# Large structural variations in the haplotype‐resolved African cassava genome

**DOI:** 10.1111/tpj.15543

**Published:** 2021-11-10

**Authors:** Ben N. Mansfeld, Adam Boyher, Jeffrey C. Berry, Mark Wilson, Shujun Ou, Seth Polydore, Todd P. Michael, Noah Fahlgren, Rebecca S. Bart

**Affiliations:** ^1^ Donald Danforth Plant Science Center St. Louis MO 63132 USA; ^2^ Department of Ecology, Evolution, and Organismal Biology Iowa State University Ames IA 50011 USA; ^3^ The Molecular and Cellular Biology Laboratory The Salk Institute for Biological Studies La Jolla CA 92037 USA

**Keywords:** cassava, genome assembly, high heterozygosity, haplotype phasing, structural variants

## Abstract

Cassava (*Manihot esculenta* Crantz, 2*n* = 36) is a global food security crop. It has a highly heterozygous genome, high genetic load, and genotype‐dependent asynchronous flowering. It is typically propagated by stem cuttings and any genetic variation between haplotypes, including large structural variations, is preserved by such clonal propagation. Traditional genome assembly approaches generate a collapsed haplotype representation of the genome. In highly heterozygous plants, this results in artifacts and an oversimplification of heterozygous regions. We used a combination of Pacific Biosciences (PacBio), Illumina, and Hi‐C to resolve each haplotype of the genome of a farmer‐preferred cassava line, TME7 (Oko‐iyawo). PacBio reads were assembled using the FALCON suite. Phase switch errors were corrected using FALCON‐Phase and Hi‐C read data. The ultralong‐range information from Hi‐C sequencing was also used for scaffolding. Comparison of the two phases revealed >5000 large haplotype‐specific structural variants affecting over 8 Mb, including insertions and deletions spanning thousands of base pairs. The potential of these variants to affect allele‐specific expression was further explored. RNA‐sequencing data from 11 different tissue types were mapped against the scaffolded haploid assembly and gene expression data are incorporated into our existing easy‐to‐use web‐based interface to facilitate use by the broader plant science community. These two assemblies provide an excellent means to study the effects of heterozygosity, haplotype‐specific structural variation, gene hemizygosity, and allele‐specific gene expression contributing to important agricultural traits and further our understanding of the genetics and domestication of cassava.

## Significance Statement

The cassava varieties grown by subsistence farmers in Africa largely differ from the partially inbred reference genome due to their highly heterozygous nature. We used multiple sequencing technologies to assemble and resolve both haplotypes in TME7, a farmer‐preferred cassava line, enabling us to study the considerable haplotypic structural variation in this line.

## INTRODUCTION

Cassava (*Manihot esculenta* Crantz 2*n* = 2*x* = 36) is a globally important crop and is particularly critical for subsistence farmers in the developing world (Ceballos et al., 2004). As an outcrossing plant, cassava is considerably heterozygous with a high genetic load and thus suffers from inbreeding depression (Rojas et al., 2009). This has hindered genetic improvement via breeding in cassava, and many agriculturally favorable lines are commonly clonally propagated, which maintains any heterozygosity in the germplasm (Aye, 2012; Ramu et al., 2017). Moreover, the heterozygous nature of the cassava genome and limitations in sequencing technologies have hindered the ability to sequence and assemble the genome accurately (Chin et al., 2016). Because of this, a partially inbred cassava accession, AM560‐2, was selected as the cassava reference genome (Prochnik et al., 2012). AM560‐2 is the product of three generations of selfing of the Colombian cassava line MCol1505, and is 94% homozygous (Bredeson et al., 2016). The reference genome has been an asset to the cassava community for more than 10 years, but due to the homozygous nature of the genome it does not accurately represent lines grown in farmer’s fields.

The development of long‐read and long‐range sequencing technologies and recent advancement in assembly algorithms have strong implications for genome assembly of heterozygous plant and animal species. Such haplotype‐resolved genome assemblies can be crucial to our comprehension of genetics in crops with strong inbreeding depression where generation of inbred lines is very difficult and not representative of the agriculturally grown plants. However, even with these advances, assembling fully haplotype‐phased genomes is difficult, particularly when rates of heterozygosity are high (Michael and VanBuren, 2020). New genome assembly strategies now exist for separate assembly of homologous and homeologous chromosome copies, allowing for accurate phasing of haplotypes and polyploid genomes (Chin et al., 2016; Koren et al., 2018; Kronenberg et al., 2018). One such strategy uses sequence data from parental lines to discern the haplotype specificity of offspring sequence reads before their assembly (Koren et al., 2018). However, this strategy requires access to the parental genotypes, which are unknown in many clonally propagated farmer‐preferred cassava lines. Another novel approach utilizes single cell sequencing of gamete cells to gain insight into phasing information and haplotype assembly (Campoy et al., 2020). This “Gamete binning” approach was showcased in the heterozygous tree crop apricot (*Prunus armeniaca*), and while potentially a viable option for field grown cassava lines, it requires extraction of pollen nuclei and other technical skills that are potentially limiting factors to its immediate adoption (Campoy et al., 2020). An alternate computational approach, implemented in the FALCON‐Phase algorithm, uses mapping information from long‐range chromatin conformation capture (Hi‐C) sequencing to phase haplotype assembled sequences correctly (Kronenberg et al., 2018). This *de novo* approach can be used to correct assembly phase switch errors, and accurately represent the chromosome from telomere to telomere (Kronenberg et al., 2018).

Recent attempts at assembling heterozygous farmer‐preferred cassava lines have produced contiguous large assemblies (Kuon et al., 2019). These assemblies, however, are limited due to the lack of haplotypic separation; the primary assemblies include both haplotypes and thus contain many duplicated sequences (Kuon et al., 2019; Lyons et al., 2021). This has implications on the assembly size and scaffolding, which can be severely impacted by these duplications (Guan et al., 2020). Sequence duplication can also cause problems for downstream analyses such as read mapping and gene annotation. Assessing the deduplication, completeness, and quality of heterozygous genomes thus plays a critical role in each assembly step, to ensure truly resolved haplotypic sequences (Rhie et al., 2020).

Here, we assemble a phased diploid assembly of the Nigerian cassava landrace (Tropical‐*Manihot*‐*esculenta*) TME7, also known as "Oko‐iyawo," a farmer‐preferred line resistant to the cassava mosaic disease virus (Rabbi et al., 2014). By assembling and phasing the moderately sized (approximately 700 Mb) diploid cassava genome we have a unique opportunity to study haplotype‐specific structural polymorphisms maintained for generations by clonal propagation. Elucidation of haplotype‐specific structural variations (SVs) in cassava will have direct implications for our understanding of these types of variations in other clonally propagated, heterozygous crops with larger genomes, including many tree fruit crops and other horticulturally important species. The two haplotype assemblies will also provide an excellent means to study the haplotype‐specific SVs, synteny, and allele‐specific gene expression that contribute to important agricultural traits, furthering our understanding of the genetics and domestication of cassava. As breeding is difficult in a crop such as cassava, a better understanding of the haplotype‐specific genetics will allow for more accurate, appropriate, and targeted gene editing to improve lines for agricultural purposes.

## RESULTS AND DISCUSSION

### Genome size and heterozygosity

**Figure 1:**
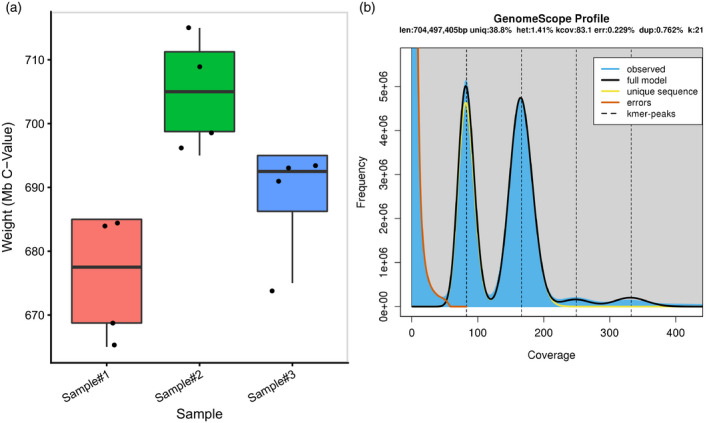
Estimates of TME7 genome parameters using flow cytometry and short reads. (a) Three biological samples of TME7, each with four technical replicates, were analyzed using flow cytometry. A mean genome size was estimated at 690 Mb. (b) Estimation of genome size, heterozygosity, and repetitiveness using GenomeScope Profile. *K*‐mer size was set to 21, and *k*‐mer coverage cutoff was set at 1e6 to include repeat regions in genome size estimates. The haploid genome size was estimated to be 704 Mb consisting of 61% repetitive sequence and a heterozygosity of 1.41%. dup, mean read duplication rate; err, percentage sequencing error rate; het, percentage estimated heterozygosity; kcov, average *k*‐mer coverage for heterozygous bases; len, haploid length; uniq, percentage non‐repetitive sequence.

Owing to the significant differences between TME7 (a clonally propagated, heterozygous, farmer‐preferred line grown in Africa) and AM560‐2 (an inbred South American line) we opted to re‐estimate the genome size of TME7 before assembly. Both flow cytometry and a *k*‐mer based approach (GenomeScope; Vurture et al., 2017), estimated the genome size to be within the range of 670–711 Mb (Figure 1). We settled on approximately 700 Mb as a target haploid size for this assembly. This estimate is moderately lower than that estimated for the reference genome line AM560‐2 (approximately 750 Mb, Bredeson et al., 2016 ). Based on the *k*‐mer analysis, the repeat content was estimated at roughly 61% of the estimated genome size and the two very distinct *k*‐mer frequency peaks suggested a high level of heterozygosity (Figure 1b; Figure S1). The GenomeScope model further estimated the heterozygosity of this cassava line to be approximately 1.4%, or roughly one polymorphism every approximately 70 bp (Figure 1b). This is slightly lower than other outcrossing clonally propagated crops such as pear (1.6%; Vurture et al., 2017), grape (1.6–1.7%; Guan et al., 2020; Patel et al., 2018), as well as the closely related rubber tree (1.6%; Liu et al., 2020). Nonetheless, this level of estimated heterozygosity suggested that haplotype‐resolved assembly approaches would be appropriate for assembly of the cassava genome.

### Maximizing the diploid assembly

With that goal in mind, we sequenced the TME7 cassava genome using long‐read PacBio single‐molecule real‐time sequencing cells yielding roughly 90× coverage. We generated 64.2 Gb of data in 8 018 064 raw PacBio subreads (Figure S2) that had an N50 of 11 099 bp; 4 970 318 of the reads were longer than 5000 bp, which was used as a seed read size. After multiple assembly attempts with different software (i.e., Canu) and other parameters (Table S1), we generated a PacBio‐only assembly with FALCON and FALCON‐Unzip (Chin et al., 2016), which yielded the best compromise between longest and most contiguous results. FALCON‐Unzip assembled a total of 874 Mb in primary contigs, as well as an additional 157 Mb in haplotigs. FALCON‐Unzip is limited in its ability to identify sequences with greater than 4–5% variation as haplotypic sequences, and these are often retained as primary contigs (Chin et al., 2016, also e.g., Padgitt‐Cobb et al. (2021)). The total sequence assembled was approximately 1 Gb, and while not yet well partitioned into haplotypes, included about 300 Mb in potentially haplotypic sequences. This represented the potential for an approximately 50% “unzipped” genome assembly. Assembly statistics for each stage of assembly and phasing are reported in Table 1.

**Table 1:** Assembly contiguity, completeness, and quality assessment

	Falcon		Falcon‐Unzip		Pilon		Add SRC	purge_dups		FALCON‐Phase Unzip		FALCON‐Phase Pseudohaplotype		Scaffolded
	Primary	Alternate	Primary	Alternate	Primary	Alternate	Diploid	Primary	Alternate	Primary	Alternate	Primary	Alternate	Primary
No. contigs	6910	917	5114	5254	5114	5254	75 291	9925	17 415	9925	12 805	9925	9925	4936
Total length (Mb)	901	50.3	874	157	875	157	115	677	341	702	313	720	720	720
N50 (kb)	253.3	76.1	263.3.	51.6	263.4	51.6	192.4	283.1	83.0	305.2	80.3	318.9	322.7	31.2 Mb
Completeness	83.3	10.2	86.1	28.6	87.5	29.0	‐	81.2	56.5	82.0	54.2	82.8	82.8	82.8
Both	84.6		90.05		91.5		96.2	93.9		93.65		93.66		
QV	27.5	28.5	29.7	29.5	33.3	32.7	‐	34.3	33.5	34.1	34.4	34.0	34.0	34.0
Both	27.56		29.68		33.18		33.66	34.03		34.2		34.0		

QV, phred scaled quality score; SRC, short‐read contigs.

To estimate the success of haplotypic separation and assembly quality we performed *k*‐mer based analyses using Merqury (Rhie et al., 2020). Using raw, highly accurate short read sequencing representing data from both haploid sequences, *k*‐mers, which exist in one or two copies arise from heterozygous and homozygous regions, respectively. The *k*‐mer distributions are then represented by the number of times each *k*‐mer appears in the assembly allowing for the comparison of observed and expected coverage, estimation of reference‐free completeness, and overall phasing success.

We first observed that even after polishing INDELs with pilon (Walker et al., 2014), a peak of heterozygous (1‐copy) *k*‐mers are missing from either the primary or alternate assemblies (Figure S3). As our goal was to assemble a full heterozygous diploid phased assembly, we sought to maximize the amount of haplotypic sequence assembled. To this end, we supplemented the long‐read assembly with short‐read contigs (SRC) containing additional heterozygous sequence. We identified *k*‐mers that contained the short reads pertaining to the missing heterozygous sequence and assembled them using SPAdes (Bankevich et al., 2012). Some of these extra SRC contained duplicates of already assembled sequences, but importantly many included the missing heterozygous sequence. Adding these SRCs to the full assembly brought the total assembled sequence to 1.15 Gb, or nearly the anticipated diploid size of approximately 1.4 Gb. The number of missing *k*‐mers was brought down from 23.7 to 9.9 m using this approach and the “Completeness” score was brought up to 96.2% when including the SRCs (Table 1). Based on the missing *k*‐mers, after adding the SRC an estimated 9.8 Mb of missing heterozygous sequence remained un‐assembled.

### Haplotypic purging and deduplication

**Figure 2:**
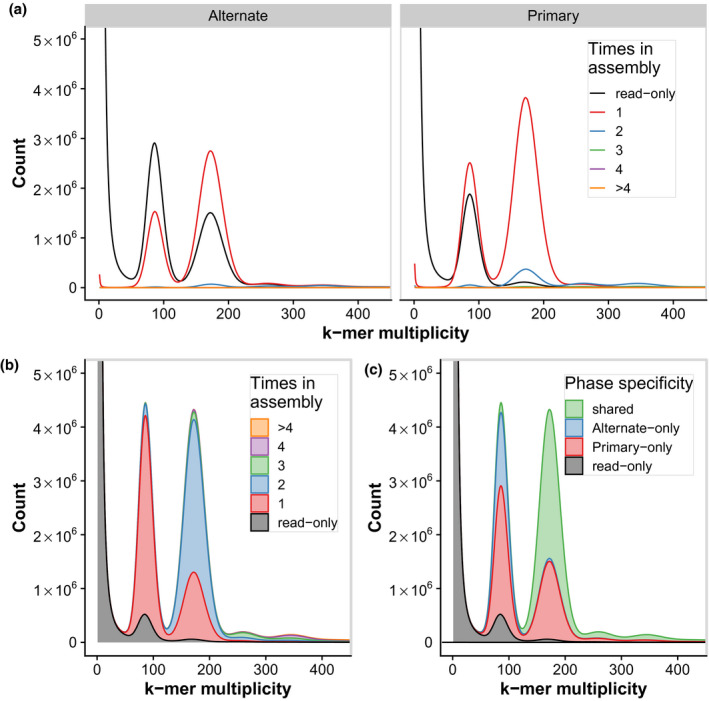
*K*‐mer copy number and assembly analyses for the final phased TME7 assemblies. (a) *K*‐mer count spectra for the alternate (haplotigs) and primary assemblies after phasing. (b) Diploid (primary + haplotigs) *k*‐mer count spectra. In both (a) and (b), short read *k*‐mer distribution plots are colored by the number of times a *k*‐mer is present in the assembly. *K*‐mers denoted in gray are missing from the assembly and represent probable short‐read sequencing errors (*k*‐mer multiplicity <50) or missing assembled sequence (≥50). (c) Assembly spectra of the diploid assembly suggest that most homozygous *k*‐mers (approximately 200× peak) are shared between the assemblies, while most of the heterozygous (approximately 100× peak) *k*‐mers are phase specific.

To complement the graph‐based assembly approach used in FALCON‐Unzip (Chin et al., 2016), other orthogonal tools have since been developed to extract haplotypic sequences from primary assemblies (e.g., Guan et al., 2020; Huang et al., 2017; Roach et al., 2018). These typically use read mapping coverage and sequence homology to identify potential haplotigs and “purge” them from the primary assembly (Roach et al., 2018). After maximizing our diploid assembly size to include as much haplotypic sequence as possible, our goal was to purge the primary assembly of haplotypic contigs, overlaps and sequence duplication, including those from our SRCs (Figure 2; Figure S3). To this end we used purge_dups (Guan et al., 2020), which improves on the previous state‐of‐the‐art, purge_haplotigs (Roach et al., 2018), by identifying and purging haplotypic overlaps. The final set of primary contigs included approximately 677 Mb assembled in 9925 contigs with an N50 of 283.1 kb. The resulting alternate assembly contained over 341 Mb assembled in haplotigs, representing an approximately 50% “unzipped” genome.

**Figure 3:**
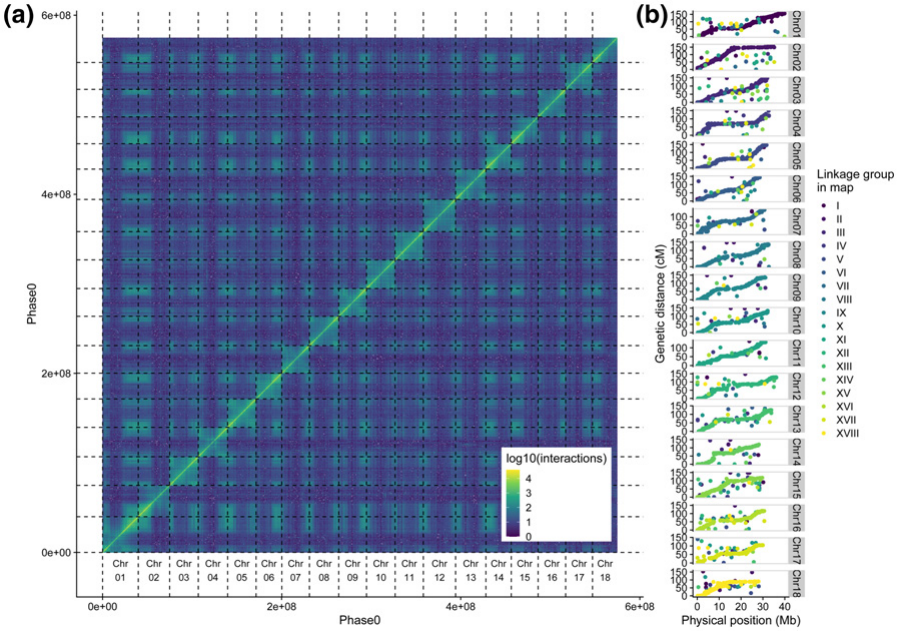
Validation of Hi‐C scaffolding order and orientation by contact map and linkage map alignment. (a) Post‐scaffolding Hi‐C contact heatmap of the 18 largest scaffolds in the Phase0 assembly of TME7 showing the density of Hi‐C interactions between regions of the genome. Color represents the intensity of interactions between regions, reported in log(1 + *x*). (b) Strong collinearity between the 22K marker Cassava Linkage Map and the TME7 Phase0 assembly. Markers are colored by their originating linkage group in the map.

**Figure 4:**
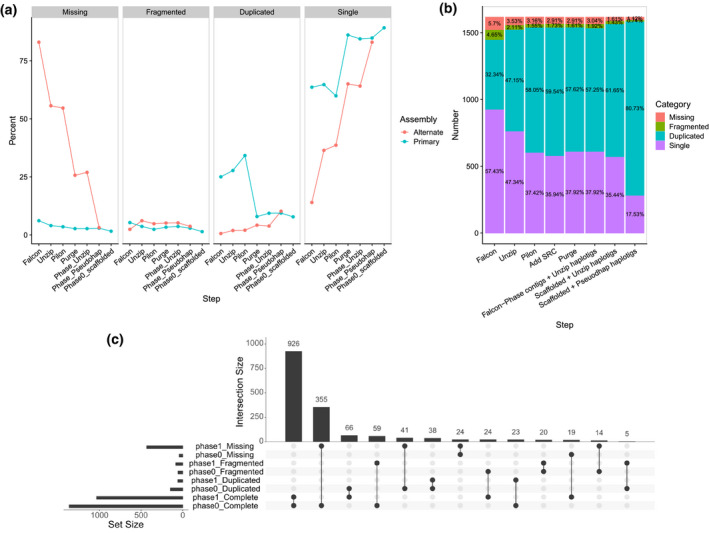
Summary of BUSCO analyses and phase‐specific BUSCOs. The BUSCO scores for each step are reported for the (a) alternate or primary assemblies or (b) full (concatenated) assemblies. (b) After polishing with Pilon, short‐read contigs (SRC) were assembled and added to the full assembly, before haplotypic purging. (c) Overlap in BUSCO categories of the final FALCON‐Phase Unzip‐emit primary (Phase0) and alternate (Phase1) assemblies shows that most BUSCOs are phased and exist in both assemblies.

*K*‐mer spectra plots showed that the amount of sequence duplication was drastically reduced after purging, and that most of the heterozygous (1‐copy) *k*‐mers were now successfully separated into the two assemblies (Figure 2; Figure S3). This was further confirmed by alignment of markers from the cassava 20k linkage map (ICGMC, 2015) (Figure 3b) and deduplication of BUSCO genes (Figure 4). After purging, the haplotig N50 size, which corresponds to the haplotype phase block, was 83 kb (90.5 kb if excluding SRC‐derived haplotigs). This is substantively smaller than the 7‐Mb block described in the Arabidopsis F_1_ assembly by Chin *et al*. (2016), but is more similar to that observed in the Carménère grape (89.5 kb, Minio et al., 2019). Furthermore, it is consistent with relatively short dispersed regions of heterozygosity, and with the high rate of linkage disequilibrium decay described in cassava, an obligate out crosser (Ramu et al., 2017).

### Haplotype phasing and scaffolding with Hi‐C sequencing

To get a more accurate representation of the TME7 pseudo‐haplotypes, we phased the primary and haplotig assemblies using Hi‐C data and FALCON‐Phase (Kronenberg et al., 2018). We noticed, however, that during the placement and mincing stages of the algorithm, FALCON‐Phase was discarding over 40 Mb of sequence from both primary and haplotig assemblies. We compared the haplotig truncation lengths with the contig vs. haplotig alignment lengths and identified that the FALCON‐Phase *coords2hp.py* script truncated both contigs and haplotigs at the ends of alignments. We hypothesized that if large SVs exist between haplotypes, this could affect how FALCON‐Phase aligns and places haplotigs vs. their primary contigs. Owing to these large SVs between the haplotypes, haplotig sequences were truncated to exclude the non‐aligning sequences. Merqury analysis showed that removal of these sequences reduced the number of heterozygous *k*‐mers in the assembly (Figure S4). We thus modified the *coords2hp.py* script in FALCON‐Phase to force it to include the entire length of each haplotig, rather than only the length of the sequences that aligned.

The result was one complete set of 9925 contigs comprising approximately 720 Mb for each phase, which included almost all the original heterozygosity assembled. This suggests we were able to assemble almost the entirety of the TME7 genome successfully (720 Mb haploid assembly vs. approximately 700 Mb estimated genome size) with a contig N50 of approximately 320 kb for both assemblies. When emitted in the “unzip” format, the total primary and haplotig contig length was 702 Mb (N50 = 305 kb) and 311 Mb (N50 = 80 kb), respectively. We assessed the success of the phasing step using Merqury (Figure 2). A modest increase in homozygous sequence duplication was observed after phasing, probably due to incorporation of homozygous contig boundaries into the primary assembly (Figure 2a). This minor sequence duplication in the “pseudohaplotype” assembly was also observed with the unmodified version of the *coords2hp.py* script suggesting it may be an inherent issue with the FALCON‐Phase algorithm (Figure S4). While this additional minor duplication is a limitation with this phase correction approach, the benefits of accurate phasing outweigh this issue.

After phasing, the Hi‐C data were further used to scaffold the assembly into 18 chromosome length scaffolds. Contigs designated as part of Phase0 were scaffolded using the Proximo algorithm (Phase Genomics) and manual scaffolding curation with Juicebox (Durand et al., 2016; Rao et al., 2014). This process resulted in placing approximately 80% of all sequences in a set of 18 chromosome‐scale scaffolds containing 580 Mb of sequence (Figure 3a). We validated the scaffolding order and orientation by aligning 22 403 single nucleotide polymorphism (SNP) markers from the cassava composite map (ICGMC, 2015) to both phases. After filtering for >95% identity and >150 bp length, >19 000 markers aligned uniquely to both phases. We plotted the concordance between the new *de novo* assembly and the linkage map and observed high collinearity between the two (Figure 3b). Except for a few cases, there was high agreement between the physical and linkage maps (average Spearman’s correlation of 0.96). Approximately 1900 marker sequence tags had duplicate mapping sites on the same scaffold in both phases and were distributed along the chromosome scaffolds (Figure S5). While this may suggest potential sequence duplication or retained heterozygosity, an alternate explanation for some of these duplications are genotype‐specific duplications in TME7 that differ from the inbred reference genome. This represents a significant improvement over the previous attempts at assembly of heterozygous African cassava lines (Kuon et al., 2019), where close to 30% of markers had multiple map hits, indicating a not well deduplicated assembly.

### Assessing the quality of the final assembly

When compared with the raw diploid short read data, the final assemblies showed approximately 94% completeness and a phred scaled quality score (QV) of >33 (or >99.9995% accurate) (Table 1). More short‐read polishing could be performed to increase accuracy; however, this might come at a cost of falsely correcting heterozygosity. While some heterozygous sequences are still missing from the assembly, the majority of 1‐copy *k*‐mers are uniquely assigned to one of the haploid assemblies and not shared between them (Figure 2c). These results show that we have accurately produced one full haplotype assembly of TME7 and a second alternate assembly that contains most of the haplotypic variation in this genotype.

We used BUSCO (Simão et al., 2015) analysis to verify that we successfully resolved the TME7 haplotypes (Figure 4). The primary (Phase0‐scaffolded) assembly had a complete BUSCO score of 96.9%, marginally outperforming the AM560‐2 v6.1 assembly (complete: 95.1%; duplicated: 5.1%) (Bredeson et al., 2016). The majority of complete single BUSCOs (969) are assembled in both phases, yet another 374 are missing from the alternate assembly (Figure 4c). This could be because these BUSCOs are homozygous and thus assembled in the collapsed regions of assembly, and/or due to the missing heterozygosity. Importantly, our deduplicated, TME7 Phase0 assembly only contains 7.9% duplicated BUSCOs, which is comparable with that of AM560‐2 and represents a significant improvement compared with approximately 15% and approximately 19% of the non‐haplotype‐purged assemblies described in Kuon et al. (2019). Interestingly, we identified haplotype‐specific complete BUSCOs (Figure 4c), and together the full diploid assembly (Phase0 scaffolds + Phase1 pseudohaplotype contigs) has a complete BUSCO score of greater than that of each phase separately (complete: 98.2%; 80.7% duplicated). This indicates that some BUSCOs may exist in a hemizygous state in the TME7 genome, and that complementation between the phases preserves the existence of these potentially crucial single copy genes.

### Transposable elements and gene annotation

#### Transposable element and repeat annotation

Assembling the repetitive portion of the cassava genome is challenging as it is predicted to contain about 60% repetitive sequence (Figure 1b; Figure S1). We used the long terminal repeat (LTR) Assembly Index (LAI) to assess the quality and contiguity of the repetitive sequence assembly (Ou et al., 2018). Overall, both haploid assemblies display reference‐quality contiguity in the repetitive portions of the genome, with LAI values of 10.53 and 11.17 for the Phase0 and Phase1 assembly, respectively (Figure S6a). Furthermore, we found that the contiguity of the repetitive space in the assembly was much improved compared with the unplaced scaffolds (Figure S6b). We annotated both structurally intact and fragmented transposable elements (TEs) in the full diploid assembly using EDTA (Ou et al., 2019). As expected, 59% of the TME7 genome are repeats and TEs, which are dominated by LTR retrotransposons that contribute about 50.5% of the genome (Table 2; Figure S7). Terminal inverted repeat and Helitron DNA transposons contributed 2.43% to the total genome size. There were only marginal differences in TE content between the phases.

**Table 2:** Summary of TEs in the TME7 genome assembly

Category	Phase0, %	Phase1, %	Average, %
LTR/Copia	6.24	6.25	6.25
LTR/Gypsy	35.36	36.22	35.79
LTR/unknown	8.49	8.46	8.48
TIR/CACTA	0.64	0.63	0.64
TIR/Mutator	0.90	0.88	0.89
TIR/PIF_Harbinger	0.13	0.16	0.15
TIR/Tc1_Mariner	0.01	0.01	0.01
TIR/hAT	0.77	0.67	0.72
LINE/unknown	0.44	0.44	0.44
DNA/Helitron	0.02	0.03	0.03
Repeat/unknown	6.05	5.45	5.75
Total LTR	50.09	50.93	50.51
Total DNA TE	2.47	2.38	2.43
Total TE	59.06	59.19	59.13

LTR, long terminal repeat; TE, transposable element; TIR, terminal inverted repeat.

#### Gene annotation, synteny, and tandem duplication

**Figure 5:**
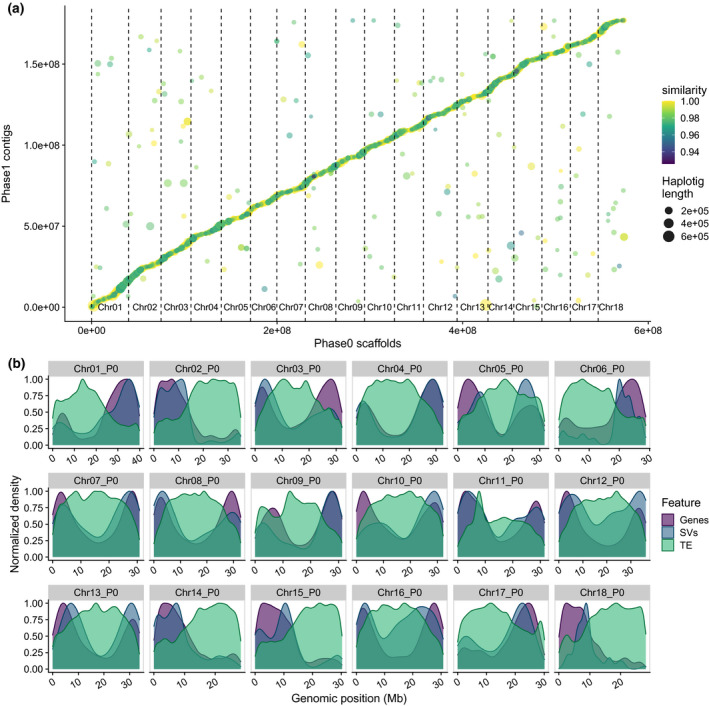
Comparison of the TME7 haplotype phased assemblies. (a) Dot plot of the best sequence alignments of the two haplotype assemblies. Color represents the alignment percentage identity between the alternate assembly (Phase1) contig (haplotig) and the primary assembly (Phase0). (b) Chromosomal distribution of annotated genes, transposable elements (TE) and large haplotypic structural variants (SVs) between the two phased assemblies. SVs were identified by sequence alignment of the two phases. P0, Phase0 assembly.

**Figure 6:**
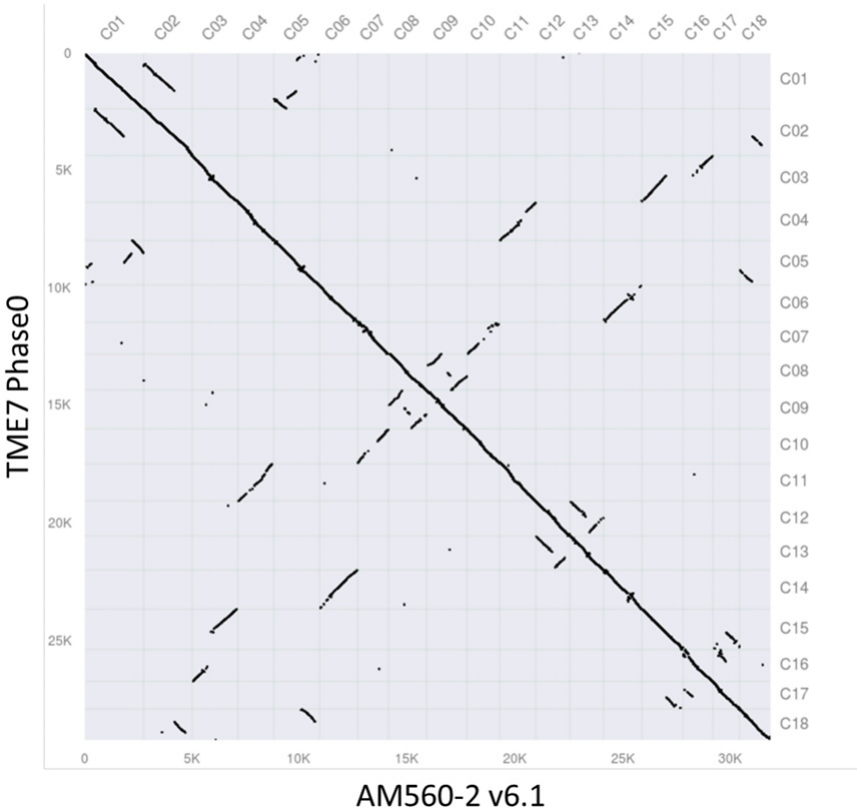
Macro‐synteny between of the TME7 and the AM560‐2 Ref6.1 genome. Gene synteny dot‐plot comparison between the scaffolded TME7‐Phase0 assembly and the AM560‐2 reference genome shows largely co‐linear genomes with multiple inter‐chromosomal duplications attributable to the paleotetraploidy described in cassava in Bredeson et al. (2016).

Gene annotation was performed using the MAKER, AUGUSTUS, and SNAP pipelines, including transcript evidence from RNA‐sequencing (RNA‐seq) from 11 tissue types (Wilson et al., 2017). We annotated 33 653 and 35 684 genes in Phase0 and Phase1 assemblies, respectively (Figure 5). Over 70% of annotated genes had an Annotation Edit Distance of <0.25 suggesting most genes were supported by high evidence levels (Figure S8a). Comparison of our annotations with that of the AM560‐2 ref v6.1 showed that gene synteny between the two cassava genomes was largely conserved; however, several macro‐level rearrangements are identifiable (Figure 6). Furthermore, this comparison revealed a largely 2:2 pattern of syntenic depth between the annotations (Figure S9), consistent with the whole genome duplication described in cassava (Bredeson et al., 2016). About 36% of cassava genes exist in one syntenic block reciprocally in either genome, suggesting that these genes may have lost their extra copy since the paleo‐duplication. Based on our analysis, it thus appears that the percentage of genes, which have retained their duplicate status is closer to 60%, rather than approximately 36% as previously reported (Bredeson et al., 2016). The previous analysis used homologous genes identified in *Jatropha curcas* as the reference; this likely limited the total numbers of homologs in the analysis, leading to the underestimate of retained duplicated genes. Only 2% of AM560‐2 genes were not shared in syntenic blocks in TME7 suggesting they may be unannotated, lost, or translocated out of their block.

We examined and compared the number of tandem duplications (arrays) between the two TME7 phase annotations. We identified 1256 arrays in Phase0 containing 2,865 genes and 1,159 arrays containing 2,608 genes in Phase1. While most arrays were of two tandem genes (approximately 1000 in both phases), array sizes varied to 11 and 8 genes in an array in Phase0 and Phase1, respectively (Figure S8b). As expected, genes in tandem arrays were associated with Gene Ontology (GO) terms of important defense‐related biological processes, for example: “oxidation‐reduction process,” “response to other organism,” and “phenylpropanoid biosynthetic process” (Table S2). In synteny analyses between the annotations of the two phases, we identified that approximately 20% of genes in Phase0 were no longer in syntenic blocks in Phase1 (Figure S9). This could be due to specific gene deletion, or alternatively, may reflect the contig format of Phase1, which disrupts the contiguity of the synteny blocks. This, together with gene duplication arising from the evident whole genome duplication limits the ability to identify accurately which genes are haplotype specific and missing from one annotation; however, we have supplied the MCScanX (Wang et al., 2012) gene anchor file as Supporting Information to help users of the genome identify probable best hits between genes of the two phases (File S1). Further exploration of gene hemizygosity in cassava will be interesting from a genome evolution standpoint and have important implications for breeders.

### Haplotype‐specific sequence and SV

#### Comparison with the inbred AM560‐2 reference

**Figure 7:**
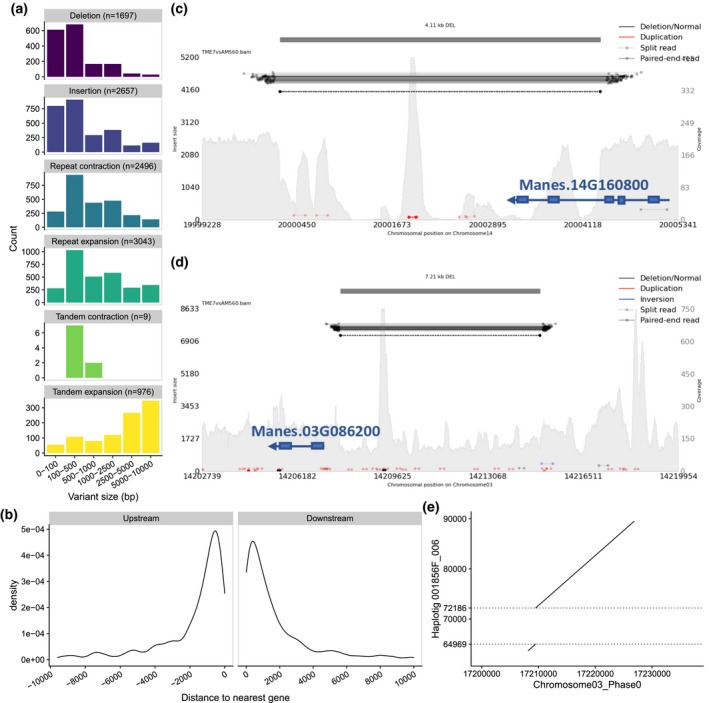
Large structural variants identified in TME7 vs. the AM560‐2 reference genome. (a) Size distribution histograms of structural variants identified by comparison of the Phase0 assembly to the AM560‐2 reference genome. (b) Density of distances (<10 kb away) of large deletions (50–10 000 bp) in TME7 from genes annotated in the AM560‐2 reference. (c,d) Structural variants interrogated by paired‐end reads. Reads with abnormally large insert sizes (color‐coded horizontal bars, left *y*‐axis) corroborate deletions identified by alignment of the assemblies. The depth of coverage (gray‐filled background, right *y*‐axis) aid in determining the zygosity of the deletions. Gene models from the AM560‐2 v6.1 annotation are in blue. (c) TME7 Phase0 assembly contains a homozygous 4.11 kb deletion compared with Chromosome14 of the AM560‐2 Reference genome which overlaps the 3′‐end of *Manes.14G160800*. (d) A 7.21‐kb heterozygous deletion is verified on Chromosome3, potentially overlapping with the upstream regulatory region of *Manes.03G086200*. Other smaller sequence duplications are also observable (marked in red in c and d). The 7.21 kb heterozygous deletion in TME7 is correctly phased and assembled as an insertion in haplotig 001856F_006. (e) Deletion between 64.9 kb and 72.1 kb on the haplotig, is delineated between the two dashed horizontal lines.

The differences in origin, genome size, and levels of heterozygosity between TME7 and the reference line AM560‐2, prompted us to compare the assemblies. Comparison of the TME7 Phase0 assembly to the AM560‐2 ref v6.1 assembly revealed 2 257 216 SNPs and 1 666 639 bases affected by small INDELs (<50 bp) that differed (Figure S10). We further identified over 10 000 large SVs (50–10 000 bp) affecting more than 15.99 Mb of sequence (Figure 7a; File S2). There is increasing evidence pointing to the importance of large genomic SVs, and their contribution to phenotypic traits (Alonge et al., 2020; Zhou et al., 2019). We examined the potential effects of the large INDELs (>50 bp) on gene function by measuring the distance to the nearest genes (Figure 7b). Of 4,354 large INDELs, 1,217 were predicted to be within gene models and another 882 within 2,000 bp upstream of genes, potentially affecting *cis*‐regulatory elements.

To validate and assess visually the heterozygosity state of several of the largest deletions (>4 kb in length), we aligned short reads from TME7 to the AM560‐2 genome. Both homozygous and heterozygous deletions were identified, and an example of each is in Figure 7c and d, respectively. A homozygous deletion identified on Chromosome14, where paired‐end reads map to either side of the 4.11‐kb deletion and a sharp decline in read coverage is observed, overlaps with the 3′‐end of RNA CLEAVAGE STIMULATION FACTOR (Manes.14G160800) (Figure 7c). A heterozygous deletion on Chromosome03, that has read coverage approximately half that of the surrounding area, overlaps the potential promoter region of Manes.03G086200, annotated to encode Ribosomal protein L6 (Figure 7d). This further supports the importance of assembling both haplotypes and suggests that many large haplotypic SVs might be present with potential impact on gene expression or function.

#### Large haplotypic SVs in TME7

Recently shown in grape (Zhou et al., 2019) and tomato (Alonge et al., 2020), large genomic SVs may have substantive effects on important agricultural traits. For example, the white berries of Chardonnay grape could be a result of a large inversion and deletion, causing hemizygosity at the *MybA* locus (Zhou et al., 2019). To examine further the within‐genome, haplotypic variation in TME7 we aligned the alternate assembly to the primary assembly. FALCON‐Phase has two options for emitting phased assemblies. In “unzip” style, short haplotigs containing alternate sequences are emitted alongside the phased primary contigs (as in FALCON‐Unzip). In contrast, in “pseudohap” mode, pseudo‐haplotype contigs are generated by collapsing alternate sequences from the phased haplotigs with homozygous sequence from primary assembly. Thus, the pseudo‐haplotype alternate assembly might contain artificially homozygous sequences that were missing from the original alternate assembly, originating from lack of assembly or true hemizygosity in the alternate assembly. We therefore used the “unzip”‐emit‐style haplotigs for comparison with the primary assembly and calculated the mean haplotype divergence to be 2.09 ± 0.18%. We further identified 1 116 832 SNPs and 300 883 small INDELs (<50 bp) in non‐repetitive regions, collectively representing more than 2.14 Mb of heterozygous sequence between the two assemblies (Figure 5a). This confirms the high rate of heterozygosity predicted using *k*‐mer based approaches and suggests a well extracted set of haplotigs.

To compare directly the two independently assembled TME7 haplotypes, we aligned the Phase1 contigs to the scaffolded Phase0 assembly and identified large SVs. Overall, we identified more than 5000 variants 50–10 000 bp in size including large insertions, deletions, tandem duplications, and contractions as well as repeat expansions and contractions (Table 3, Figure 5b; File S3). The total sequence space that was affected by these SVs was greater than 8 Mb. Thus, this within‐genotype, haplotypic SV amounts to greater than half of the between‐genotype differences that TME7 has with the AM560‐2 reference line. The Assemblytics pipeline can also identify variants >10 kb; however, the accuracy with which these are distinguished from translocations or assembly errors is limited (Nattestad and Schatz, 2016). Though we primarily focused on a more conservative approach to identify large SVs, potentially larger haplotypic SVs were identified using Assemblytics; including SVs up to 50 kb in size in the analysis, yielded close to 16 Mb of sequence affected by SV (Figure S11). While these larger SVs should be considered with caution, we note that this is comparable with structural heterozygosity reported in other species such as wine‐grape (Minio et al., 2019).

**Table 3:** Summary of haplotype‐specific structural variants

	50–500 bp Count	50–500 bp Total bp	500–10 000 bp Count	500–10 000 bp Total bp	Total count	Total bp
Insertions	699	110 936	348	791 434	1047	902 370
Deletions	676	99 453	226	663 975	902	763 428
Repeat expansion	649	139 116	938	2 722 146	1587	2 861 262
Repeat contraction	668	144 659	1136	3 486 466	1804	3 631 125
Tandem expansion	27	5575	31	125 712	58	131 287
Tandem contraction	7	819	3	5070	10	5889
				Total:	5408	8 295 361

### Effects of haplotypic SV on allele‐specific expression

The identified haplotypic SVs are primarily distributed in the chromosome arms and thus are often near genes (Figure 5b). For example, the 7217 bp heterozygous deletion, upstream of *Manes.03G086200* (Figure 7c) is correctly phased in our assemblies, as it was detected as an insertion in the Phase1 contigs by alignment of the Phase1 contigs vs. the Phase0 scaffolds (Figure 7e). We posited that large haplotype‐specific INDELs (>50 bp) in the vicinity of genes, such as this one, could impact their allele‐specific expression (ASE). We examined ASE patterns, and their relation to SVs, in previously published RNA‐seq data from 11 cassava tissues harvested from TME204, another heterozygous farmer preferred line (Wilson et al., 2017).

**Figure 8:**
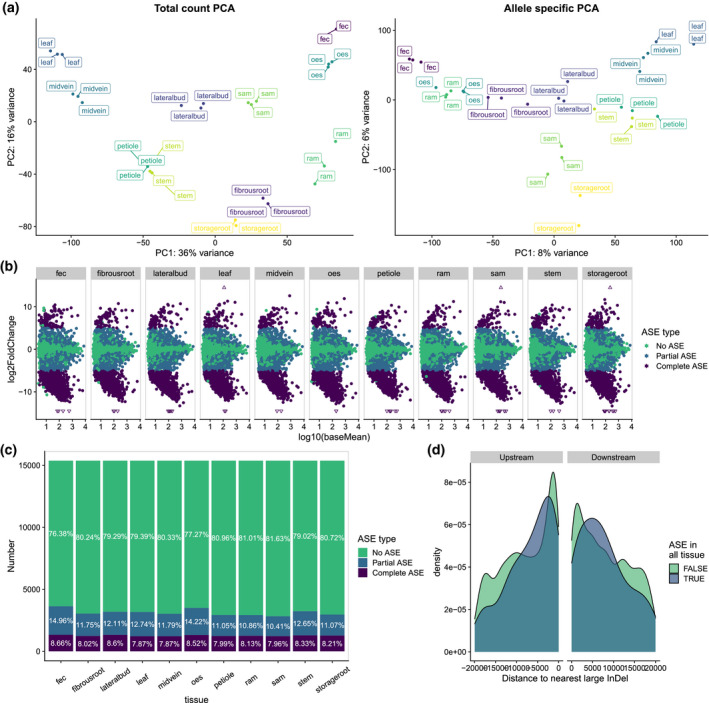
Potential effects of large haplotypic structural variants on allele‐specific expression. (a) Principal components analyses based on total read count (left) and the log2(fold‐change) between allele‐specific read counts (right). (b) MA‐plots of allele‐specific expression (ASE) patterns in cassava RNA‐sequencing data (Wilson et al., 2017). Each point represents an expressed gene and its average read count vs. the log2(fold‐change) between allele‐specific read counts. If a gene shows >5 log2(fold‐change) between allelic read counts the gene is characterized as having “complete ASE” (purple). Genes showing significant ASE but allelic log2(fold‐change) <5 are categorized as “partial ASE” (blue). If no significant ASE (adjusted *P* > 0.05) was observed, genes are denoted in green. (c) Percentage of genes in each category in each tissue assayed. (d) Distribution of distances to the nearest large insertion or deletion (InDel) in genes that show “complete ASE” in all tissues vs. the rest of the transcriptome.

We observed that on average, in any given tissue, approximately 20% of genes showed significant ASE (BH adjusted *P* < 0.05, Figure 8; File S4). However, of the 15 371 expressed genes detected, nearly half (7222 genes) showed significant ASE in at least one tissue. For comparison, in the analyses of above‐ground tissue of hybrid rice, over 3000 genes showed patterns of ASE (Shao et al., 2019). Genes with ASE in hybrid rice were associated with multiple biological processes (Song et al., 2013) and were potentially implicated in contributing to the genetic basis of heterosis (Shao et al., 2019). Cassava genes showing ASE were also enriched for a variety of biological process GO terms, ranging from primary metabolism to defense (Table S3). Thus, given the high levels of heterozygosity in farmer‐preferred cassava, the large number of ASE genes involved in multiple processes suggests that much of cassava physiology is under allele‐specific control. This might have important biological and agricultural implications as has been observed in other heterozygous/hybrid crops (Shao et al., 2019; Zhang et al., 2020).

As a complementary approach to visualizing ASE, we compared principal components analyses (PCA) of global gene expression to PCA of ASE differences for different tissue types. The most notable observation from this comparison was that while all root‐derived samples cluster together in the total count PCA, samples from storage root cluster with above‐ground organs, when performing the analysis based on allelic log fold‐change (Figure 8a). Future research can investigate if and how these unique ASE differences contribute to tissue identity. In total, 1441 ASE showed patterns of ASE in all tissue types, with another 2,218 genes having ASE uniquely in one tissue (Figure S12a).

While there could be multiple reasons for ASE of genes (Castel et al., 2015; Wood et al., 2015), large haplotypic INDELs in nearby or distal *cis*‐regulatory regions, such as the one in Figure 7d, could cause expression of one allele to be severely repressed. We thus defined two categories of ASE genes: we defined genes as being under “complete ASE” if significant ASE was detected and the log2(fold‐change) between alternate and reference alleles was >5. We further defined genes as having “partial ASE” if statistically significant ASE was observed, but the allelic log2(fold‐change) did not pass the threshold of 5. We observed that >24% of all genes with ASE show patterns of “complete ASE” (approximately 8% of all genes detected per tissue type) (Figure 8b).

We then compared the distribution of distances to the nearest upstream and downstream large INDEL between ASE and non‐ASE genes. In both directions relative to the genes, “complete ASE” genes had significantly different distance distributions from both “partial ASE” and “no ASE” categories (K‐S test, *P* < 0.05). Genes with “partial ASE” did not have different distance distributions compared with those with no ASE. Specifically examining the set of 847 genes that show “complete ASE” in all tissue types showed that these had an enrichment of large INDELs approximately 5,000 bp upstream and downstream the gene (Figure 8d). We further observed additional differences in more distant regions (up to 1 Mb away) as well (Figure S12b). While the genes themselves are not in a hemizygous state, there is hemizygosity in their *cis*‐regulatory regions including upstream promoter regions as well as potentially in more distal enhancer regions that may be several Mb away from the gene (Weber et al., 2016). These might have important impacts on their allelic expression and potentially on downstream phenotypes. Thus, future analyses using new approaches, such as ATAC‐seq (Buenrostro et al., 2015), might be informative to assess how the high heterozygosity and number of large haplotypic SVs impact chromatin accessible regions and how this might implicate enhancer sequences in ASE. Furthermore, examining the relationship between ASE and SVs in other datasets under additional treatments and/or conditions may further yield important cases where gene expression is affected by large haplotypic SVs (Knowles et al., 2017).

Together, the single‐nucleotide and large SVs identified by comparing the two phased TME7 assemblies open a window into the complexity of the heterozygous cassava genome. Work in grapevine and their wild relatives suggests that SVs are primarily deleterious and that they are under strong purifying selection (Zhou et al., 2019). Examining the conservation and diversity of large variants within a wide range of farmer‐preferred cassava lines would shed light on the effect of SVs on cassava genome evolution in this clonally propagated crop. Further, potentially deleterious alleles such as these large haplotypic SVs, as well as SNPs previously characterized (Ramu et al., 2017), warrant further research as these may contribute to limits in inbreeding of cassava.

### Tissue‐specific gene expression Cassava Atlas

We previously published gene expression patterns for 11 different cassava tissue types based on the AM560‐2 reference genome (Wilson et al., 2017). With our newly assembled phased genome, we updated this existing resource. All 11 RNA‐seq datasets were mapped to the Phase0 scaffolded and annotated TME7 assembly, and differentially expressed genes were identified as previously described. These results can be further explored at: shiny.danforthcenter.org/cassava_atlas.

## CONCLUSION

While recently released assemblies of farmer‐preferred cassava lines contain information from both haplotypes in the assembly, the limitation of these assemblies is in the lack of haplotypic purging and sequence deduplication (Kuon et al., 2019). Thus, these assemblies do not fully represent either of the haplotypes. Our assembly was successfully deduplicated of most haplotypic sequences, as evidenced by *k*‐mer, BUSCO, and linkage map‐based analyses. We further successfully used Hi‐C sequencing data to phase and create pseudo‐haplotype assemblies. The phased assembly described herein, is thus currently the most accurate assembly of a cassava genotype representative of those grown by millions of subsistence farmers around the world. The differences in genome size compared with the published reference (approximately 700 Mb vs. the estimated approximately 750 Mb for AM560‐2), alongside the large SVs identified between the genotypes, showcases how diversity in cassava goes beyond small nucleotide level variation between accessions. We further show that not only does TME7 have large SVs compared with AM560‐2, but that within the genome there are thousands of haplotypic SVs, potentially perpetuated through clonal variation. Many of these SVs are in close proximity to annotated genes and ASE of these genes was observed. Further research will help inform how these variants interact and affect gene hemizygosity, copy number, and expression as well as the impact on agronomically important traits. We believe this assembly will be an invaluable resource to the cassava research and breeding community, and will further aid in developing tools to ensure food security to those who rely on cassava.

## EXPERIMENTAL PROCEDURES

### Plant material and nucleic acid extraction

Cassava line TME7 (Oko‐iyawo) were obtained from Peter Kulakow at IITA in Ibadan, Nigeria. Plantlets were maintained in tissue culture by Nigel Taylor’s lab at the Donald Danforth Plant Science Center. Fresh young leaves were collected for extraction of high molecular weight DNA using a CTAB extraction method (Clarke, 2009).

### Library preparation and sequencing

#### Illumina

Data from Illumina (San Diego, CA, USA) short sequencing DNA libraries of TME7 were provided by Wilhelm Gruissem’s lab at ETH Zurich. After adapter trimming by the sequencing facility, reads were *de novo* de‐duped using Nubeam‐dedup (Dai and Guan, 2020) before further use.

#### PacBio

Initial PacBio (Menlo Park, CA, USA) sequencing was contributed by Todd Michael in 2016 and did not include size selection before sequencing. The PacBio libraries were sequenced on a PacBio RSII system with P6C4 chemistry. A second set of PacBio libraries were constructed using the manufacturer’s protocol and were size selected for 20‐kb fragments on the BluePippen system (Sage Science, Beverly, MA, USA) followed by subsequent purification using AMPure XP beads (Beckman Coulter, Brea, CA, USA). Sequencing was performed by the University of Delaware DNA Sequencing & Genotyping Center.

#### Chromatin Conformation Capture sequencing (Hi‐C)

Fresh, young cassava leaf material was sent to Dovetail Genomics (Scotts Valley, CA, USA) for DNA extraction, digestion with *Dpn*II, library preparation, and sequencing.

### Genome size and heterozygosity estimation

Flow cytometry protocol was performed at the Benaroya Research Institute at Virginia Mason in Seattle, Washington following their standard methods.

Genome size and heterozygosity were also estimated by means of *k*‐mer counting. We used jellyfish (Marçais and Kingsford, 2011) to count *k*‐mers of size 21 and plot their depth distributions from the approximately 100× paired‐end adapter‐trimmed and deduped Illumina sequencing reads of TME7. The maximum *k*‐mer depth was set to 1e6, which allows inclusion of repetitive regions of the genome. We then used the GenomeScope v1 web application (Vurture et al., 2017) to model the genome size and heterozygosity for each one of these histograms, and used the model fit to select the best *k*‐mer size for analysis.

### De novo genome assembly and scaffolding

#### Maximizing the diploid assembly

We first assembled the PacBio reads *de novo* using the FALCON and FALCON‐Unzip (Chin et al., 2016) suite of tools (v1.5.2), which included one round of consensus polishing with quiver. The config files for all FALCON tools are supplied as File S5. We further polished only INDELS with one round of Pilon (Walker et al., 2014). We identified missing heterozygous sequences using Merqury count spectra plots (Rhie et al., 2020). The *k*‐mers unique to the short reads and missing from the assembly were then extracted using the Meryl tool set (Miller et al., 2008; Rhie et al., 2020) and finally extracted the reads containing those *k*‐mers using the function meryl lookup. The short reads were first down sampled and normalized to approximately 100× coverage using BBnorm from the BBTools suite (https://sourceforge.net/projects/bbmap/) then assembled using SPAdes (Bankevich et al., 2012) and the resulting contigs were filtered for a minimum coverage depth of 10× and length of 500 bp.

#### Assembly deduplication

The complete set of assembled sequences was concatenated and processed through the purge_dups (Guan et al., 2020) pipeline. Alignment coverage histograms inform assembly purging software, such as purge_dups or purge_haplotigs (Roach et al., 2018), as to what sequences are potential haplotigs or duplication. While these software packages were developed for use with long reads, we found that short reads allow for higher resolution when plotting coverage histograms, which in turn results in more accurate sequence purging. Thus, we aligned approximately 100× deduped PE short reads to the entire diploid assembly for purging. First, duplicates, caused by retained haplotigs, haplotypic overlaps, and junk contigs, were purged from the primary assembly using manual depth cutoff settings of 5, 76, 126, 151, 252, and 453. A second round of purging on the “haplotig” output of purge_dups was useful to remove duplicates and artifact contigs created by purge_dups during purging of overlaps, again using automatic depth cutoffs *(5, 70, 136, 137, 219,* and *534*). We then renamed all contigs and haplotigs in the FALCON‐Unzip naming convention for further processing using scripts in R and python (File S6). Briefly, haplotigs that had associated primary contigs in the dups.bed file were renamed to match their respective primary contigs. Those that did not have matches (i.e., contigs with low coverage in round 1 of purging etc.) were aligned to the primary assembly using nucmer (Marçais et al., 2018) and BLAST. The primary contig with the longest set of alignments was selected as the associated primary contig.

#### Haplotype phasing

The resulting pseudo‐haplotype primary contigs and haplotigs alongside the Hi‐C data were passed to FALCON‐Phase for phase switch correction, creating one complete set of contigs for each phase (Kronenberg et al., 2018). However, due to the large number of SVs between the TME7 haplotypes, we modified the *coords2hp.py* script in FALCON‐Phase to always include the entire length of the haplotig in placement (File S6). This reduced the length of haplotig sequence discarded by FALCON‐Phase during phasing. We output the results in both “pseudohap” and “unzip” formats.

#### Scaffolding

The Proximo Hi‐C genome scaffolding platform from Phase Genomics (Seattle, WA, USA) was used to create chromosome‐scale scaffolds from the FALCON‐Phase Phase0 assembly, following the same single‐phase scaffolding procedure described in Bickhart et al. (2017). As in the LACHESIS method (Burton et al., 2013), this process computes a contact frequency matrix from the aligned Hi‐C read pairs, normalized by the number of *Sau*3AI restriction sites (GATC) on each contig, and constructs scaffolds in such a way as to optimize expected contact frequency and other statistical patterns in Hi‐C data. Juicebox (Durand et al., 2016; Rao et al., 2014) was then used to correct scaffolding errors. The Hi‐C contact map was created by separately aligning the Hi‐C read pairs to the scaffolded genome then generating a Hi‐C contact matrix using the command line version of HiCExplorer (Wolff et al., 2020). A 10‐kb matrix was first created, then bins were merged to get a 500‐kb resolution for ease of plotting. Bin interaction data was then exported to table separated format (tsv) and imported to R for plotting.

### Assembly quality assessment

#### Linkage map alignment

To confirm the order and contiguity of the assembly further we aligned the 22k marker composite linkage map (ICGMC, 2015) from cassava base (cassavabase.org). In this map, each SNP marker is aligned to the cassava v4.1 draft genome assembly and a scaffold and physical position is reported alongside the genetic position. Using the *marker_seqs.py* python script (File S6) we extracted sequences from 100 nt on both sides of each SNP in the v4.1 assembly. If the SNP marker was closer than 100 nt from the end of a scaffold, then the sequence with the maximum length possible around that SNP was extracted. These approximately 200 nt sequence tags were then aligned via BLAST to each phase of the current assembly. The numbers of uniquely mapping markers with alignment length >150 nt and >95% identity were used to assess levels of sequence duplication.

#### K‐mer based evaluation

Merqury (Rhie et al., 2020) and the built‐in Meryl implementation were used to enumerate the *k*‐mer distribution in the Illumina PE reads and compare it to the diploid and haploid assemblies. Using the provided script in Merqury, a *k* = 21 was selected to best represent a genome size of approximately 700 Mb. Copy number spectra and assembly spectra were plotted using the hist files provided and ggplot2. When *k*‐mer distributions were used to estimate genome sequence length (i.e., to measure missing sequence space), the sum of counts of *k*‐mers under the respective distribution was divided by the mean *k*‐mer multiplicity of the distribution:

$$\begin{array}{l} \left(sum\left(kmercount*kmermultiplicity\right)\right)\\ /mean\left(kmermultiplicity\right) \end{array}$$

#### Assessment of genic and repetitive sequence space

The completeness and duplication of the genic regions in the assembly was performed by using BUSCO v4.1.2 (Simão et al., 2015) benchmark software (http://busco.ezlab.org/) and the “eudicotyledons_odb10” ortholog dataset with default settings.

To evaluate the contiguity of the repetitive sequence assembly, LAI was evaluated using LAI beta3.2 (Ou et al., 2018) with input files generated by EDTA. The initial LAI estimation was done using the “*‐q*” parameter, then average LTR identity and total LTR content were obtained and further provided to the standardization of LAI, with parameters “*‐iden 95.63 ‐totLTR 53*.” Regional LAI was calculated in 3 Mb windows with 300 kb overlapping steps.

### Haplotype‐specific annotation

#### TE annotation and repeat masking

TEs of each assembly were independently annotated using EDTA v1.9.7 (Ou et al., 2019) with parameters “*‐‐sensitive 1 ‐‐anno 1 ‐t 18*” and “*‐‐cds*” providing the coding sequences of the *M*. *esculenta* v6.1 assembly. Library sequences from the *de novo* TE library generated by EDTA were filtered and those present more than three full‐length copies in the respective haploid assembly were retained. The remaining sequences from the two TE libraries were combined using the “*make_panTElib.pl*” script in the EDTA package, generating a high‐quality TE library. The final TE library was then used to annotate the two haploid genomes using RepeatMasker v4.1.1 (www.repeatmasker.org) with parameters “*‐q ‐no_is ‐norna ‐nolow ‐div 40 ‐cutoff 225*” that allow for up to 40% of sequence divergence. This step helped to annotate fragmented TEs. To annotate consistently the intact TEs in the two haploid genomes, the final TE library and the final homology‐based TE annotation were provided to EDTA with parameters “*‐‐evaluate 1 ‐‐anno 1 ‐t 18 ‐‐step final*.” In depth commands for TE annotation and LAI calculation are supplied in File S6.

#### Gene annotation

Transcriptome data of 11 tissue types (Wilson et al., 2017) was used to generate transcript evidence for annotation. Reads were trimmed with Trimmomatic (Bolger et al., 2014) and aligned to the soft masked diploid reference (Phase0 scaffolds + Phase1 pseudohaplotype contigs concatenated) using Hisat2 v2.1.0 (Kim et al., 2019). Stringtie v1.3.5 was used to assemble transcripts from each alignment file and all files were merged with “stringtie merge” (Pertea et al., 2015). A fasta containing coding sequence for all transcripts was produced using gffread tool from the cufflinks (Trapnell et al., 2010) package. These transcripts, together with AM560‐2 v6.1 coding sequences and protein sequence from Araport11 (Cheng et al., 2017), were used for a first round of MAKER v2.31.8 (Cantarel et al., 2008) gene annotation. Gene prediction was further performed by training SNAP (library 2013‐02‐16) (Korf, 2004) and AUGUSTUS v3.3 (Stanke and Morgenstern, 2005) as suggested in Bowman et al. (2017) and the output of the first round of MAKER annotation. After gene prediction the genes in the gff file were renamed and the file was split to produce one gff for each phase.

#### Gene synteny and tandem duplication analyses

Comparison of gene synteny between the TME7 Phase0 assembly and the AM560‐2 ref6 assembly was performed with the Python MCScanX pipeline v1.1.12 (Tang et al., 2008; Wang et al., 2012). Briefly, annotation gff files were converted to bed format keeping one isoform per gene using “*jcvi.formats.gff ‐‐primary_only*”. A pairwise synteny search was performed and the high‐quality synteny block (anchors) was used in syntenic depth comparisons and plotting of karyotypes and dot plots. Gene synteny depths between the Phase0 and Phase1 annotations were calculated in the same way. For each phase, tandem gene duplication arrays were identified similar to the study Van Buren et al. (2018). Briefly, arrays were detected by MCScan (Tang et al., 2008) using results from an all‐vs‐all BLASTP (e‐value < 1e‐10) and a maximum gene distance of 10. Arrays were extracted from the “*.tandem*” file and enumerated using a short script in R (File S7).

### SV and polymorphisms

SVs between TME7 and the AM560‐2 reference genome were identified by aligning the Phase0 contigs vs. the reference genome. The authors of the assemblytics (Nattestad and Schatz, 2016) software recommend analysis using contigs and not scaffolds, to minimize bias from different gap sizes in the assembly. Thus, initially the reference assembly was split at gaps of >10 Ns using the python script *split_scaffolds.py* (File S6). After alignment with nucmer (Marçais et al., 2018) with settings: “*‐‐maxmatch ‐l 100 ‐c 500*” the delta file was gziped and uploaded to the Assemblytics web interface (www.assemblytics.com) for analysis using a maximum variant size of 10 000 or 50 000 bp. The results were exported as a bed file and imported into R for plotting. Dot plots of the alignments were produced using scripts modified from https://jmonlong.github.io/Hippocamplus/2017/09/19/mummerplots‐with‐ggplot2/ (File S7).

The locations of the five largest deletions identified were then examined for evidence of SV using short‐read mapping. Deduplicated Illumina reads from TME7 were aligned to the AM560‐2 v6.1 reference using bwa mem (Li and Durbin, 2009). The sorted bam file was then loaded into samplot (Belyeu et al., 2021) to plot the read coverage and identification of discordant mapping. SNPs and INDELs were identified by using dnadiff and show‐snps programs in the MUMmer4 package (Marçais et al., 2018).

SV between the phases was then assessed by aligning Phase1 unzip contigs vs. Phase0 scaffolds (split at >10 Ns) and using Assemblytics as above. Haplotype divergence was calculated by aligning the FALCON‐Phase “Unzip”‐emit‐style haplotigs to the primary, Phase0, assembly using nucmer with these settings: “*‐‐maxmatch ‐l 100 ‐c 500*”. Alignments were filtered with delta‐filter ‐g and coordinates were output using show‐coords. Finally, divergence from the primary assembly was calculated using scripts from https://github.com/skingan/FC_Unzip_HaplotypeDivergence. SNPs and INDELs between the phases were identified as above.

### ASE

The above genomic read alignment bam files for TME7 vs. the Phase0 assembly were then deduplicated with Picard tools and SNPs were called using GATK v4.1.4.1 (Van der Auwera et al., 2013) with minimal quality filtering (*QD < 2.0, FS > 60.0, MQ < 40.0, MQRankSum < −12.5, ReadPosRankSum < −8.0*). We then aligned all RNA‐seq data from Wilson et al. (2017), to the TME7 Phase0 assembly using STAR v2.7.8 (Dobin et al., 2013), and the bams and SNP VCF file were then imported to phASER (Castel et al., 2016) to phase the variants accurately within each gene model. To decrease running time we ran PhASER on each bam separately and included only genes on the first 18 chromosomes. PhASER settings were “*‐‐paired_end 1 ‐‐mapq 255 ‐‐baseq 10*”. The haplotypic read counts from all samples were then concatenated, and subsequently the per gene read counts were then exported using the “phASER Gene AE” tool and read into R for statistical analysis.

We used the suggested DESeq2 pipeline for ASE (http://rstudio‐pubs‐static.s3.amazonaws.com/275642_e9d578fe1f7a404aad0553f52236c0a4.html) for analysis. Using this approach, statistical contrasts were evaluated for each tissue type between the counts of REF and ALT alleles, similarly, to how one might identify treatment effects in a regular RNA‐seq analysis. Significance *P*‐values were adjust using the Benjamini–Hochberg method (Benjamini and Hochberg, 1995) and genes were marked as ASE if the adjusted *P*‐value was <0.05. PCAs were performed once on the variance‐stabilized read counts and once on the log2(ALT count/REF count) values calculated by PhASER. A constant of 0.01 was added to the counts to limit log2 of zero. We further categorized ASE genes as having “Complete ASE” or “Partial ASE” if log2 allele ratios were ≥5, respectively. Distances to nearest non‐overlapping INDEL were measured using bedtools *closest* command using the ‐D a ‐io and ‐id or ‐iu settings for upstream and downstream INDELs, respectively (Quinlan and Hall, 2010). The distributions of distances of genes in different ASE categories were compared using the Kolmogorov–Smirnov test in R.

### GO annotation and enrichment analyses

A GO term database was established as in Mansfeld et al. (2017); all TME7 proteins were compared with Araport11 (Cheng et al., 2017) by BLASTP and Protein families were identified using InterProScan5 (Jones et al., 2014). GO terms were extracted from these results, and merged and deduplicated to make the final database. The GO term enrichment analysis was performed using TopGO (Alexa et al., 2006) using all expressed genes as a background set and a minimum node size of 100. The GO enrichment of genes in tandem arrays was performed as above but using all annotated genes as a background set.

### SHINY app update

Reads from the RNA‐seq dataset for 11 tissue types were aligned to the TME7 Phase0 assembly using HISAT2 (Kim et al., 2019) and abundance was quantified with Stringtie (Pertea et al., 2015). Read counts were transformed into robust‐FPKMs using DESeq2 (Love et al., 2014). Finally, the annotation was matched to the transcript IDs and formatted to be read within the Shiny framework.

### Scripts and figures

All scripts described above are supplied in File S6. All R scripts for producing figures and summary results are supplied in File S7.

## AUTHOR CONTRIBUTIONS

BNM and RB conceived and designed the assembly strategy with contribution from NF. TPM generated the initial long read data. AB, MW, and BNM performed the assembly steps. AB and SO annotated the gene and TE space. Genome‐wide analyses and assembly assessments were performed by BNM, AB, SO, and SP. ASE analysis was performed by BNM. AB and JCB updated the cassava expression atlas application. BNM and RB wrote the manuscript with comments from all authors.

## CONFLICT OF INTERESTS

The authors declare that they have no competing interests.

## Supporting information


**Table S1.** Statistics of other assembly attempts.
**Table S2.** Gene Ontology enrichment of tandem duplicated genes.
**Table S3.** Gene Ontology enrichment of genes with allele‐specific expression.
**Figure S1.** Full GenomeScope *k*‐mer profile.
**Figure S2.** PacBio read length distribution.
**Figure S3.** Diploid *k*‐mer count spectra for assembly stages.
**Figure S4.** Reduction of heterozygous sequences by FALCON‐Phase.
**Figure S5.** Linkage map hit duplication rate.
**Figure S6.** Distribution of long terminal repeats.
**Figure S7.** Distributions of transposable elements.
**Figure S8.** Annotation statistics.
**Figure S9.** Gene syntenic depth analyses.
**Figure S10.** Sequence alignment to AM560‐2 reference genome.

**File S1.** Gene comparison between the two phase’s annotation (Anchor file).

**File S2.** Structural variants between Phase0 and the AM560‐2 reference genome.

**File S3.** Structural variants between the two phases.

**File S4.** Allele specific analysis and relation to structural variants in 11 tissues.

**File S5.** Configuration file for FALCON suite.

**File S6.** Additional scripts.

**File S7.** Scripts for figures and assessment.

## Data Availability

Both haplotype genome assemblies and annotations are stored in the Zenodo data repository https://doi.org/10.5281/zenodo.5534689. All short and long reads in assembly have been uploaded under the SRA accession PRJNA765105. Custom scripts used for assembly and analysis are available in Files S5–7 and at https://github.com/bmansfeld/tme7_cassava_genome_figures/.
