# Supplementary material for: Large structural variations in the haplotype‐resolved African cassava genome
Source: Plant J. 2021 Nov 10;108(6):1830–48. doi: 10.1111/tpj.15543 (PMC9299708; doi:10.1111/tpj.15543)
Supplement: Supplementary file 1 — Table S1. Statistics of other assembly attempts. Table S2. Gene Ontology enrichment of tandem duplicated genes. Table S3. Gene Ontology enrichment of genes with allele‐specific expression. Figure S1. Full GenomeScope k‐mer profile. Figure S2. PacBio read length distribution. Figure S3. Diploid k‐mer count spectra for assembly stages. Figure S4. Reduction of heterozygous sequences by FALCON‐Phase. Figure S5. Linkage map hit duplication rate. Figure S6. Distribution of long terminal repeats. Figure S7. Distributions of transposable elements. Figure S8. Annotation statistics. Figure S9. Gene syntenic depth analyses. Figure S10. Sequence alignment to AM560‐2 reference genome. [file TPJ-108-1830-s001.docx]

**Supplementary Material: Large structural variations in the haplotype-resolved African cassava genome.**

*Author order TBD:*

^1^Ben N. Mansfeld: 0000-0001-6118-6409

^1^Adam Boyher: 0000-0001-9681-1817

^1^Jeffrey C. Berry: 0000-0002-8064-9787

^1^Mark Wilson:

^2^Shujun Ou: 0000-0001-5938-7180

^1^Seth Polydore: 0000-0002-7779-7367

^3^Todd P. Michael: 0000-0001-6272-2875

^1^Noah Fahlgren: 0000-0002-5597-4537

^1^Rebecca S. Bart: 0000-0003-1378-3481

**Supplementary Table 1. Other assembly attempts and the resulting assembly statistics.**

|  | Canu v1.7  (Default settings) –  genomeSize=750m | FALCON and FALCON-Unzip falcon-kit v0.4.0* | |
| --- | --- | --- | --- |
|  |  | Primary | Haplotigs |
| Total length assembled (bp) | 1,105,799,943 | 900,836,955 | 130,487,209 |
| Number of contigs | 20,055 | 5,873 | 2,460 |
| Contig N50 | 91,652 | 234,455 | 76,564 |
|  |  |  |  |

***Alternate Falcon suite settings**:

*[General]*

*job_type = local*

*# list of files of the initial bas.h5 files*

*input_fofn = input.fofn*

*#input_fofn = preads.fofn*

*input_type = raw*

*#input_type = preads*

*# The length cutoff used for seed reads used for initial mapping*

*# set at 2000 because the average is 6800*

*length_cutoff = 5000*

*# The length cutoff used for seed reads use for pre-assembly*

*# set at 2000 because the average is 6800*

*length_cutoff_pr = 7000*

*sge_option_da = -pe smp 2 -q all.q*

*sge_option_la = -pe smp 2 -q all.q*

*sge_option_pda = -pe smp 2 -q all.q*

*sge_option_pla = -pe smp 2 -q all.q*

*sge_option_fc = -pe smp 24 -q all.q*

*sge_option_cns = -pe smp 8 -q all.q*

*pa_concurrent_jobs = 20*

*ovlp_concurrent_jobs = 20*

*# set -dal32 to have less jobs*

*# min read length can be set with the -l option*

*pa_HPCdaligner_option = -v -dal8 -t16 -e.70 -l1000 -s1000 -M10*

*ovlp_HPCdaligner_option = -v -dal8 -t32 -h60 -e.96 -l1000 -s1000 -M10*

*# set -s400 to define block sizes of 400Mb for a smaller number of jobs because this will be running on a machine that can handle jobs that run for longer*

*pa_DBsplit_option = -x500 -s350*

*ovlp_DBsplit_option = -x500 -s350*

*falcon_sense_option = --output_multi --min_idt 0.70 --min_cov 4 --local_match_count_threshold 2 --max_n_read 200 --n_core 6*

*overlap_filtering_setting = --max_diff 70 --max_cov 90 --min_cov 4 --bestn 10*

**Falcon unzip settings**

*[General]*

*job_type = local*

*[Unzip]*

*smrt_bin= /home/mawilson/local/smrtsuite/smrtcmds/bin/*

*sge_phasing = -pe smp 12 -q bigmem*

*sge_unzip= -pe smp 24 -q bigmem*

*sge_quiver= -pe smp 24 -q bigmem*

*unzip_concurrent_jobs = 15*

*quiver_concurrent_jobs = 15*

**Supplementary Table 2. Tandem gene duplication gene ontology enrichment analysis.**

| **ID** | **Term** | **Annotated** | **Significant** | **Expected** | **P-value**  ***Fisher***  ***weight01*** |
| --- | --- | --- | --- | --- | --- |
| GO:0055114 | oxidation-reduction process | 919 | 119 | 51.82 | 1.70E-18 |
| GO:0007154 | cell communication | 1110 | 77 | 62.59 | 3.80E-10 |
| GO:0009856 | pollination | 182 | 23 | 10.26 | 9.80E-09 |
| GO:1901617 | organic hydroxy compound biosynthetic pr... | 142 | 26 | 8.01 | 8.40E-08 |
| GO:1901615 | organic hydroxy compound metabolic proce... | 261 | 47 | 14.72 | 1.80E-06 |
| GO:0008610 | lipid biosynthetic process | 406 | 44 | 22.89 | 3.60E-06 |
| GO:0048878 | chemical homeostasis | 274 | 26 | 15.45 | 3.60E-06 |
| GO:0006952 | defense response | 661 | 55 | 37.27 | 1.60E-05 |
| GO:0005975 | carbohydrate metabolic process | 602 | 55 | 33.95 | 1.10E-04 |
| GO:0042221 | response to chemical | 1755 | 117 | 98.96 | 2.50E-04 |
| GO:0006518 | peptide metabolic process | 518 | 22 | 29.21 | 4.50E-04 |
| GO:0032259 | methylation | 174 | 12 | 9.81 | 5.00E-04 |
| GO:0007275 | multicellular organism development | 1694 | 85 | 95.52 | 5.00E-04 |
| GO:0051707 | response to other organism | 676 | 60 | 38.12 | 6.20E-04 |
| GO:0032502 | developmental process | 2057 | 108 | 115.99 | 8.40E-04 |
| GO:0010817 | regulation of hormone levels | 153 | 19 | 8.63 | 9.80E-04 |
| GO:0007165 | signal transduction | 937 | 51 | 52.84 | 0.00112 |
| GO:1901360 | organic cyclic compound metabolic proces... | 3098 | 139 | 174.69 | 0.00145 |
| GO:0009057 | macromolecule catabolic process | 589 | 27 | 33.21 | 0.00203 |
| GO:1901362 | organic cyclic compound biosynthetic pro... | 1802 | 88 | 101.61 | 0.00405 |
| GO:0009056 | catabolic process | 1074 | 66 | 60.56 | 0.00424 |
| GO:0006073 | cellular glucan metabolic process | 134 | 15 | 7.56 | 0.00847 |
| GO:0044248 | cellular catabolic process | 864 | 46 | 48.72 | 0.01134 |
| GO:0006633 | fatty acid biosynthetic process | 114 | 13 | 6.43 | 0.01183 |
| GO:0009409 | response to cold | 288 | 26 | 16.24 | 0.01202 |
| GO:0019748 | secondary metabolic process | 181 | 18 | 10.21 | 0.0137 |
| GO:0009620 | response to fungus | 184 | 19 | 10.38 | 0.01395 |
| GO:0009832 | plant-type cell wall biogenesis | 104 | 12 | 5.86 | 0.01397 |
| GO:0000910 | cytokinesis | 108 | 12 | 6.09 | 0.0184 |
| GO:0006468 | protein phosphorylation | 879 | 59 | 49.57 | 0.01971 |
| GO:0014070 | response to organic cyclic compound | 176 | 17 | 9.92 | 0.0211 |
| GO:0006950 | response to stress | 2095 | 137 | 118.13 | 0.02232 |
| GO:0033692 | cellular polysaccharide biosynthetic pro... | 111 | 12 | 6.26 | 0.02236 |
| GO:0048856 | anatomical structure development | 1923 | 95 | 108.44 | 0.02246 |
| GO:1901565 | organonitrogen compound catabolic proces... | 585 | 30 | 32.99 | 0.02277 |
| GO:1903047 | mitotic cell cycle process | 167 | 16 | 9.42 | 0.02648 |
| GO:0006721 | terpenoid metabolic process | 103 | 11 | 5.81 | 0.03031 |
| GO:0016052 | carbohydrate catabolic process | 158 | 15 | 8.91 | 0.03323 |
| GO:0005976 | polysaccharide metabolic process | 244 | 26 | 13.76 | 0.0339 |
| GO:0009642 | response to light intensity | 106 | 11 | 5.98 | 0.03637 |
| GO:0006508 | proteolysis | 627 | 33 | 35.36 | 0.03652 |
| GO:0009414 | response to water deprivation | 246 | 21 | 13.87 | 0.03808 |
| GO:0042742 | defense response to bacterium | 276 | 23 | 15.56 | 0.03926 |
| GO:0042325 | regulation of phosphorylation | 130 | 4 | 7.33 | 0.04147 |
| GO:0010243 | response to organonitrogen compound | 163 | 15 | 9.19 | 0.042 |

**Supplementary Table 3. Gene ontology analysis of genes with allele specific expression in at least one tissue type.**

| **ID** | **Term** | **Annotated** | **Significant** | **Expected** | **P-value**  ***Fisher***  ***weight01*** |
| --- | --- | --- | --- | --- | --- |
| GO:1901565 | organonitrogen compound catabolic proces... | 585 | 315 | 274.69 | 4.80E-05 |
| GO:1901566 | organonitrogen compound biosynthetic pro... | 1096 | 591 | 514.64 | 1.30E-04 |
| GO:1901607 | alpha-amino acid biosynthetic process | 131 | 82 | 61.51 | 2.10E-04 |
| GO:0044282 | small molecule catabolic process | 197 | 120 | 92.5 | 2.50E-04 |
| GO:0044270 | cellular nitrogen compound catabolic pro... | 148 | 81 | 69.49 | 2.90E-04 |
| GO:0045935 | positive regulation of nucleobase-contai... | 314 | 124 | 147.44 | 8.90E-04 |
| GO:0010604 | positive regulation of macromolecule met... | 458 | 190 | 215.06 | 9.00E-04 |
| GO:0046700 | heterocycle catabolic process | 145 | 78 | 68.09 | 0.00113 |
| GO:0055086 | nucleobase-containing small molecule met... | 301 | 175 | 141.34 | 0.00117 |
| GO:0006091 | generation of precursor metabolites and ... | 267 | 150 | 125.37 | 0.0014 |
| GO:0006518 | peptide metabolic process | 518 | 279 | 243.23 | 0.00212 |
| GO:1901362 | organic cyclic compound biosynthetic pro... | 1802 | 764 | 846.15 | 0.00243 |
| GO:0018130 | heterocycle biosynthetic process | 1670 | 694 | 784.16 | 0.00305 |
| GO:0010035 | response to inorganic substance | 614 | 318 | 288.31 | 0.00337 |
| GO:0019438 | aromatic compound biosynthetic process | 1725 | 722 | 809.99 | 0.00499 |
| GO:0044271 | cellular nitrogen compound biosynthetic ... | 2118 | 924 | 994.53 | 0.00512 |
| GO:0042742 | defense response to bacterium | 276 | 151 | 129.6 | 0.00543 |
| GO:0044283 | small molecule biosynthetic process | 503 | 266 | 236.19 | 0.00563 |
| GO:1901361 | organic cyclic compound catabolic proces... | 175 | 93 | 82.17 | 0.00563 |
| GO:0055085 | transmembrane transport | 669 | 344 | 314.14 | 0.00575 |
| GO:1901615 | organic hydroxy compound metabolic proce... | 261 | 139 | 122.56 | 0.0059 |
| GO:0010033 | response to organic substance | 1120 | 538 | 525.91 | 0.00606 |
| GO:0019439 | aromatic compound catabolic process | 159 | 84 | 74.66 | 0.00667 |
| GO:0045229 | external encapsulating structure organiz... | 152 | 75 | 71.37 | 0.00772 |
| GO:0072594 | establishment of protein localization to... | 219 | 121 | 102.83 | 0.00795 |
| GO:0006412 | translation | 443 | 233 | 208.01 | 0.00886 |
| GO:0071702 | organic substance transport | 788 | 408 | 370.01 | 0.00966 |
| GO:0055114 | oxidation-reduction process | 919 | 466 | 431.53 | 0.00987 |
| GO:0017038 | protein import | 100 | 59 | 46.96 | 0.01012 |
| GO:0009814 | defense response, incompatible interacti... | 118 | 68 | 55.41 | 0.01255 |
| GO:0009150 | purine ribonucleotide metabolic process | 159 | 89 | 74.66 | 0.0135 |
| GO:0046395 | carboxylic acid catabolic process | 130 | 74 | 61.04 | 0.01393 |
| GO:0016051 | carbohydrate biosynthetic process | 219 | 106 | 102.83 | 0.01645 |
| GO:0006464 | cellular protein modification process | 1938 | 896 | 910.01 | 0.01767 |
| GO:0006605 | protein targeting | 202 | 110 | 94.85 | 0.01875 |
| GO:0015979 | photosynthesis | 151 | 84 | 70.9 | 0.01947 |
| GO:0006796 | phosphate-containing compound metabolic ... | 1545 | 761 | 725.47 | 0.02107 |
| GO:0051247 | positive regulation of protein metabolic... | 123 | 56 | 57.76 | 0.02289 |
| GO:1901605 | alpha-amino acid metabolic process | 223 | 135 | 104.71 | 0.02378 |
| GO:0044281 | small molecule metabolic process | 1183 | 648 | 555.49 | 0.02511 |
| GO:0016310 | phosphorylation | 1015 | 485 | 476.6 | 0.02647 |
| GO:0022603 | regulation of anatomical structure morph... | 102 | 58 | 47.9 | 0.02792 |
| GO:1901575 | organic substance catabolic process | 954 | 501 | 447.96 | 0.02813 |
| GO:0009165 | nucleotide biosynthetic process | 128 | 74 | 60.1 | 0.03053 |
| GO:0032787 | monocarboxylic acid metabolic process | 372 | 183 | 174.68 | 0.03394 |
| GO:0098662 | inorganic cation transmembrane transport | 162 | 88 | 76.07 | 0.03516 |
| GO:0046148 | pigment biosynthetic process | 105 | 59 | 49.3 | 0.03557 |
| GO:0008654 | phospholipid biosynthetic process | 109 | 61 | 51.18 | 0.03633 |
| GO:0044248 | cellular catabolic process | 864 | 457 | 405.7 | 0.03948 |
| GO:0048583 | regulation of response to stimulus | 506 | 245 | 237.6 | 0.03993 |


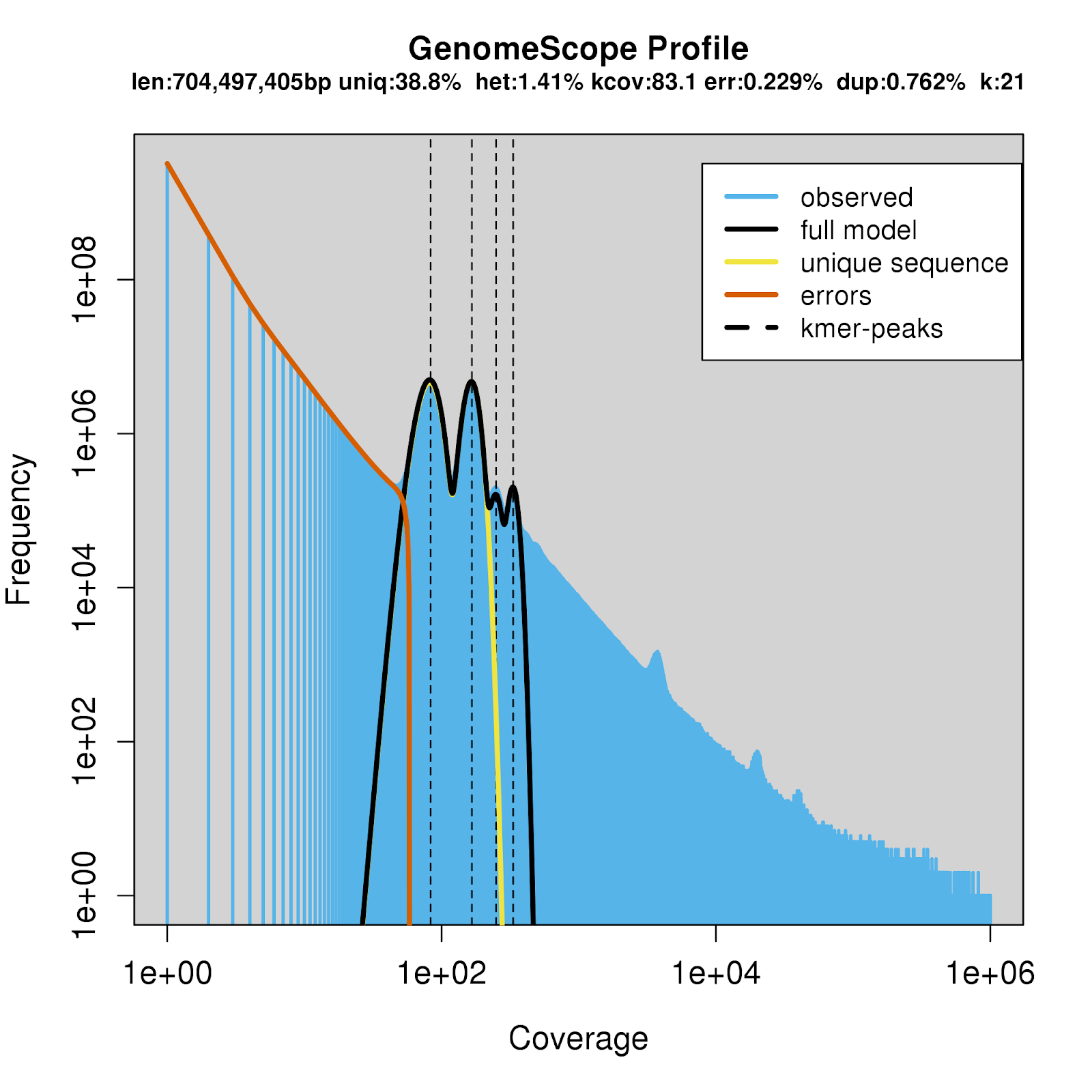
**Supplementary Figure 1. Estimation of genome size, heterozygosity, and repetitiveness using GenomeScope Profile.** The k-mer spectra from Illumina reads were used to estimate the genome parameters. The full k-mer spectra are shown here compared to main text Figure 1. K-mer size was set to 21, and k-mer coverage cutoff was set at 1e6 to include repeat regions in genome size estimates. The haploid genome size was estimated to be 704 Mb consisting of 61% repetitive sequence and a heterozygosity of 1.41%.


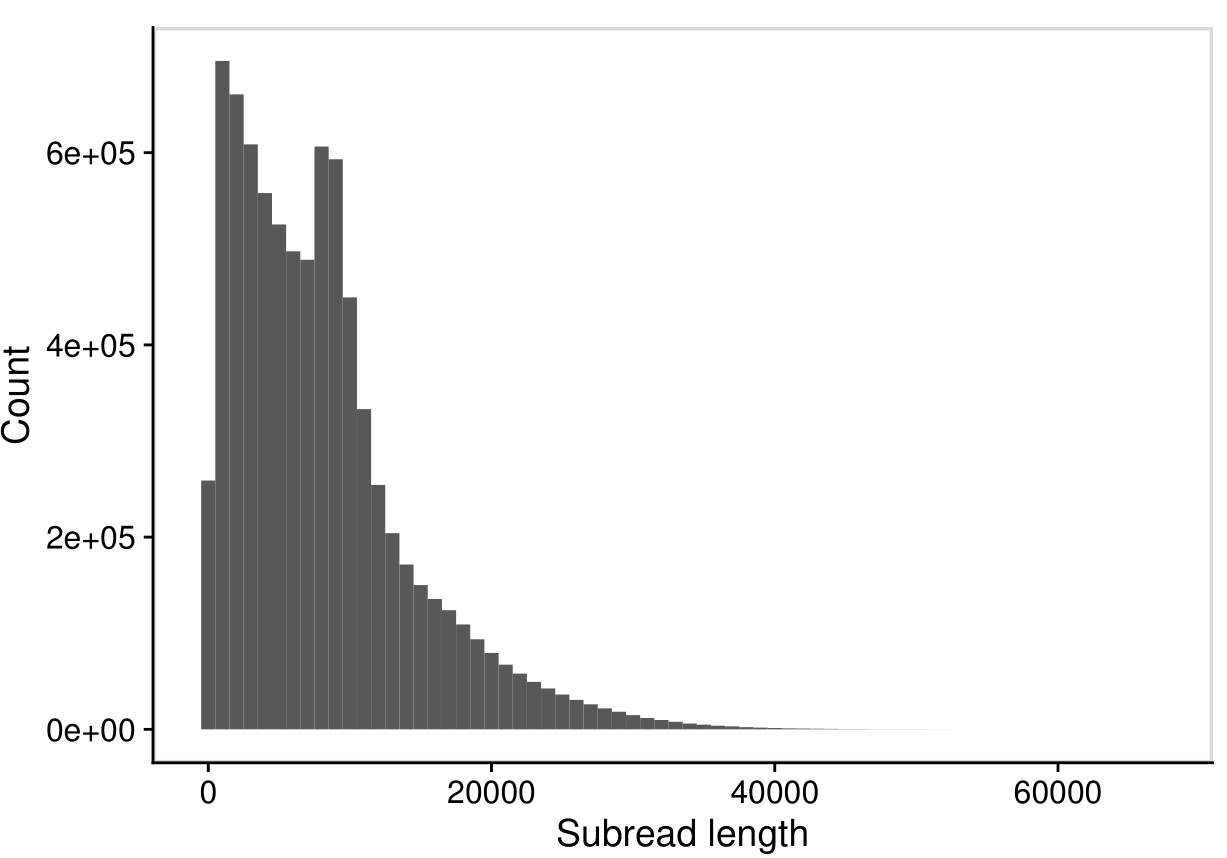


**Supplementary Figure 2. Pacific Bioscience sequencing subread length distribution.** Sequencing was done at two different times. The first round did not include a blue pippin size selection while the second round did; this can be observed as two peaks in the read length distribution.

**
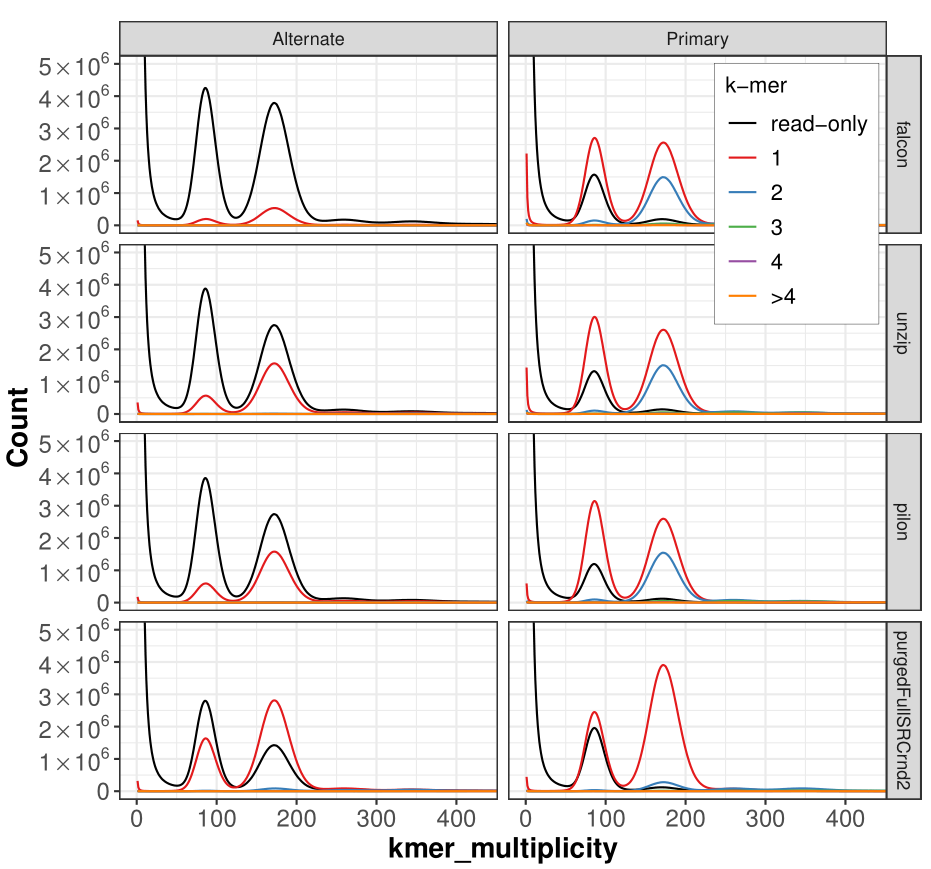
Supplementary Figure 3. Diploid k-mer count spectra for different assembly stages.** Short read k-mer distribution plots are colored by the number of times a k-mer is present in every diploid (primary + alternate) assembly. K-mers denoted in black are missing from the assembly and represent probable short read sequencing errors (k-mer multiplicity <~ 50) or missing assembled sequence (≥~50). Purging the primary assembly (bottom panel) using purge_dups, greatly reduced the duplicated homozygous k-mer levels (blue peak ~175 k-mer multiplicity) in the primary assembly and increased the heterozygous k-mers in the alternate assembly (red peak ~75 k-mer multiplicity). This indicates that the haplotypes are well resolved.


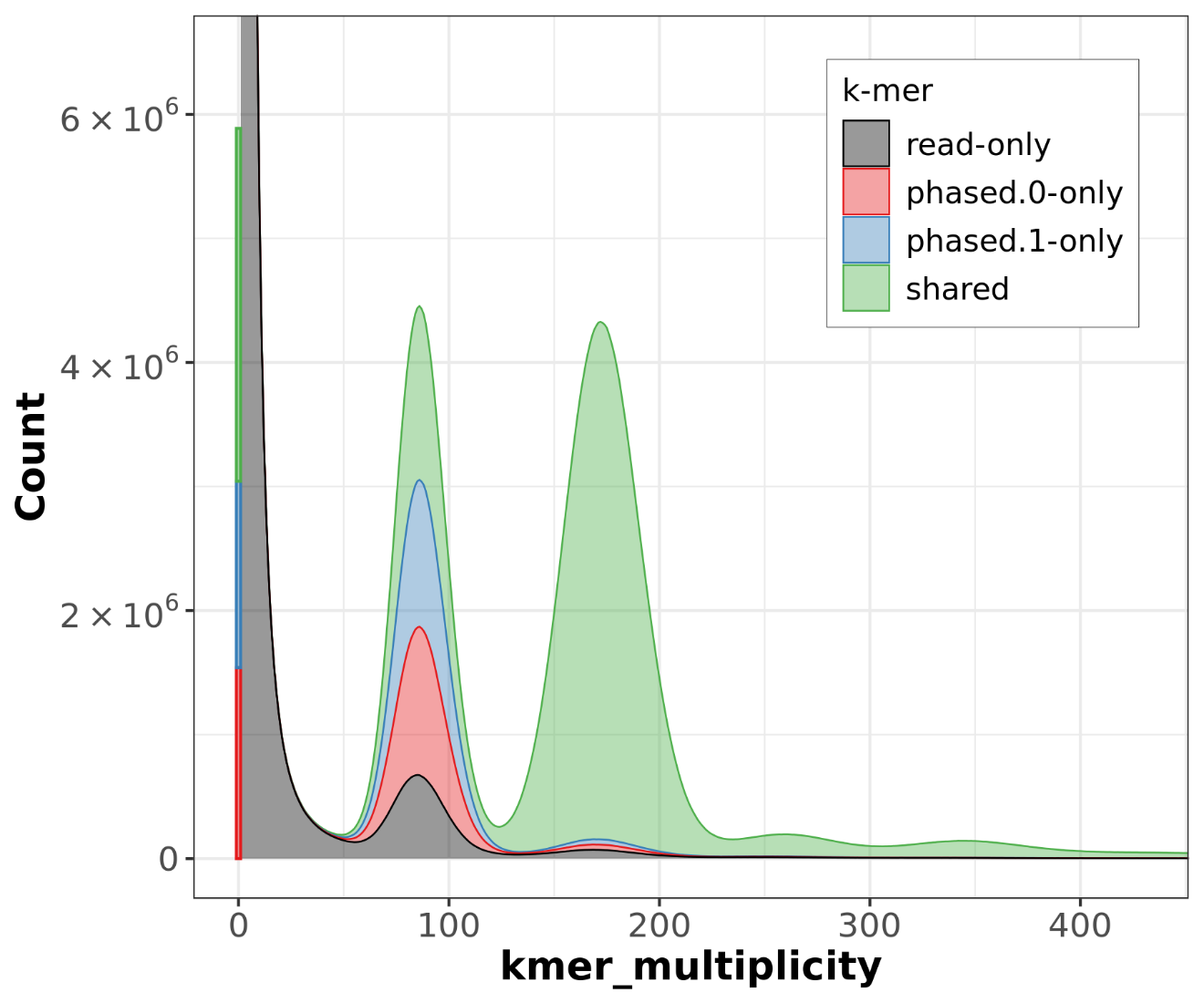
**Supplementary Figure 4. FALCON-Phase causes a reduction in heterozygous sequence with default settings.** With comparison to main text Figure 2C, a stark reduction in phase1-unique sequence (blue) is observed when using the default setting. Furthermore, an increase in “read-only” k-mers which are missing from the assembly is also observed.


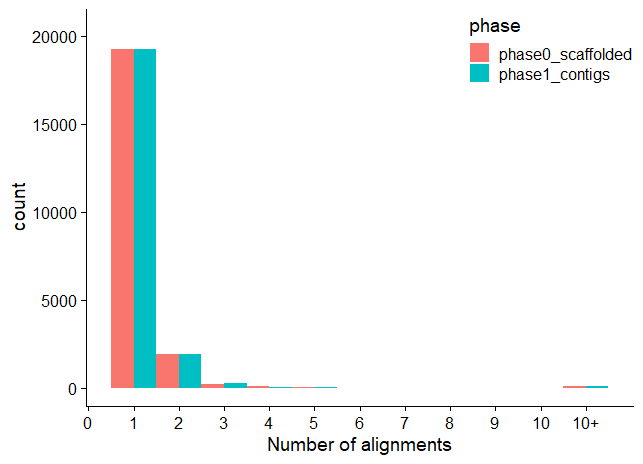


**A**


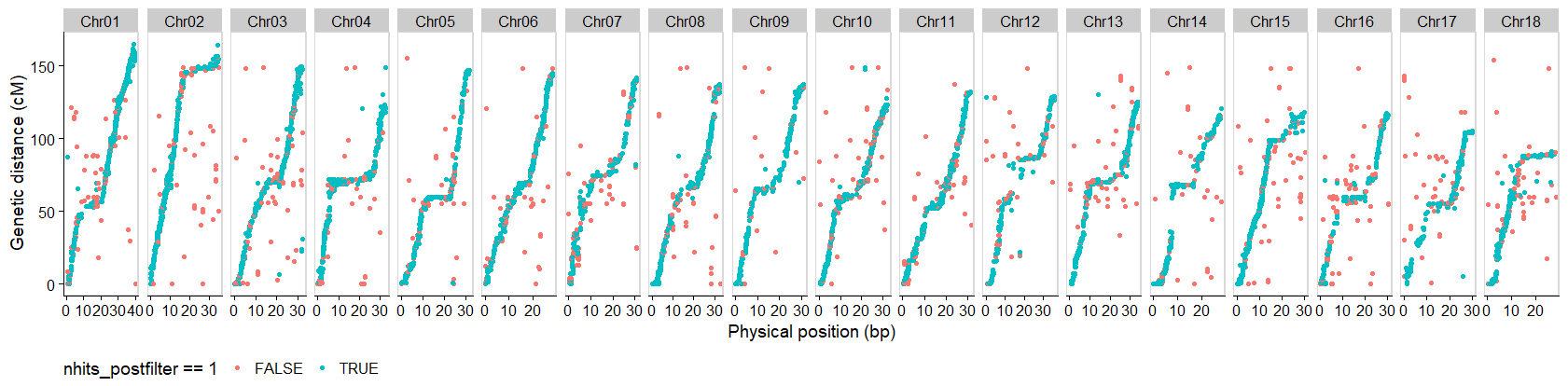


**B**

**Supplementary Figure 5. Linkage map duplication rates are minimal in the TME7 assembly. (A)** The number of filtered alignments for each of the 22K markers from the Cassava Linkage Map project. **(B)** Distribution of duplicated markers in the Phase0 assembly. Markers in blue exist exactly once in the assembly.


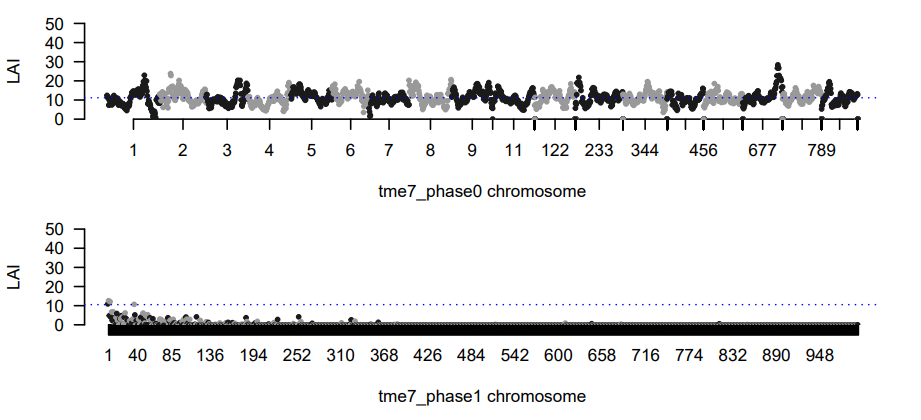


**B**

**A**


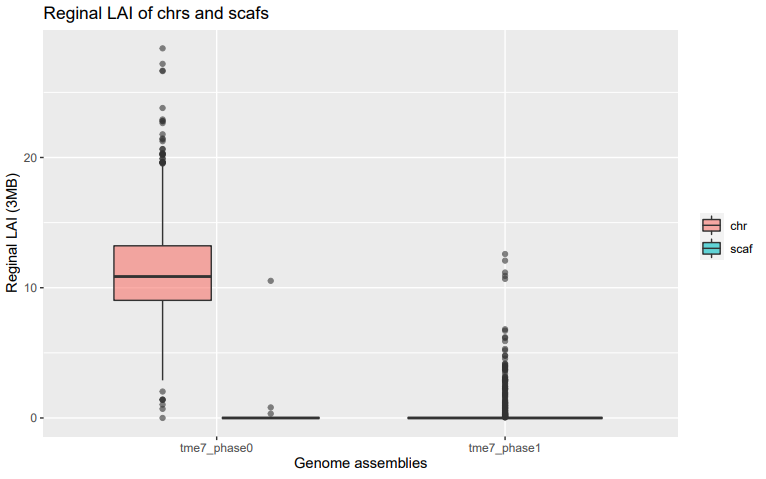


**Supplementary Figure 6. Distributions of Long Terminal Repeats (LTR) assembly index. (A)** LAI (LTR Assembly Index) as a measure of the completeness of the repetitive sequence in the two haplotype assemblies. **(B)** As expected, scaffolding increases the regional LAI.


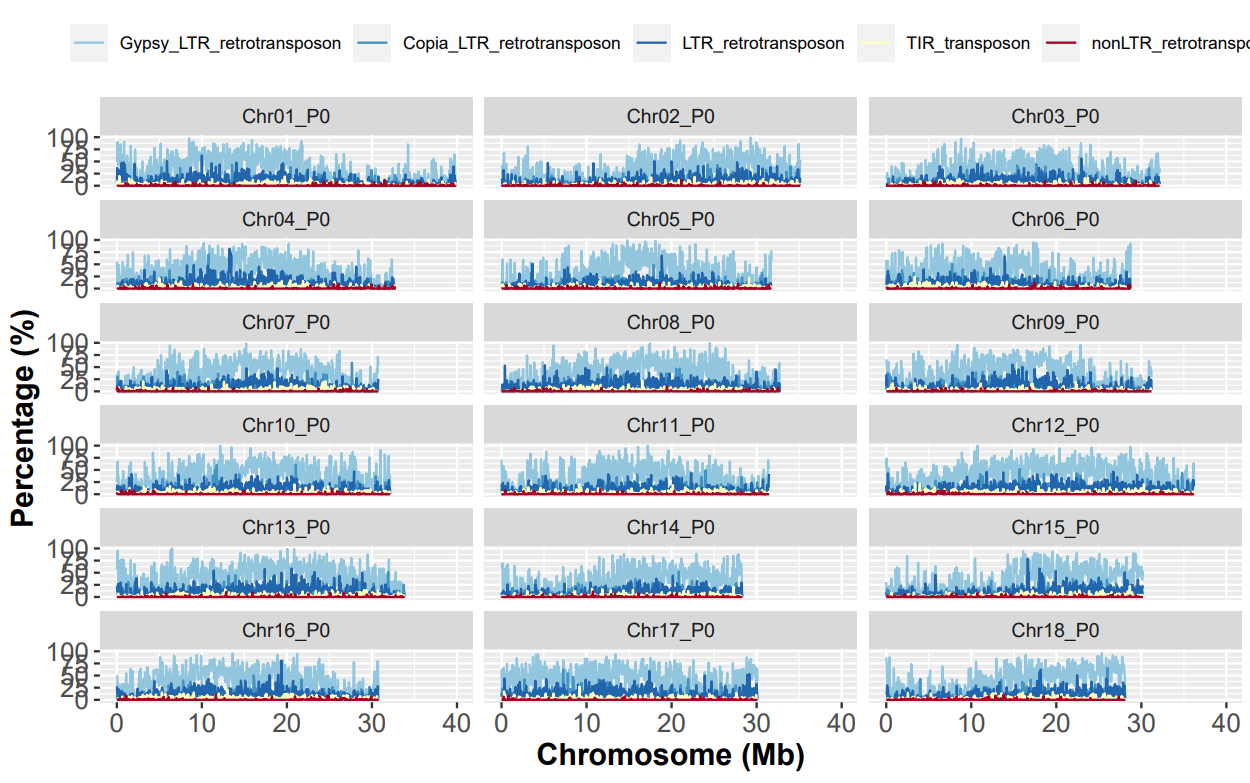
**Supplementary Figure 7. Distributions and types of transposable elements in the TME7 Phase0 assembly.** Transposable element annotation of the TME7 genome showed that ~59% of the genome is comprised of repeats and transposable elements. The repeat landscape is dominated by LTR retrotransposons that contribute about 50.5% of the genome. Terminal inverted repeat (TIR) and Helitron DNA transposons were about 2.43% of the total genome size.


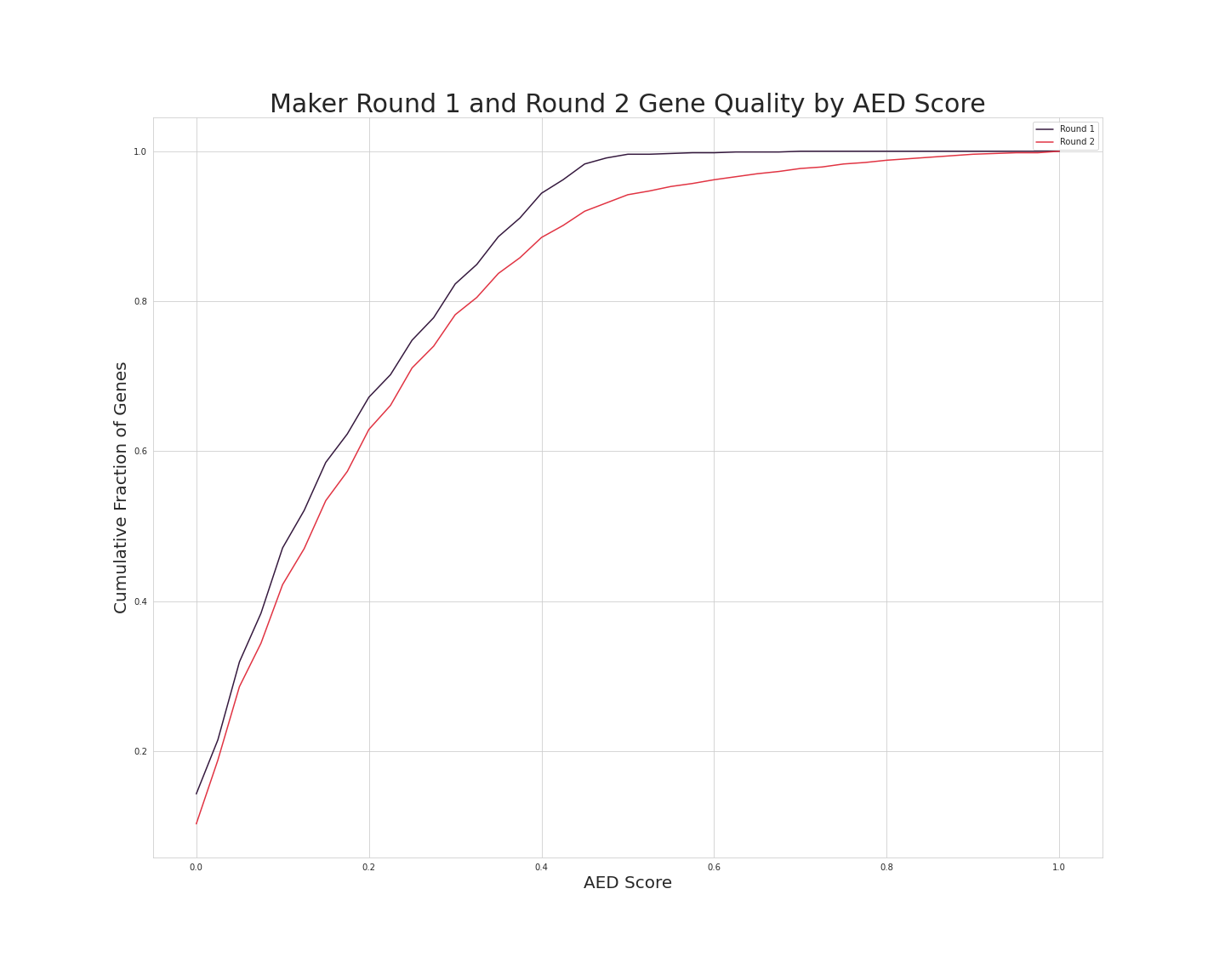


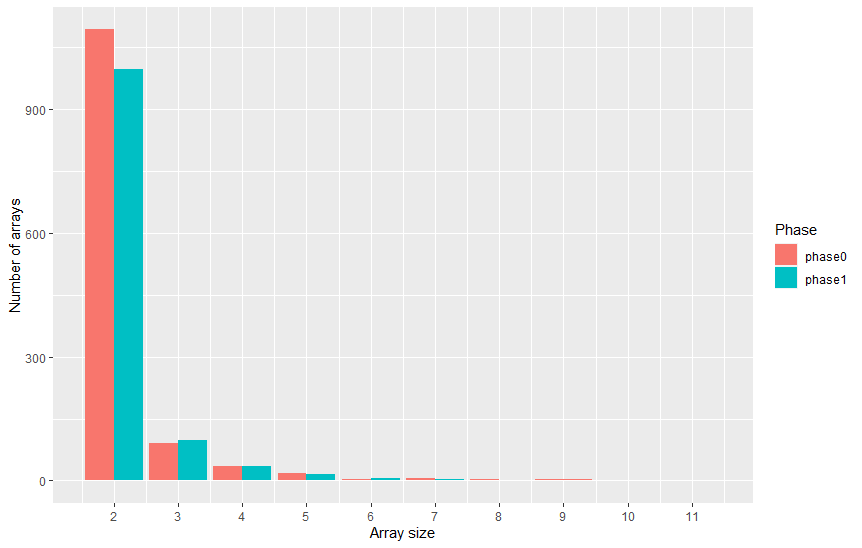


**Supplementary Figure 8. Gene annotation statistics.**

**(A)**The cumulative Annotation Edit Distance (AED) distribution of two rounds of annotation. Round 1 (black) represents an evidence-based gene annotation step with MAKER, while Round 2 (red) includes gene prediction using AUGUSTUS and SNAP. Genes with lower AED have high evidential support. (**B)** A histogram of tandem gene duplication array sizes in the to phases’ annotations.

**
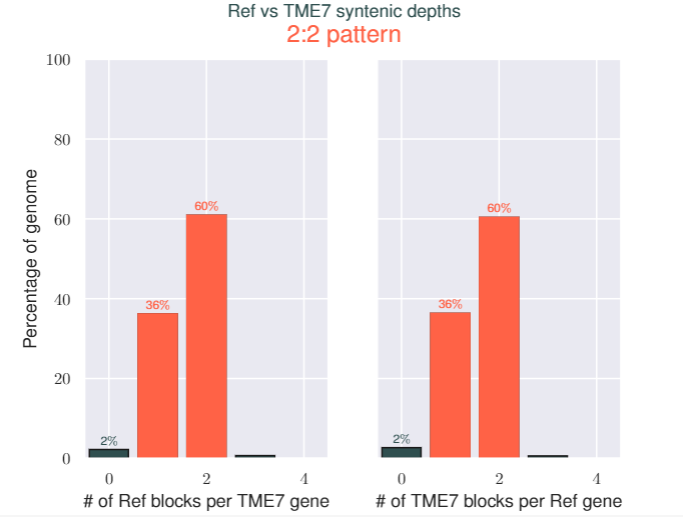
**

**b**

**a**

**
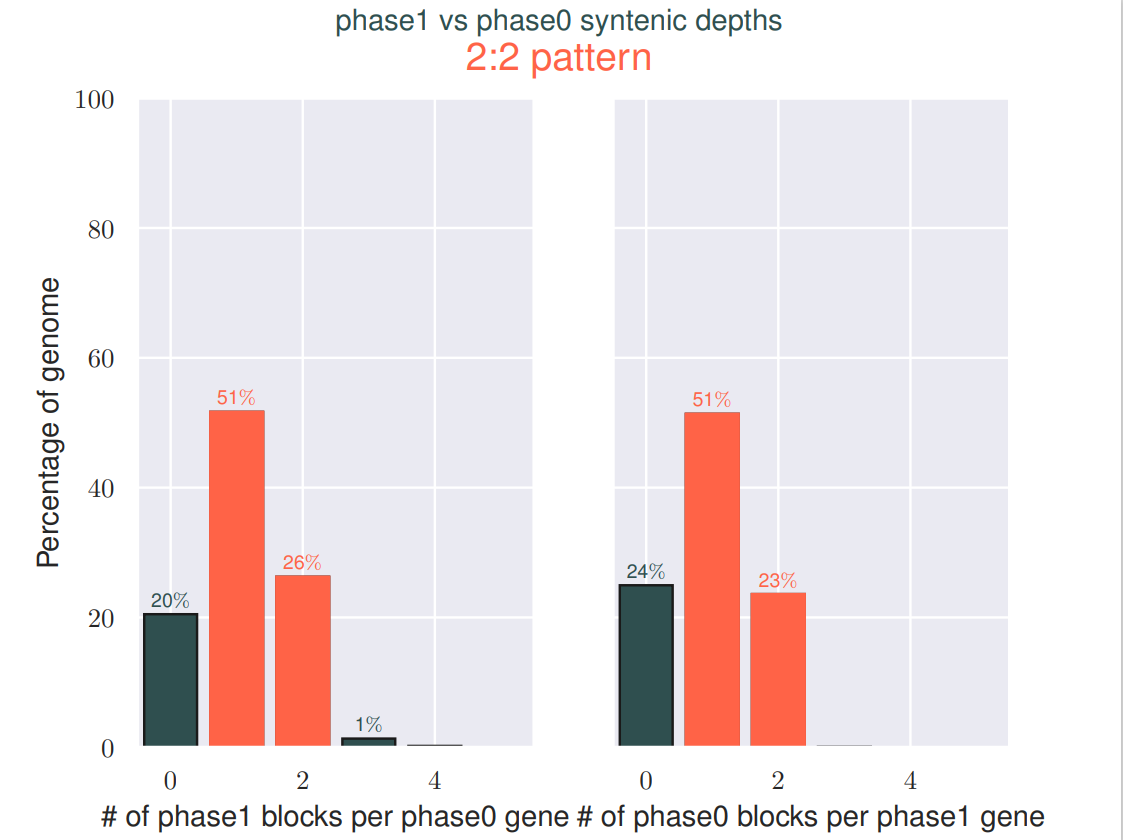
**

**Supplementary Figure 9. Percent of genes and their syntenic depths (A) Comparing blocks between TME7 (Phase0) and AM560-2 reference genome v6.1. (B) Comparison of the TME7 phase1 and Phase0 annoations.** Syntenic blocks of genes were compared using MCScan and the numbers of genes existing in single, and multiple blocks is reported. Most of the genes exist in exactly two syntenic blocks in the reciprocal comparisons due to the paleo-tetraploidization of the cassava genome.

**
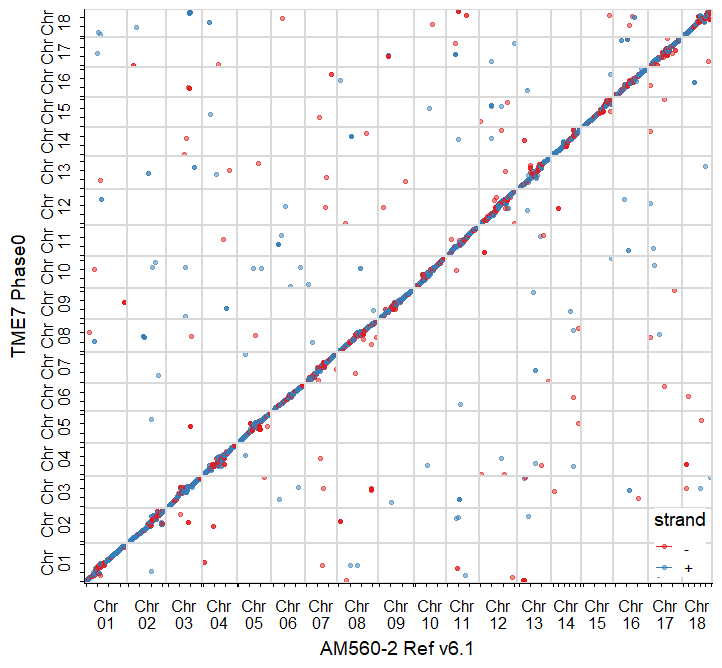
**

**Supplementary Figure 10. Alignment of TME7 Phase0 assembly to the homozygous AM560-2 reference.** Dotplot of the best sequence alignments of the assemblies. Color represents the alignment strand on the reference assembly.


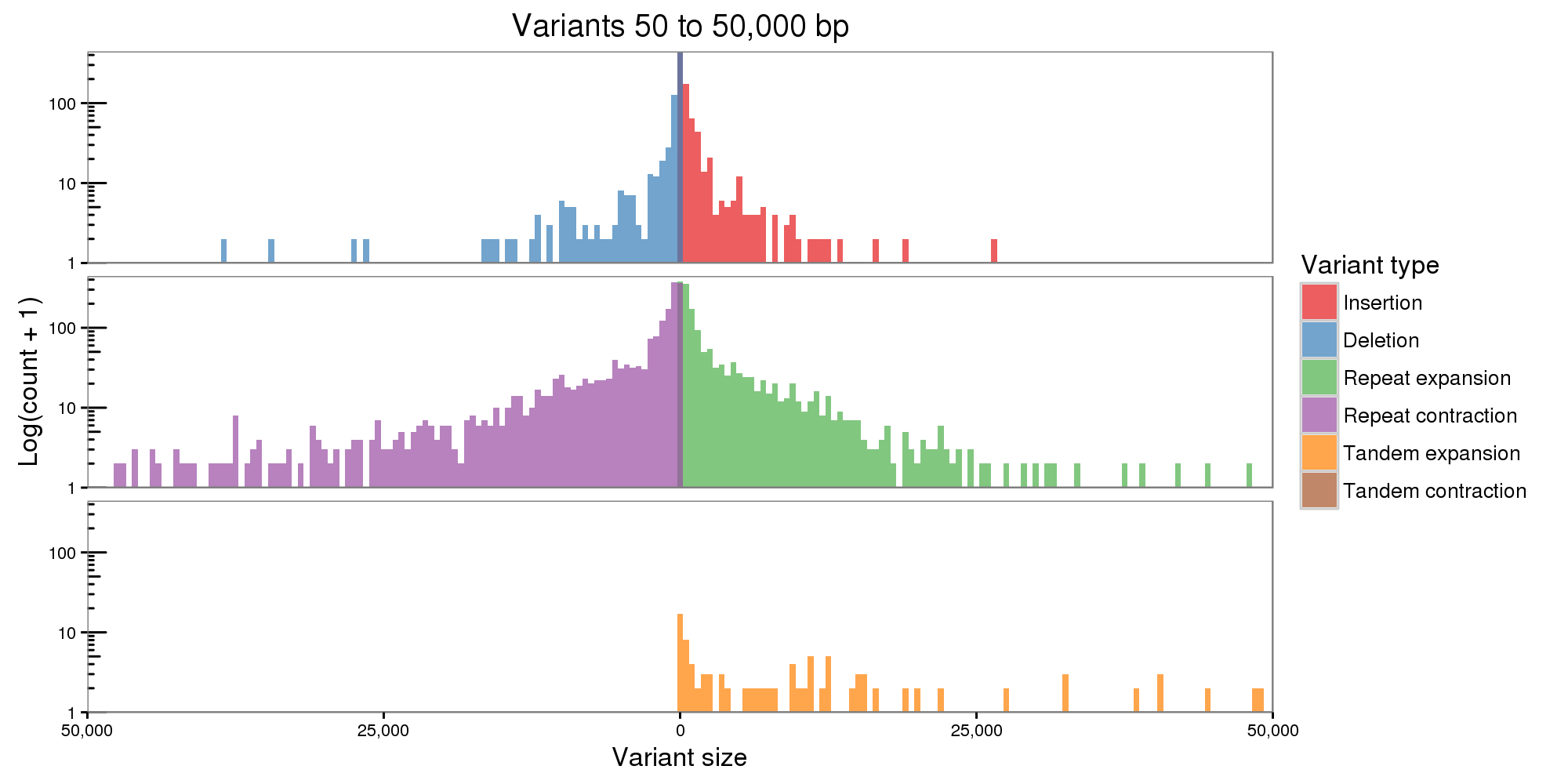


**Supplementary Figure 11. Distribution of haplotypic structural variant sizes when comparing the Phase1 to Phase0 assemblies.** The two assemblies were split at N’s and the contigs were aligned to identify structural variants (SV). Expanding the analysis from the default maximum of 10 kb to include variants up to 50 kb identified a further ~8 Mb of sequences affected by SV. Inclusion of longer SV sizes has a lower specificity.


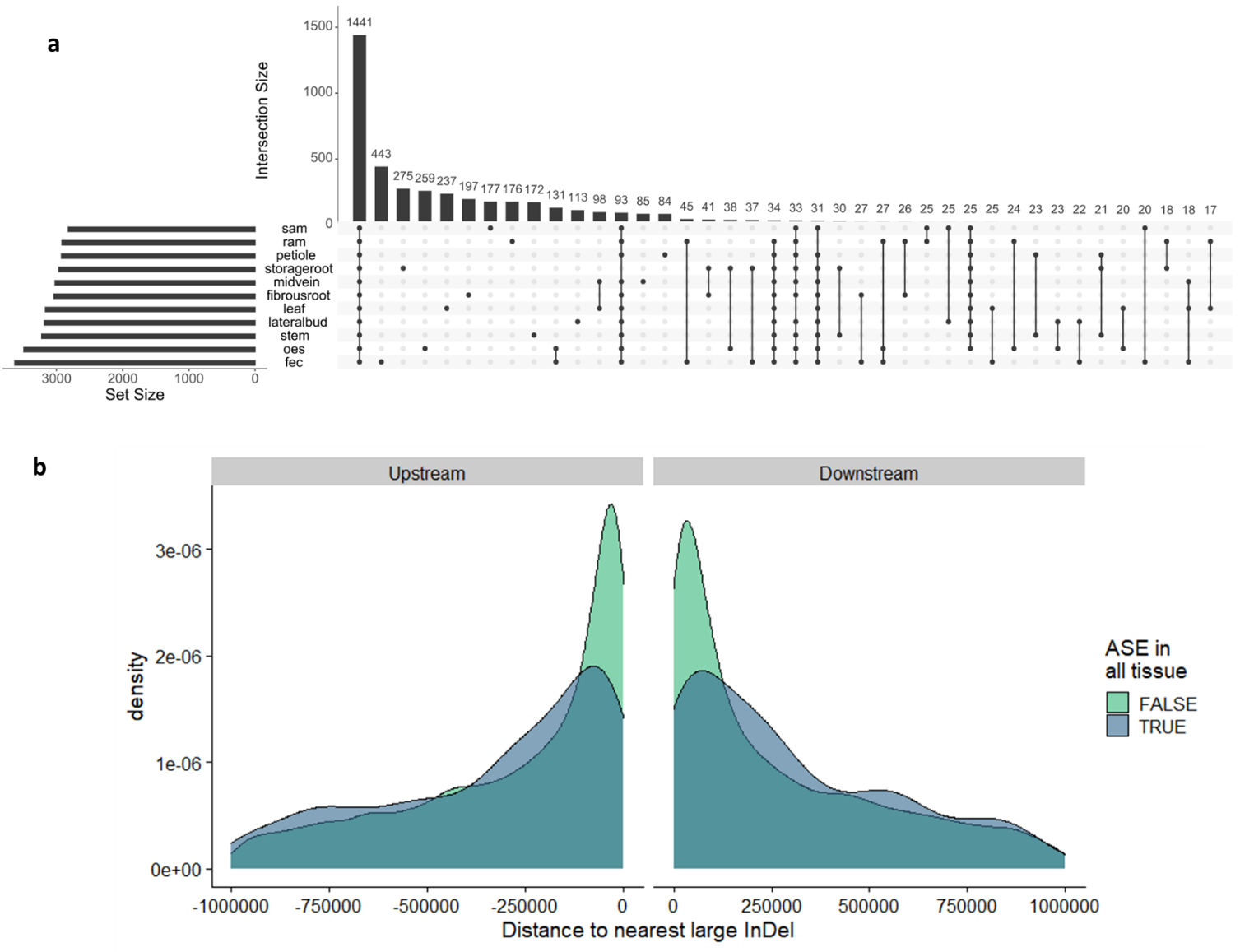


**Supplementary Figure 12. Potential effects of large haplotypic structural variants on allele specific expression. (A)** The overlap in genes identified as showing significant allele specific expression (ASE) between the different tissue types. (B) The distribution of distances (up to 1Mb) to the nearest large (> 50 bp) insertion or deletion (InDel) in genes that show “complete ASE” in all tissues vs. the rest of the transcriptome. If a gene shows greater than 5 log2(fold change) between allelic read counts the gene is characterized as having “complete ASE”.
