# Supplementary material for: Large structural variations in the haplotype‐resolved African cassava genome
Source: Plant J. 2021 Nov 10;108(6):1830–48. doi: 10.1111/tpj.15543 (PMC9299708; doi:10.1111/tpj.15543)
Supplement: Supplementary file 8 — File S7. Scripts for figures and assessment. [file TPJ-108-1830-s002.pdf]

TME7 Large structural variations in the haplotype resolved African cassava genome

Figures and analyses scripts

Ben N. Mansfeld Adam Boyher Jeffrey C. Berry Mark Wilson Shujun Ou
Seth Polydore Todd P. Michael Noah Fahlgren Rebecca S. Bart

6/23/2021

Load scripts and functions:

```
knitr::opts_chunk$set(echo = TRUE, cache = TRUE, warning=FALSE, message=FALSE)
library(tidyverse)
```

```
## -- Attaching packages --------------------------------------- tidyverse 1.3.1 --

## v ggplot2 3.3.4     v purrr   0.3.4
## v tibble  3.1.2     v dplyr   1.0.7
## v tidyr   1.1.3     v stringr 1.4.0
## v readr   1.4.0     v forcats 0.5.1

## -- Conflicts ------------------------------------------ tidyverse_conflicts() --
## x dplyr::filter() masks stats::filter()
## x dplyr::lag()    masks stats::lag()
```

```
library(UpSetR)

## Merqury color settings and functions
gray = "black"
red = "#E41A1C"
blue = "#377EB8" # light blue = "#56B4E9"
green = "#4DAF4A"
purple = "#984EA3"  # purple = "#CC79A7"
orange = "#FF7F00"  # orange = "#E69F00"
yellow = "#FFFF33"

merqury_col = c(gray, red, blue, green, purple, orange)
merqury_brw <- function(dat, direction=1) {
    merqury_colors=merqury_col[1:length(unique(dat))]
    if (direction == -1) {
        merqury_colors=rev(merqury_colors)
    }
    merqury_colors
}

ALPHA=0.4
LINE_SIZE=0.3

fancy_scientific <- function(d) {
```

```
    # turn in to character string in scientific notation
    d <- format(d, scientific = TRUE)
    # quote the part before the exponent to keep all the digits and turn the 'e+' into 10^ format
    d <- gsub("^(.*)e\\+", "'\\1'%*%10^", d)
    # convert 0x10^00 to 0
    d <- gsub("\\'0[\\.0]*\\'(.*)", "'0'", d)
    # return this as an expression
    parse(text=d)
}

format_theme <- function() {
    theme(legend.text = element_text(size=11),
          # legend.position = c(0.95,0.95),  # Modify this if the legend is covering your favorite circ
          legend.background = element_rect(size=0.1, linetype="solid", colour ="grey85"),
          legend.box.just = "right",
          legend.justification = c("right", "top"),
          axis.title=element_text(size=14,face="bold"),
          axis.text=element_text(size=12))
}

format_genomic <- function(...) {
      # Format a vector of numeric values according
      # to the International System of Units.
      # http://en.wikipedia.org/wiki/SI_prefix
      #
      # Based on code by Ben Tupper
      # https://stat.ethz.ch/pipermail/r-help/2012-January/299804.html
      # Args:
      #   ...: Args passed to format()
      #
      # Returns:
      #   A function to format a vector of strings using
      #   SI prefix notation
      #

      function(x) {
            limits <- c(1e0,   1e3, 1e6)
            #prefix <- c("","Kb","Mb")

            # Vector with array indices according to position in intervals
            i <- findInterval(abs(x), limits)

            # Set prefix to " " for very small values < 1e-24
            i <- ifelse(i==0, which(limits == 1e0), i)

            paste(format(round(x/limits[i], 1),
                         trim=TRUE, scientific=FALSE, ...)
                #  ,prefix[i]
            )
      }
}
```

Main text

Figure 1

```
fc <-
  read_csv(
    "Files for Figures/FlowCyto_080216.csv",
    skip = 21,
    skip_empty_rows = T,
    col_names = c("Line", "ID", "G0+G1", "Std", "DNA_Content")
  ) %>%
  fill(Line, ID) %>%
  separate(Line, into = c("Line", "Rep"), sep = " ") %>%
  mutate(Line = ifelse(Line == "Oko-iyawo", "TME7", Line))

fc_fig <- fc %>%
  filter(Line == "TME7") %>%
  ggplot(aes(
    x = Rep,
    y = DNA_Content / 2 * 1e3,
    group = Rep,
    fill = as.factor(Rep)
  )) +
  geom_boxplot() +
  geom_jitter(color = "black", width = 0.25) +
  labs(x = "Sample", y = "Weight (Mb C-Value)") +
  cowplot::theme_cowplot() +
  theme(axis.text.x = element_text(angle = 30, hjust = 1)) +
  cowplot::panel_border() +
  theme(legend.position = "none")
```

```
gs_specta <- cowplot::ggdraw() +
    cowplot::draw_image(image = "Files for Figures/genomescope1.png")
```

Make Figure 1:

```
cowplot::plot_grid(fc_fig, gs_specta, align = "h", labels = "auto", rel_widths = c(3, 7))
```

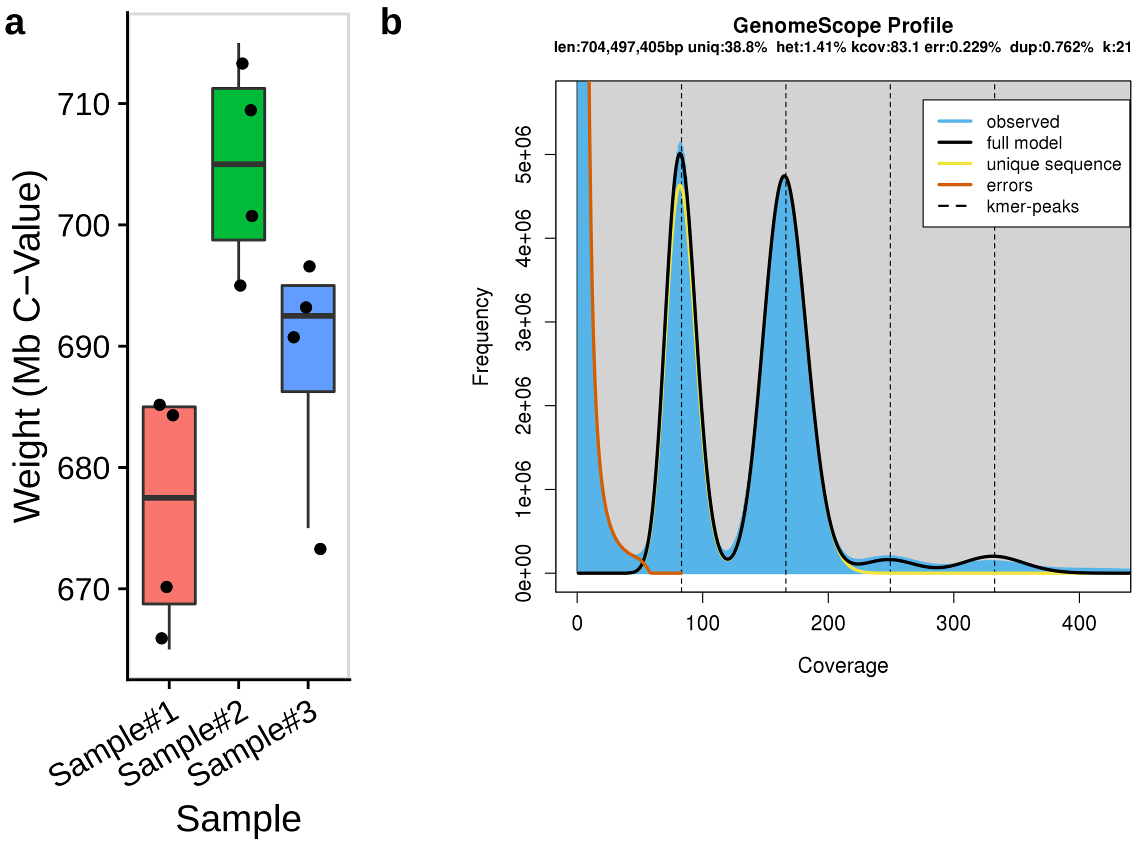

Figure 2

Load merqury data:

```
falcon_200703_sepcn <-
    bind_rows(
        "Primary" = read_tsv(
          file = "Files for Figures/merqury/200703_4000.p_ctg.spectra-cn.hist"),
        "Alternate" = read_tsv(
          file = "Files for Figures/merqury/200703_4000.a_ctg.spectra-cn.hist"),
        .id = "Phase"
    )

unzip_200703_sepcn <-
    bind_rows(
        "Primary" = read_tsv(
          file = "Files for Figures/merqury/tme7_200703_unzip.cns_p_ctg.spectra-cn.hist"),
        "Alternate" = read_tsv(
          file = "Files for Figures/merqury/tme7_200703_unzip.cns_h_ctg.spectra-cn.hist"),
        .id = "Phase"
    )

pilon_200703_sepcn <-
    bind_rows(
        "Primary" = read_tsv(
          file = "Files for Figures/merqury/tme7_200703_pilon.cns_p_ctg_pilon.spectra-cn.hist"),
```

```r
        "Alternate" = read_tsv(
          file = "Files for Figures/merqury/tme7_200703_pilon.cns_h_ctg_pilon.spectra-cn.hist"),
        .id = "Phase"
    )


purgedFullSRA_200703_sepcn <-
    bind_rows(
        "Primary" = read_tsv(file = "Files for Figures/merqury/purgeFullSRA1/purge_full_sra_manual.purg
        "Alternate" = read_tsv(file = "Files for Figures/merqury/purgeFullSRA1/purge_full_sra_manual.pu
        .id = "Phase"
    )

purgedFullSRArnd2_200703_sepcn <-
    bind_rows(
        "Primary" = read_tsv(file = "Files for Figures/merqury/purgeFullSRA2/primary__pd_rnd2_short_ful
        "Alternate" = read_tsv(file = "Files for Figures/merqury/purgeFullSRA2/primary__pd_rnd2_short_f
        .id = "Phase"
    )

pseudo_200703_sepcn <-
    bind_rows(
        "Primary" = read_tsv(file = "Files for Figures/merqury/phase_pseudo/phased_pseudohap_minaln500.
        "Alternate" = read_tsv(file = "Files for Figures/merqury/phase_pseudo/phased_pseudohap_minaln50
        .id = "Phase"
    )

phaseUnzip_200703_sepcn <-
    bind_rows(
        "Primary" = read_tsv(file = "Files for Figures/merqury/phase_unzip/phased_unzip_minaln500.phase
        "Alternate" = read_tsv(file = "Files for Figures/merqury/phase_unzip/phased_unzip_minaln500.pha
        .id = "Phase"
    )



all_sepcn <- bind_rows(
    "falcon" = falcon_200703_sepcn,
    "unzip_200703" = unzip_200703_sepcn,
    "pilon_200703" = pilon_200703_sepcn,
    # "purgedFullSRC_200703" = purgedFullSRA_200703_sepcn,
    "AddSRC_200703" = purgedFullSRArnd2_200703_sepcn,
    "phaseUnzip_200703" = phaseUnzip_200703_sepcn,
    "phasePseudo_200703" = pseudo_200703_sepcn,
    .id = "Version") %>%
    mutate(Version = fct_inorder(Version)) %>%
    separate(Version, into = c("Step", "Run"), sep = "_", remove = FALSE) %>%
    mutate(Step = fct_relevel(Step, "falcon", "unzip", "pilon", "AddSRC", "phaseUnzip"))
```

```r
falc_200703_cn <- read_tsv(
  file = "Files for Figures/merqury/200703_4000.spectra-cn.hist") %>%
    mutate(Copies = fct_relevel(Copies, "read-only"),
           Copies = fct_relevel(Copies, ">4", after = 5))
```

```r
unzip_200703_cn <- read_tsv(
  file = "Files for Figures/merqury/unzip/tme7_200703_unzip.spectra-cn.hist") %>%
    mutate(Copies = fct_relevel(Copies, "read-only"),
           Copies = fct_relevel(Copies, ">4", after = 5))

pilon_200703_cn <- read_tsv(
  file = "Files for Figures/merqury/pilon/tme7_200703_pilon.spectra-cn.hist") %>%
    mutate(Copies = fct_relevel(Copies, "read-only"),
           Copies = fct_relevel(Copies, ">4", after = 5))

SRC_200703_cn <- read_tsv(
  file = "Files for Figures/merqury/pilon+SRC/cns_p_h_ctg_pilon_SRC_fullAssemb.cns_p_h_ctg_pilon_SRC.spe
    mutate(Copies = fct_relevel(Copies, "read-only"),
           Copies = fct_relevel(Copies, ">4", after = 5))

purge_200703_cn <- read_tsv(file = "Files for Figures/merqury/purgeFullSRA2/primary__pd_rnd2_short_full
    mutate(Copies = fct_relevel(Copies, "read-only"),
           Copies = fct_relevel(Copies, ">4", after = 5))

phaseUnzip_200703_cn <- read_tsv(file = "Files for Figures/merqury/phase_unzip/phased_unzip_minaln500.sp
    mutate(Copies = fct_relevel(Copies, "read-only"),
           Copies = fct_relevel(Copies, ">4", after = 5))

phasePseudo_200703_cn <- read_tsv(file = "Files for Figures/merqury/phase_pseudo/phased_pseudohap_minal
    mutate(Copies = fct_relevel(Copies, "read-only"),
           Copies = fct_relevel(Copies, ">4", after = 5))
```

```r
all_spectra_cn <- bind_rows(
    "falcon" = falc_200703_cn,
    "unzip" = unzip_200703_cn,
    "pilon" = pilon_200703_cn,
    "SRC" = SRC_200703_cn,
    "purge_dups" = purge_200703_cn,
    "phaseUnzip" = phaseUnzip_200703_cn,
    "phasePseudo" = phasePseudo_200703_cn,
    # "Add_SRC" = sra_200703_cn,
    .id = "Version") %>%
    mutate(Version = fct_inorder(Version))
```

```r
f200703_asm <-
    read_tsv("Files for Figures/merqury/200703_4000.spectra-asm.hist") %>%
    bind_rows(
        read_tsv(
            "Files for Figures/merqury/200703_4000.dist_only.hist",
            col_names = colnames(.)
        )
    ) %>%
    mutate(
        Assembly = case_when(
            Assembly == "a_ctg-only" ~ "Alternate-only",
            Assembly == "p_ctg-only" ~ "Primary-only",
            TRUE ~ Assembly
        )
    ) %>%
```

```
    mutate(Assembly = fct_relevel(
        Assembly,
        "shared",
        "Alternate-only",
        "Primary-only",
        "read-only"
    ))


u200703_asm <-
    read_tsv("Files for Figures/merqury/tme7_200703_unzip.spectra-asm.hist") %>%
    bind_rows(
        read_tsv(
            "Files for Figures/merqury/tme7_200703_unzip.dist_only.hist",
            col_names = colnames(.)
        )
    ) %>%
    mutate(
        Assembly = case_when(
            Assembly == "cns_h_ctg-only" ~ "Alternate-only",
            Assembly == "cns_p_ctg-only" ~ "Primary-only",
            TRUE ~ Assembly
        )
    ) %>%
    mutate(Assembly = fct_relevel(
        Assembly,
        "shared",
        "Alternate-only",
        "Primary-only",
        "read-only"
    ))


p200703_asm <-
    read_tsv("Files for Figures/merqury/tme7_200703_pilon.spectra-asm.hist") %>%
    bind_rows(
        read_tsv(
            "Files for Figures/merqury/tme7_200703_pilon.dist_only.hist",
            col_names = colnames(.)
        )
    ) %>%
    mutate(
        Assembly = case_when(
            Assembly == "cns_h_ctg_pilon-only" ~ "Alternate-only",
            Assembly == "cns_p_ctg_pilon-only" ~ "Primary-only",
            TRUE ~ Assembly
        )
    ) %>%
    mutate(Assembly = fct_relevel(
        Assembly,
        "shared",
        "Alternate-only",
        "Primary-only",
```

```
        "read-only"
    ))


purge200703_asm <-
    read_tsv("Files for Figures/merqury/purgeFullSRA1/purge_full_sra_manual.spectra-asm.hist") %>%
    bind_rows(
        read_tsv(
            "Files for Figures/merqury/purgeFullSRA1/purge_full_sra_manual.dist_only.hist",
            col_names = colnames(.)
        )
    ) %>%
    mutate(
        Assembly = case_when(
            Assembly == "purge_full_sra_manual_old.hap-only" ~ "Alternate-only",
            Assembly == "purge_full_sra_manual_old.purged-only" ~ "Primary-only",
            TRUE ~ Assembly
        )
    ) %>%
    mutate(Assembly = fct_relevel(
        Assembly,
        "shared",
        "Alternate-only",
        "Primary-only",
        "read-only"
    ))

purgedX2_200703_asm <-
    read_tsv(
        "Files for Figures/merqury/purgeFullSRA2/primary__pd_rnd2_short_full.spectra-asm.hist"
    ) %>%
    bind_rows(
        read_tsv(
            "Files for Figures/merqury/purgeFullSRA2/primary__pd_rnd2_short_full.dist_only.hist",
            col_names = colnames(.)
        )
    ) %>%
    mutate(
        Assembly = case_when(
            Assembly == "purged-only" ~ "Alternate-only",
            Assembly == "purge_full_sra_manual.purged-only" ~ "Primary-only",
            TRUE ~ Assembly
        )
    ) %>%
    mutate(Assembly = fct_relevel(
        Assembly,
        "shared",
        "Alternate-only",
        "Primary-only",
        "read-only"
    ))

phase_200703_asm <-
```

```
    read_tsv("Files for Figures/merqury/phase_unzip/phased_unzip_minaln500.spectra-asm.hist") %>%
    bind_rows(
        read_tsv(
            "Files for Figures/merqury/phase_unzip/phased_unzip_minaln500.dist_only.hist",
            col_names = colnames(.)
        )
    ) %>%
    mutate(
        Assembly = case_when(
            Assembly == "phased.unzip.1-only" ~ "Alternate-only",
            Assembly == "phased.unzip.0-only" ~ "Primary-only",
            TRUE ~ Assembly
        )
    ) %>%
    mutate(Assembly = fct_relevel(
        Assembly,
        "shared",
        "Alternate-only",
        "Primary-only",
        "read-only"
    ))

pseudo_200703_asm <-
    read_tsv(
        "Files for Figures/merqury/phase_pseudo/phased_pseudohap_minaln500.spectra-asm.hist"
    ) %>%
    bind_rows(
        read_tsv(
            "Files for Figures/merqury/phase_pseudo/phased_pseudohap_minaln500.dist_only.hist",
            col_names = colnames(.)
        )
    ) %>%
    mutate(
        Assembly = case_when(
            Assembly == "phased.1-only" ~ "Alternate-only",
            Assembly == "phased.0-only" ~ "Primary-only",
            TRUE ~ Assembly
        )
    ) %>%
    mutate(Assembly = fct_relevel(
        Assembly,
        "shared",
        "Alternate-only",
        "Primary-only",
        "read-only"
    ))


all_spectra_asm <- bind_rows(
     "falcon_200703" = f200703_asm,
     "unzip_200703" = u200703_asm,
     "pilon_200703" = p200703_asm,
     #"purgedups_200703" = purge200703_asm,
```

```
     "purgedupsX2_200703" = purgedX2_200703_asm,
     "phaseUnzip_200703" = phase_200703_asm,
     "phasePseudo_200703" = pseudo_200703_asm,
    # "purgedups+SRA_200703" = purged_SRC_200703_asm,
    # "purgedupsfull_200703" = manualpurgefull_200703_asm,
    # "purgedupsfullX2_200703" = manualpurgeX2_200703_asm,
    .id = "Version") %>%
    mutate(Version = fct_inorder(Version)) %>%
    separate(Version, into = c("Step", "Run"), sep = "_", remove = FALSE) %>%
    mutate(Step = fct_relevel(Step, "falcon", "unzip", "pilon", "purgedupsX2", "phase"))
```

```
p1 <- all_sepcn %>%
    mutate(Copies = fct_relevel(Copies, "read-only"),
           Copies = fct_relevel(Copies, ">4", after = 5)) %>%
    filter(Step == "phaseUnzip") %>%
    ggplot(aes(x=kmer_multiplicity, y=Count, color=Copies)) +
    geom_line(size = 0.5) +
    scale_color_manual(values = merqury_brw(all_sepcn$Copies,direction = 1), name="Times in\nassembly")
    cowplot::theme_cowplot() +
    cowplot::panel_border() +
    format_theme() +
    scale_y_continuous(labels=fancy_scientific) +
    coord_cartesian(xlim=c(0, 430), ylim=c(0, 5e6)) +
    facet_grid(~ Phase) +
    theme(legend.position = c(0.95,0.95)) +
    labs(x = "k-mer multiplicity")


p2 <- all_spectra_cn %>%
    filter(Version == "phaseUnzip") %>%
    mutate(Copies = fct_rev(Copies)) %>%
    ggplot(aes(x=kmer_multiplicity, y=Count, color=Copies, fill=Copies)) +
    geom_area(alpha = 0.4) +
    scale_color_manual(values = merqury_brw(all_spectra_cn$Copies, direction=-1),
                       name="Times in\nassembly",
                       breaks=rev(levels(all_spectra_cn$Copies))) +
    scale_fill_manual(values = merqury_brw(all_spectra_cn$Copies, direction=-1),
                      name="Times in\nassembly",
                      breaks=rev(levels(all_spectra_cn$Copies))) +
    cowplot::theme_cowplot() +
    cowplot::panel_border() +
    format_theme() +
    scale_y_continuous(labels=fancy_scientific) +
    coord_cartesian(xlim=c(0, 430), ylim=c(0, 5e6)) +
    theme(legend.position = c(0.95,0.95)) +
    labs(x = "k-mer multiplicity")

p3 <- all_spectra_asm %>%
    filter(Step == "phaseUnzip") %>%
    filter(kmer_multiplicity > 0) %>%
ggplot(aes(x=kmer_multiplicity, y = Count, color=Assembly, fill=Assembly)) +
    geom_area(alpha = 0.4) +
    # geom_bar(data = all_spectra_asm %>% filter(kmer_multiplicity == 0), aes(x = 0),
    #          position="stack", stat="identity", show.legend = FALSE, width = 3, alpha = 0.4) +
```

```
    scale_color_manual(values = merqury_brw(all_spectra_asm$Assembly, direction=-1), name="Phase specif
    scale_fill_manual(values = merqury_brw(all_spectra_asm$Assembly, direction=-1), name="Phase specific
    cowplot::theme_cowplot() +
    cowplot::panel_border() +
    format_theme() +
    scale_y_continuous(labels=fancy_scientific) +
    coord_cartesian(xlim=c(0, 430), ylim=c(0, 5e6)) +
    theme(legend.position = c(0.95,0.95)) +
    labs(x = "k-mer multiplicity")

bottom <- cowplot::plot_grid(p2, p3, nrow = 1, labels = c("b", "c"))

pdf("fig2.pdf", width = 8, height = 8)
cowplot::plot_grid(p1, bottom,  nrow = 2, align = 'V', axis = 'l', labels = c("a", ""))
dev.off()
```

```
## pdf
##   2
```

```
cowplot::plot_grid(p1, bottom,  nrow = 2, align = 'V', axis = 'l', labels = c("a", ""))
```

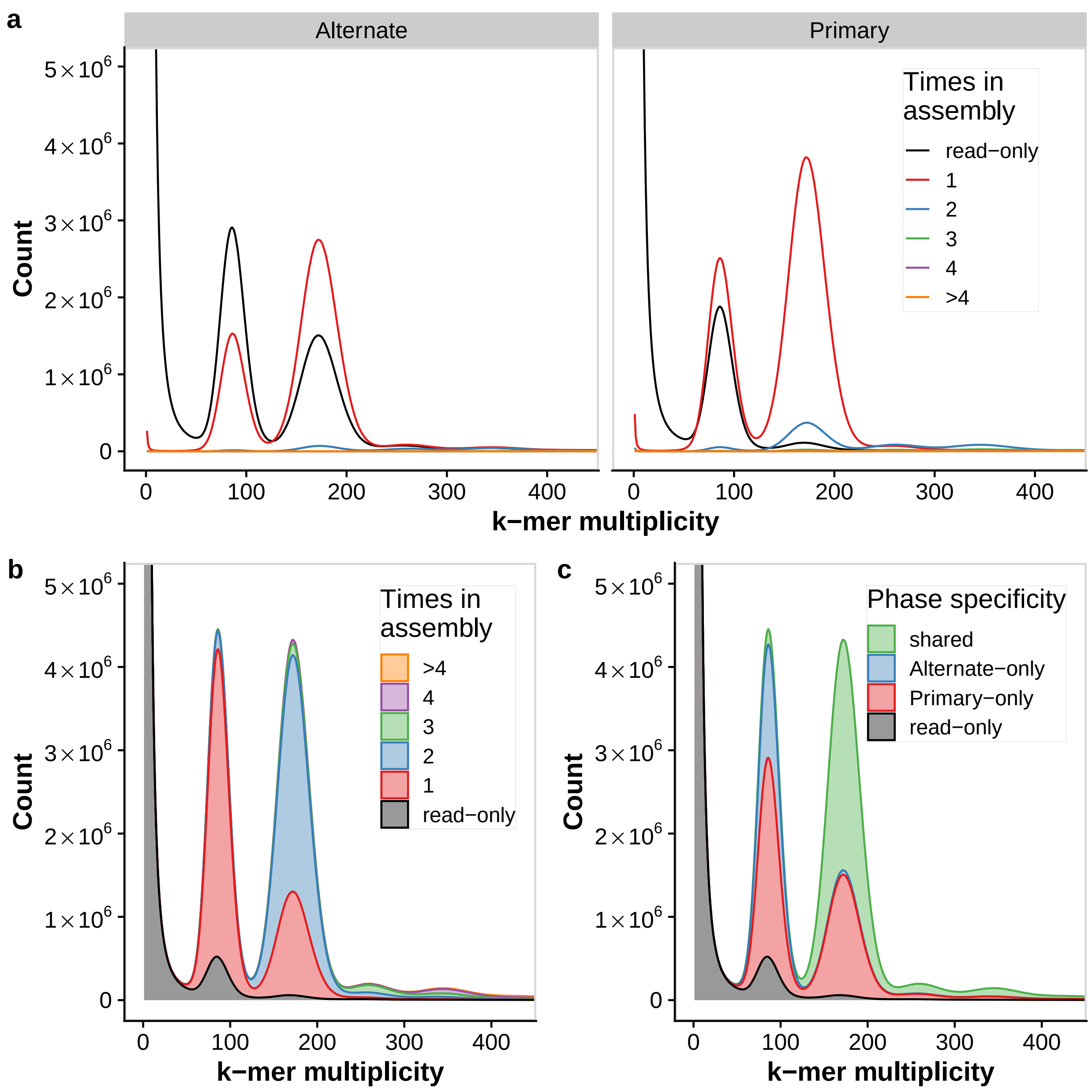

Figure 3:

```
hic <- read_tsv(file = "Files for Figures/hic/GInterations500.tsv",
                col_names = c("xchrom", "xstart", "xend", "ychrom", "ystart", "yend", "interactions"))

hic <- hic %>% filter(grepl("Chr", xchrom),
                      grepl("Chr", ychrom))


hic_chrm_lengths_x <-
    hic %>% group_by(xchrom) %>% summarise(length = max(xend)) %>%
    ungroup() %>%
    mutate(xpad = lag(length, default = 0),
```

```
            xcumsumpad = cumsum(xpad),
            labelpos_x = xcumsumpad + length/2,
            label_x = paste0("Chr\n", str_extract(xchrom, "[0-9][0-9]")))

hic_chrm_lengths_y <-
    hic %>% group_by(ychrom) %>% summarise(length = max(yend)) %>%
    ungroup() %>%
    mutate(ypad = lag(length, default = 0),
            ycumsumpad = cumsum(ypad),
            labelpos_y = ycumsumpad + length/2,
            label_y = paste0("Chr\n", str_extract(ychrom, "[0-9][0-9]")))

hic_mat <- hic %>%
    left_join(hic_chrm_lengths_x, by = "xchrom") %>%
    left_join(hic_chrm_lengths_y, by = c("ychrom"))


 hic_plot <- hic_mat %>%
    ggplot() +
    geom_tile(
        aes(
            x = xstart + xcumsumpad,
            y = ystart + ycumsumpad,
            fill = log10(interactions)
        ),
        width = 500000,
        height = 500000
    ) +
    geom_tile(
        aes(
            y = xstart + xcumsumpad,
            x = ystart + ycumsumpad,
            fill = log10(interactions)
        ),
        width = 500000,
        height = 500000
    ) +
    geom_vline(data = hic_chrm_lengths_x,
               aes(xintercept = xcumsumpad),
               linetype = 2) +
    geom_hline(data = hic_chrm_lengths_x,
               aes(yintercept = xcumsumpad),
               linetype = 2) +
    geom_text(data = hic_chrm_lengths_x, aes(x = labelpos_x, y = -20e6, label = label_x)) +
    labs(x = "Phase0", y = "Phase0") +
    scale_fill_viridis_c() +
    coord_fixed() +
    cowplot::theme_cowplot() +
    theme(legend.position = c(0.75, 0.01))
```

```
map <- read_tsv(file = "Files for Figures/marker_alignment/mesculenta_map_2014.txt") %>%
    mutate(CHROM = paste0(
        "Chromosome",
        stringr::str_pad(
```

```
            gtools::roman2int(chromosome),
            width = 2,
            pad = "0"
        )
    )) %>%
    rename(LG = chromosome)


header <- c(
    "query_id",
    "ref_id",
    "perc_ident",
    "alignment_length",
    "mismatch",
    "num_gaps",
    "query_start",
    "query_end",
    "ref_start",
    "ref_end",
    "evalue",
    "bitscore"
)

blast_pri <-
    read_tsv(
      file = "Files for Figures/marker_alignment/cassava_tme7_phase0_scaffolded_renamed_2.linkage_map.bl[illegible]
             col_names = header) %>%
    # distinct(query_id, .keep_all = TRUE) %>%
    right_join(map, by = c("query_id" = "SGN id"))


blast_alt <- read_tsv(
  file = "Files for Figures/marker_alignment/p1_pseudohap_contigs.linkage_map_blast.txt",
                      col_names = header) %>%
    # distinct(query_id, .keep_all = TRUE) %>%
    right_join(map, by = c("query_id" = "SGN id"))


markers <- bind_rows("phase0_scaffolded" = blast_pri,
                     "phase1_contigs" = blast_alt,
                     .id = "phase")

markers_count <- markers %>%
    add_count(phase, query_id, .drop = FALSE, name = "nhits") %>%
    mutate(nhits = ifelse(nhits >= 10, 11, nhits),
           nhits = ifelse(is.na(ref_id), 0, nhits)) %>%
    mutate(qual =  (perc_ident >= 95 &
               alignment_length >= 150))

# filter qual
markers_count_filt <- markers_count %>%
    filter(qual) %>%
    add_count(phase, query_id, .drop = FALSE, name = "nhits_postfilter") %>%
```

```
    mutate(nhits_postfilter = ifelse(nhits_postfilter >= 10, 11, nhits_postfilter),
           nhits_postfilter = ifelse(is.na(ref_id), 0, nhits_postfilter))
    # mutate(ref_id = fct_relevel(ref_id, as.character(chrm_order) ))

# summarize post filter
markers_count_filt %>%
    group_by(phase, query_id) %>%
    summarise(count = unique(nhits_postfilter)) %>%
    group_by(phase, count) %>%
    summarize(nMarkers = n_distinct(query_id)) %>%
    group_by(phase) %>%
    mutate(percent = (nMarkers / sum(nMarkers)) * 100,
           count = ifelse(count == 11, "10+", count)) %>%
    pivot_wider(names_from = phase, values_from = c(nMarkers, percent)) %>%
    knitr::kable()
```

count	nMarkers_phase0_scaffolded	nMarkers_phase1_contigs	percent_phase0_scaffolded	percent_phase1_contigs
1	19252	19228	88.6290397	88.6409736
2	1941	1928	8.9356413	8.8880693
3	242	267	1.1140779	1.2308685
4	98	76	0.4511555	0.3503596
5	29	27	0.1335052	0.1244699
6	17	18	0.0782617	0.0829799
7	10	18	0.0460363	0.0829799
8	11	12	0.0506399	0.0553199
9	13	8	0.0598472	0.0368800
10+	109	110	0.5017954	0.5070994

```
# corr for each chrom
markers_count_filt %>%
    filter(phase == "phase0_scaffolded") %>%
    filter(nhits_postfilter < 10) %>%
    group_by(phase, ref_id) %>%
    summarise(cor(ref_start, position, method = "spearman"))
```

```
## # A tibble: 560 x 3
## # Groups:   phase [1]
##    phase            ref_id            `cor(ref_start, position, method = "spear~
##    <chr>            <chr>                                                  <dbl>
##  1 phase0_scaffold~ Chromosome01_Pha~                                      0.986
##  2 phase0_scaffold~ Chromosome02_Pha~                                      0.946
##  3 phase0_scaffold~ Chromosome03_Pha~                                      0.959
##  4 phase0_scaffold~ Chromosome04_Pha~                                      0.979
##  5 phase0_scaffold~ Chromosome05_Pha~                                      0.984
##  6 phase0_scaffold~ Chromosome06_Pha~                                      0.983
##  7 phase0_scaffold~ Chromosome07_Pha~                                      0.941
##  8 phase0_scaffold~ Chromosome08_Pha~                                      0.966
##  9 phase0_scaffold~ Chromosome09_Pha~                                      0.983
## 10 phase0_scaffold~ Chromosome10_Pha~                                      0.970
## # ... with 550 more rows
```

```
### all correlation
```

```r
markers_count_filt %>%
    filter(phase == "phase0_scaffolded") %>%
    filter(nhits_postfilter < 10) %>%
    group_by(phase, ref_id) %>%
    summarise(correl = cor(ref_start, position, method = "spearman")) %>% filter(grepl(x = ref_id,patte
```

```
## # A tibble: 1 x 2
##   phase              `mean(correl)`
##   <chr>                       <dbl>
## 1 phase0_scaffolded           0.966
```

```r
# plot
linkage_plot <- markers_count_filt %>%
    mutate(LG = fct_relevel(LG, as.character(as.roman(1:18)))) %>%
    filter(phase == "phase0_scaffolded") %>%
    filter(nhits_postfilter < 10) %>%
    filter(grepl("Chrom", ref_id)) %>%
    ggplot() +
    geom_point(aes(x = ref_start, y = position, color = LG)) +
    facet_grid(paste0("Chr", str_extract(ref_id, pattern = "[0-9][0-9]")) ~ ., scales = "free") +
    labs(x = "Physical position (Mb)", y = "Genetic distance (cM)") +
    cowplot::theme_cowplot() +
    cowplot::panel_border() +
    #theme(legend.position = "bottom", axis.text.x = element_text(angle = 45)) +
    guides(colour = guide_legend("Linkage group\nin map", ncol = 1)) +
    scale_x_continuous(labels = format_genomic()) +
    scale_color_viridis_d()
```

```r
pdf("fig3.pdf", width = 14, height = 10)
cowplot::plot_grid(hic_plot + theme(legend.background = element_rect(fill = "white"), legend.position =
dev.off()
```

```
## pdf
##   2
```

Figure 4

```r
all_buscos <- read_tsv("Files for Figures/busco/all_busco.tsv.txt", col_names = c("dir", "results")) %>%
    separate(dir, into = "Version", sep = "/") %>%
    separate(results, into = c("Complete", "Duplicated", "Fragmented", "Missing", "n"), sep = ",") %>%
    separate(Complete, into = c("Complete", "Single"), sep = "\\[") %>%
    mutate(Complete =      as.numeric(gsub("[^0-9.-]", "", Complete)),
           Single =        as.numeric(gsub("[^0-9.-]", "", Single)),
           Duplicated =    as.numeric(gsub("[^0-9.-]", "", Duplicated)),
           Fragmented = as.numeric(gsub("[^0-9.-]", "", Fragmented)),
           Missing =       as.numeric(gsub("[^0-9.-]", "", Missing)),
           n =             as.numeric(gsub("[^0-9.-]", "", n))
           ) %>%
    mutate(Step = c(rep("Falcon", 3),
                    rep("Unzip", 3),
                    rep("Pilon", 3),
                    "Add SRC",
                    rep("Purge", 3),
                    rep("Phase_Unzip", 3),
```

```
                    rep("Phase_Pseudohap", 2),
                    rep("Phase0_scaffolded", 3)
                    ),
            Assembly = case_when(
                Version %in% c("BUSCO_2__a_ctg", "BUSCO_4__cns_h_ctg", "BUSCO_5__cns_h_ctg_pilon", "BUSC
                Version %in% c("BUSCO_2__p_ctg", "BUSCO_4__cns_p_ctg", "BUSCO_5__cns_p_ctg_pilon", "BUSC
                Version == "BUSCO_5__cns_h_p_ctg_pilon_SRA" ~ "Full",
                Version == "BUSCO_9__TME7_p0_p1_Unzip" ~ "Full (Unzip)",
                Version == "BUSCO_9__TME7_p0_p1_Pseudohap" ~ "Full (Pseudohap)",
                TRUE ~ "Full"
            )) %>%
    mutate(Step = fct_inorder(Step),
           Assembly = fct_relevel(Assembly, "Alternate", "Primary", "Full", "Full_Unzip", "Full_Pseudoha
    gather(-Version, -Step, -Assembly, key = "Category", value = "Value") %>%
    filter(Category != "n") %>%
    mutate(Number = ceiling(Value / 100 * 1614)) %>%
    mutate(label = paste0(round(Number / 1614 * 100, digits = 2), "%")) %>%
    mutate(Category = fct_rev(fct_relevel(Category, "Single", "Duplicated", "Fragmented", "Missing")))


#line just AP
AP <- all_buscos %>%
    filter(Category != "Complete") %>%
    filter(Assembly %in% c( "Alternate", "Primary")) %>%
    mutate(Assembly = case_when(grepl("Full", Assembly) ~ "Full",
                                TRUE ~ as.character(Assembly))) %>%
    ggplot(aes(x = Step, y = Number/1614*100, color = Assembly, group = Assembly)) +
    #geom_bar(stat = "identity", position = "stack") +
    geom_line() +
    geom_point(size = 2) +
    facet_grid(~ Category) +
    cowplot::theme_cowplot() +
    theme(axis.text.x = element_text(angle = 40, hjust = 1)) +
    labs(y = "Percent") +
    cowplot::panel_border()

# Just full assems
full <- all_buscos %>%
    filter(Category != "Complete") %>%
    filter(grepl("Full", Assembly)) %>%
    # mutate(Step = case_when(Assembly == "Full_SRC" ~ "Add SRC",
    #                          TRUE ~ as.character(Step))) %>%
    # filter(Assembly %in% c( "Alternate", "Primary")) %>%
    # mutate(Assembly = case_when(grepl("Full", Assembly) ~ "Full",
    #                             TRUE ~ as.character(Assembly))) %>%
    mutate(Step = case_when(
        Step == "Phase_Unzip" ~ "Falcon-Phase contigs + Unzip haplotigs",
        Assembly == "Full (Unzip)" ~ "Scaffolded + Unzip haplotigs",
        Assembly == "Full (Pseudohap)" ~ "Scaffolded + Pseuodhap haplotigs",
        TRUE ~ as.character(Step))
    ) %>%
    mutate(Step = fct_inorder(Step)) %>%
    ggplot(aes(x = Step, y = Number, color = Category, fill = Category, group = Category)) +
```

```
    geom_bar(stat = "identity", position = "stack") +
    geom_text(aes(label = label), size = 3, color = "black", stat = "identity", position = position_stac
    # geom_line() +
    # geom_point() +
    # facet_grid(~ Category) +
    cowplot::theme_cowplot() +
    theme(axis.text.x = element_text(angle = 30, hjust = 1)) +
    # labs(y = "Percent") +
    cowplot::panel_border()
```

```
phase0_busco <-
    read_tsv(
        file = "Files for Figures/busco/BUSCO_full_table_phase0_unzip.tsv",
        skip = 3,
        col_names = c(
            "Busco id",
            "Status",
            "Sequence",
            "Gene Start",
            "Gene End",
            "Score",
            "Length",
            "OrthoDB url",
            "Description"
        )
    ) %>%
    distinct(`Busco id`, .keep_all = TRUE)

phase1_busco <-
    read_tsv(
        file = "Files for Figures/busco/BUSCO_full_table_phase1_unzip.tsv",
        skip = 3,
        col_names = c(
            "Busco id",
            "Status",
            "Sequence",
            "Gene Start",
            "Gene End",
            "Score",
            "Length",
            "OrthoDB url",
            "Description"
        )
    ) %>%
    distinct(`Busco id`, .keep_all = TRUE)

full_buscos <- phase0_busco %>%
    bind_rows("phase0" = ., "phase1" = phase1_busco, .id = "phase") %>%
    mutate(set = paste(phase, Status, sep = "_")) %>%
    select(set, `Busco id`) %>%
    arrange(set) %>%
    mutate(i = 1) %>%
    spread(set, value = i, fill = 0) %>%
    select(contains("Comp"), contains("Dup"), contains("Frag"), contains("Miss"))
```

```
# svg(filename = "busco_ovlp.svg", width = 18, height = 6)
# upset(as.data.frame(full_buscos), sets = colnames(full_buscos), keep.order = T, mb.ratio = c(0.60, 0.
# dev.off()
```

```
busco_top <- cowplot::plot_grid(AP, full, nrow = 1, rel_widths = c(1, 0.6), align = "hv", axis = "b", la
pdf(file = "fig4.pdf", width = 16, height = 12)
cowplot::plot_grid(busco_top, cowplot::ggdraw() +
                     cowplot::draw_image(image = "busco_ovlp.svg"),
                   nrow = 2,
                   rel_heights = c(1.25, 0.75),
                   labels = c("", "c"))
dev.off()
```

```
## pdf
##   2
```

```
cowplot::plot_grid(busco_top, cowplot::ggdraw() +
                     cowplot::draw_image(image = "busco_ovlp.svg"),
                   nrow = 2,
                   rel_heights = c(1.25, 0.75),
                   labels = c("", "c"))
```

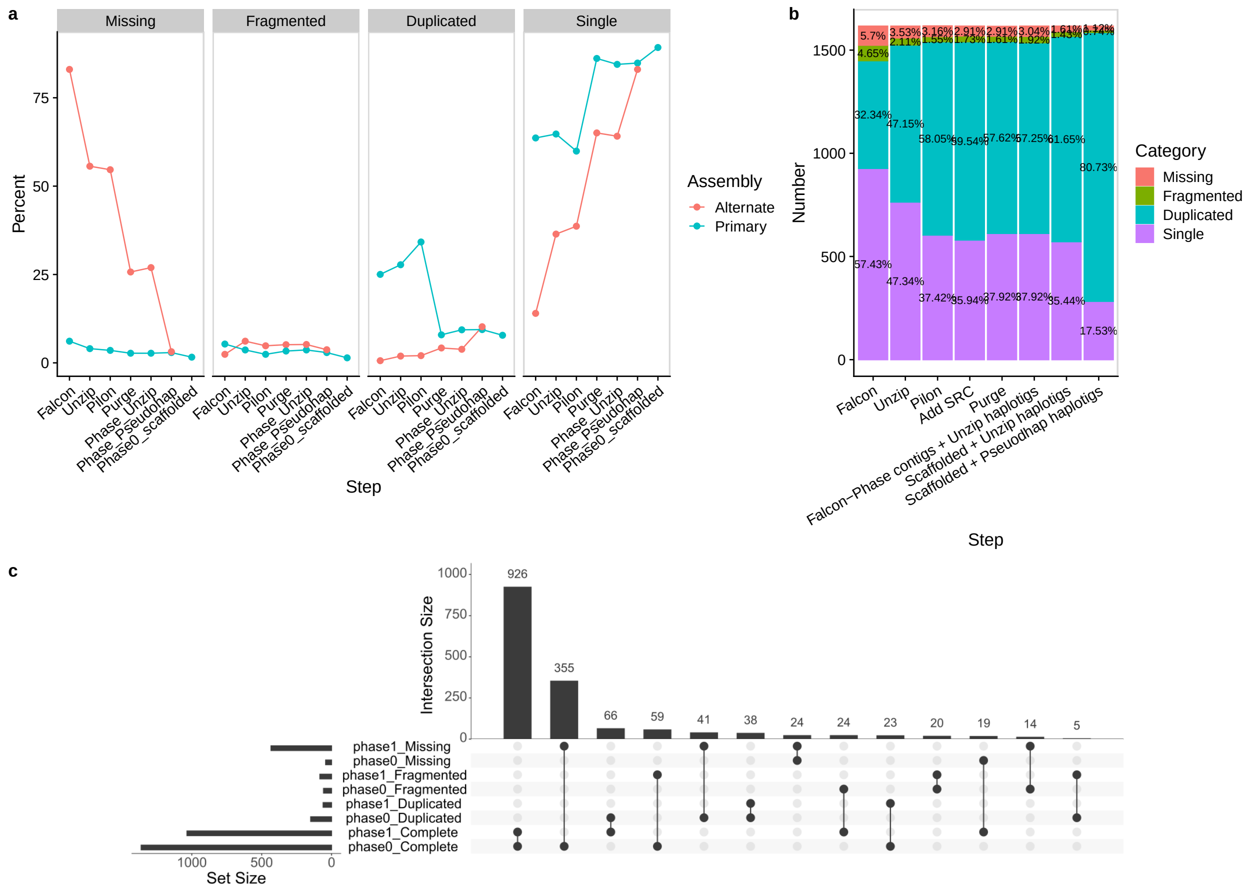

Figure 5

```
SV_p1 <-
    read_tsv(file = "Files for Figures/variation/cassava_tme7_phase1_unzip_contigs_vs_p0_scaffolds_spli
    separate(reference, into = c("Chrm", "coords"), sep = ":") %>%
    separate(
        coords,
        into = c("ctg_start", "ctg_end"),
        sep = "-",
        convert = TRUE
    ) %>%
    mutate(
        SV_start = ctg_start + ref_start,
        SV_end = ctg_start + ref_stop,
        SV_cmd = paste0(
            "-o ",
            Chrm,
            "_",
            SV_start,
            "_",
            SV_end,
            " -c ",
            Chrm,
            " -s ",
            SV_start,
            " -e ",
            SV_end
        )
    ) %>%
    mutate(query_coordinates = ifelse(
        str_count(query_coordinates, ":") == 2,
        sub(":", ":0-0:",
            query_coordinates),
        query_coordinates
    )) %>%
    separate(
        query_coordinates,
        into = c(
            "query_Chrm",
            "query_ctg_pos",
            "query_int_pos",
            "query_strand"
        ),
        sep = ":"
    ) %>%
    separate(
        col = query_ctg_pos,
        into = c("query_ctg_start", "query_ctg_end"),
        sep = "-",
        convert = T
    ) %>%
    separate(
        col = query_int_pos,
        into = c("query_int_start", "query_int_end"),
```

```
        sep = "-",
        convert = T
    ) %>%
    mutate(
        query_SV_start = query_ctg_start + query_int_start,
        query_SV_end = query_ctg_start + query_int_end
    )

write_delim(
    x = SV_p1 %>%  select(
        Chrm,
        SV_start,
        SV_end,
        everything(),
        -SV_cmd,
        -ctg_start,
        -ctg_end,
        -ref_start,
        -ref_stop,
        -contains("query_ctg"),
        -contains("query_int")
    ),
    "Supplementary_file_2_TME7_Phase1_vs_Phase0_SVs.tsv",
    delim = "\t"
)

SV_p1 %>% select(Chrm, SV_start, SV_end, type, size) %>%
    filter(type %in% c("Insertion", "Deletion"),!is.na(SV_start)) %>%
    arrange(Chrm, SV_start) %>% write_delim("SV_p1_indels.bed", delim = "\t", col_names = F)

SV_p1 %>% dplyr::select(Chrm, SV_start, SV_end, type, size) %>%
    filter(!is.na(SV_start)) %>% arrange(Chrm, SV_start) %>%
    write_delim("SV_p1_allSVs.bed", delim = "\t", col_names = F)
```

```
readDelta <- function(deltafile){
    lines = scan(deltafile, 'a', sep='\n', quiet=TRUE)
    lines = lines[-1]
    lines.l = strsplit(lines, ' ')
    lines.len = lapply(lines.l, length) %>% as.numeric
    lines.l = lines.l[lines.len != 1]
    lines.len = lines.len[lines.len != 1]
    head.pos = which(lines.len == 4)
    head.id = rep(head.pos, c(head.pos[-1], length(lines.l)+1)-head.pos)
    mat = matrix(as.numeric(unlist(lines.l[lines.len==7])), 7)
    res = as.data.frame(t(mat[1:5,]))
    colnames(res) = c('rs','re','qs','qe','error')
    res$qid = unlist(lapply(lines.l[head.id[lines.len==7]], '[', 2))
    res$rid = unlist(lapply(lines.l[head.id[lines.len==7]], '[', 1)) %>% gsub('^>', '', .)
    res$strand = ifelse(res$qe-res$qs > 0, '+', '-')
    res
}

filterMum <- function(df, minl=1000, flanks=1e4){
    coord = df %>% filter(abs(re-rs)>minl) %>% group_by(qid, rid) %>%
```

```
        summarize(qsL=min(qs)-flanks, qeL=max(qe)+flanks, rs=median(rs)) %>%
        ungroup %>% arrange(desc(rs)) %>%
        mutate(qid=factor(qid, levels=unique(qid))) %>% select(-rs)
    merge(df, coord) %>% filter(qs>qsL, qe<qeL) %>%
        mutate(qid=factor(qid, levels=levels(coord$qid))) %>% select(-qsL, -qeL)
}

delta <- readDelta("Files for Figures/variation/cassava_tme7_phase1_unzips_p0_scaffolds.delta.filter")
delta_chr <- filter(delta, str_detect(rid, "Chr"), re-rs > 1e4)  #%>% arrange(rid, rs, qs) %>%
#rename(A = rid, B = qid, AStart = rs, AEnd =  re, BStart = qs, BEnd =  qe)

diagMum <- function(df){
    ## Find best qid order
    rid.o = df %>% group_by(qid, rid) %>% summarize(base=sum(abs(qe-qs)),
                                                    rs=weighted.mean(rs, abs(qe-qs))) %>%
        ungroup %>% arrange(desc(base)) %>% group_by(qid) %>% do(head(., 1)) %>%
        ungroup %>% arrange(desc(rid), desc(rs)) %>%
        mutate(qid=factor(qid, levels=unique(qid)))
    ## Find best qid strand
    major.strand = df %>% group_by(qid) %>%
        summarize(major.strand=ifelse(sum(sign(qe-qs)*abs(qe-qs))>0, '+', '-'),
                  maxQ=max(c(qe, qs)))
    merge(df, major.strand) %>% mutate(qs=ifelse(major.strand=='-', maxQ-qs, qs),
                                       qe=ifelse(major.strand=='-', maxQ-qe, qe),
                                       qid=factor(qid, levels=levels(rid.o$qid)))
}

delta_chr.diag <- diagMum(delta_chr) %>%
  mutate(rlab = paste0("Chr", str_extract(rid, "[0-9][0-9]"))) %>%
  mutate(similarity = 1 - error / abs(qe - qs)) %>%
  arrange(desc(as.numeric(qid)))

ctg_lengths <-
    delta_chr.diag %>% group_by(qid) %>% summarise(ctg_length = sum(abs(qe-qs))) %>%
    ungroup() %>%
    arrange(desc(as.numeric(qid))) %>%
    mutate(ypad = lag(ctg_length, default = 0),
           ycumsumpad = cumsum(ypad)
           )


chrm_lengths <-
    delta_chr.diag %>% group_by(rid) %>% summarise(length = max(re)) %>%
    ungroup() %>%
    mutate(xpad = lag(length, default = 0),
           xcumsumpad = cumsum(xpad),
           labelpos = xcumsumpad + length/2,
           label = paste0("Chr", str_extract(rid, "[0-9][0-9]")))

delta_chr.diag <- delta_chr.diag %>%
    left_join(chrm_lengths, by = "rid") %>%
    left_join(ctg_lengths, by = "qid")
```

```r
hapDotPlot <- delta_chr.diag %>%
  ggplot() +
  geom_point(
    data = filter(delta_chr.diag, similarity >= 0.98),
    aes(
      x = xcumsumpad + rs,
      y = ycumsumpad + qs,
      color = similarity,
      size = ctg_length
    ),
    alpha = 0.5
  ) +
  geom_point(
    data = filter(delta_chr.diag, between(similarity, 0.94, 0.98)),
    aes(
      x = xcumsumpad + rs,
      y = ycumsumpad + qs,
      color = similarity,
      size = ctg_length
    ),
    alpha = 0.5
  ) +
  geom_point(
    data = filter(delta_chr.diag, similarity <= 0.94),
    aes(
      x = xcumsumpad + rs,
      y = ycumsumpad + qs,
      color = similarity,
      size = ctg_length
    ),
    alpha = 0.5
  ) +
  geom_vline(data = chrm_lengths,
             aes(xintercept = xcumsumpad),
             linetype = 2) +
  geom_text(data = chrm_lengths, aes(x = labelpos, y = 1000, label = label)) +
  scale_size_continuous(name = "Haplotig\nlength") +
  labs(x = "Phase0 scaffolds", y = "Phase1 contigs") +
  scale_color_viridis_c() +
  cowplot::theme_cowplot()
```

```r
tme7_gff <-
    read_tsv(
        "Files for Figures/gffs/tme7_200703_falcon_phase0.gff",
        skip = 3,
        col_names = c(
            "seqid",
            "source",
            "type",
            "start",
            "end",
            "score",
            "strand",
            "phase",
```

```
            "attributes"
        ),
        comment = "#"
    )

tme7_TEs <-
    read_tsv(
        "Files for Figures/gffs/cassava_tme7_phase0_scaffolded_renamed.fasta.mod.EDTA.TEanno.gff3",
        skip = 3,
        col_names = c(
            "seqid",
            "source",
            "type",
            "start",
            "end",
            "score",
            "strand",
            "phase",
            "attributes"
        ),
        comment = "#"
    )

tme7_SVs <- SV_p1 %>% select(Chrm, SV_start, type) %>%
    rename(seqid = Chrm, start = SV_start) %>%
    mutate(seqid = str_remove(seqid, "omosome"),
           seqid = str_replace(seqid, "Phase", "P"))

genesTEs <-
    bind_rows(
        "Genes" = tme7_gff %>% filter(type == "gene"),
        "TE" = tme7_TEs,
        "SVs" = tme7_SVs,
        .id = "anno"
    )


annoDist <- genesTEs %>%
    filter(grepl("Chr", seqid)) %>%
    ggplot() +
    geom_density(aes(x = start, y = after_stat(ndensity), fill = anno, color = anno), alpha = 0.6) +
    facet_wrap(~ seqid, ncol = 6, scales = "free_x") +
    cowplot::theme_cowplot() +
    theme(axis.text.x = element_text(angle = 30, hjust = 1)) +
    cowplot::panel_border() +
    labs(x = "Genomic position (Mb)", y = "Normalized density") +
    shades::lightness(scale_color_manual(values = c(viridisLite::viridis(4)[-4]),
                                         name="Feature"), shades::scalefac(0.6)) +
    scale_fill_manual(values = c(viridisLite::viridis(4)[-4]),
                      name="Feature") +
    scale_x_continuous(labels=format_genomic())

pdf("fig5.pdf", width = 12, 12)
```

```r
cowplot::plot_grid(hapDotPlot, annoDist , ncol = 1, labels = "auto")
dev.off()
```

```
## pdf
##   2
```

```r
cowplot::plot_grid(hapDotPlot, annoDist , ncol = 1, labels = "auto")
```

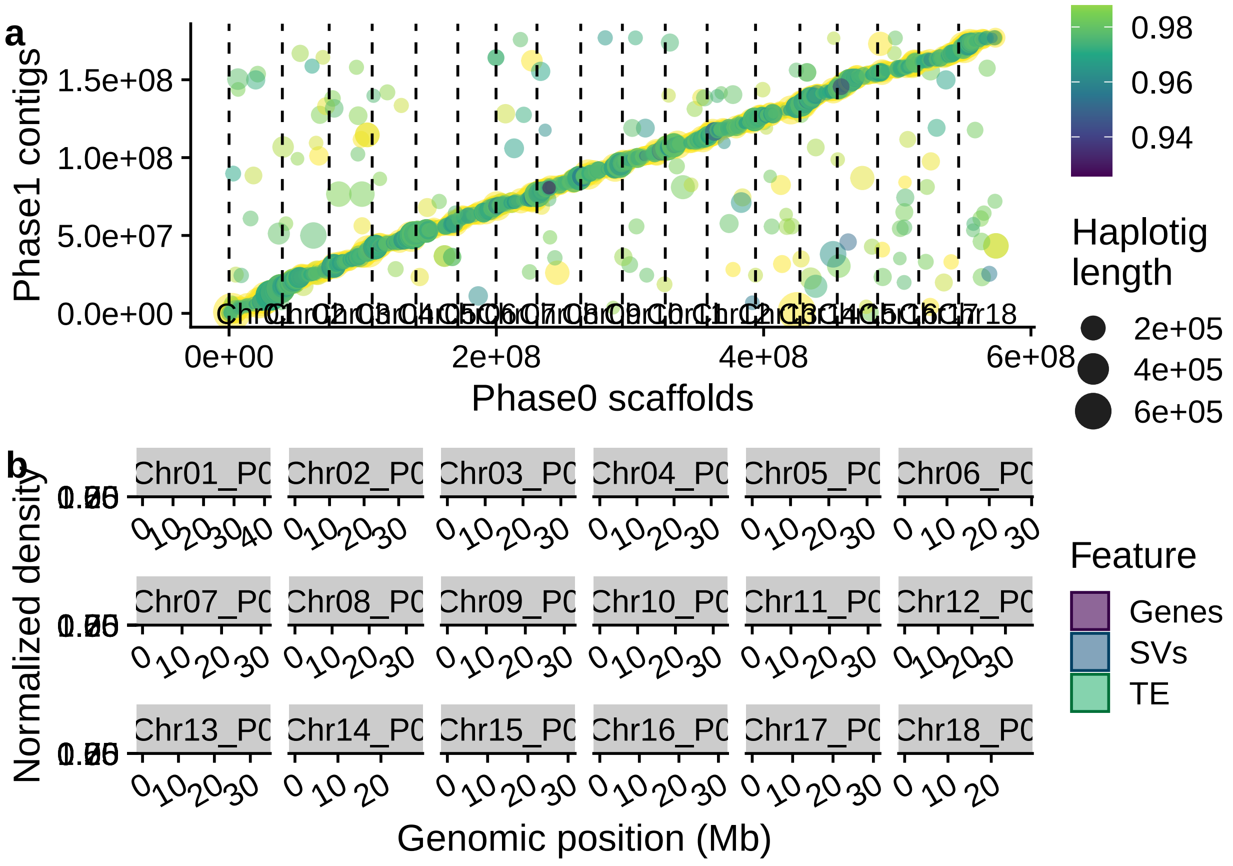

Figure 6

In python MCScanX

Figure 7

```r
SV_Ref <-
    read_tsv(file = "Files for Figures/variation/Cassava_Phase0_renamed_split10_Ns_vs_esculenta_305_v6_
    separate(reference, into = c("Chrm", "coords"), sep = ":") %>%
    separate(
        coords,
        into = c("ctg_start", "ctg_end"),
        sep = "-",
        convert = TRUE
    ) %>%
    mutate(
        SV_start = ctg_start + ref_start,
        SV_end = ctg_start + ref_stop,
```

```
        SV_cmd = paste0(
            "-o ",
            Chrm,
            "_",
            SV_start,
            "_",
            SV_end,
            " -c ",
            Chrm,
            " -s ",
            SV_start,
            " -e ",
            SV_end
        )
    ) %>%
    mutate(query_coordinates = ifelse(
        str_count(query_coordinates, ":") == 2,
        sub("Phase0:", "Phase0:0-0:",
            query_coordinates),
        query_coordinates
    )) %>%
    separate(
        query_coordinates,
        into = c(
            "query_Chrm",
            "query_ctg_pos",
            "query_int_pos",
            "query_strand"
        ),
        sep = ":"
    ) %>%
    separate(
        col = query_ctg_pos,
        into = c("query_ctg_start", "query_ctg_end"),
        sep = "-",
        convert = T
    ) %>%
    separate(
        col = query_int_pos,
        into = c("query_int_start", "query_int_end"),
        sep = "-",
        convert = T
    ) %>%
    mutate(
        query_SV_start = query_ctg_start + query_int_start,
        query_SV_end = query_ctg_start + query_int_end
    )


SV_Ref %>%
    filter(type == "Deletion") %>%
    arrange(desc(size)) %>% head
```

```
## # A tibble: 6 x 23
```

```
##   Chrm     ctg_start  ctg_end ref_start ref_stop ID           size strand type
##   <chr>        <int>    <int>     <dbl>    <dbl> <chr>       <dbl> <chr>  <chr>
## 1 Chromos~  11718858 11753535     23242    33116 Assemblyti~  9874 +      Delet~
## 2 Chromos~  19391747 19444245     18902    27886 Assemblyti~  8984 +      Delet~
## 3 Chromos~  26031509 26121103     50400    59066 Assemblyti~  8666 +      Delet~
## 4 Chromos~  22419269 22465670     32419    40418 Assemblyti~  7982 +      Delet~
## 5 Chromos~  14164795 14206982     23279    30937 Assemblyti~  7658 +      Delet~
## 6 Chromos~    543394   619976     54989    62492 Assemblyti~  7499 +      Delet~
## # ... with 14 more variables: ref_gap_size <dbl>, query_gap_size <dbl>,
## #   query_Chrm <chr>, query_ctg_start <int>, query_ctg_end <int>,
## #   query_int_start <int>, query_int_end <int>, query_strand <chr>,
## #   method <chr>, SV_start <dbl>, SV_end <dbl>, SV_cmd <chr>,
## #   query_SV_start <int>, query_SV_end <int>
```

```r
# SV_Ref %>% #filter(type != "Tandem_contraction") %>%
#     ggplot() +
#     geom_histogram(aes(x = size, fill = type), binwidth = 100) +
#     facet_wrap(~ str_replace(type, "_", " "),
#                ncol = 1, scales = "free_y") +
#     cowplot::theme_cowplot() +
#     theme(axis.text.x = element_text(angle = 30, hjust = 1)) +
#     cowplot::panel_border() +
#     labs(x = "Variant size (bp)", y = "Count") +
#     guides(fill = guide_none()) +
#     scale_x_log10()

write_delim(x = SV_Ref %>%  select(Chrm, SV_start, SV_end, everything(), -SV_cmd, -ctg_start, -ctg_end,

SV_Ref_dist_plot <- SV_Ref %>%
    mutate(bin = cut(size, breaks = c(0, 100, 500, 1000, 2500, 5000, 10000))) %>%
    group_by(type) %>%
    add_count(name = "total") %>%
    mutate(facet_label = paste0(str_replace(type, "_", " "), " (n=", total, ")")) %>%
    group_by(bin, facet_label) %>%
    count() %>%
    ggplot() +
    geom_bar(aes(x = bin, y = n, fill = facet_label), stat = "identity") +
    facet_wrap(~ facet_label,
               ncol = 1,
               scales = "free_y") +
    cowplot::theme_cowplot() +
    theme(axis.text.x = element_text(angle = 30, hjust = 1)) +
    cowplot::panel_border() +
    labs(x = "Variant size (bp)", y = "Count") +
    scale_x_discrete(labels = c("0-100", "100-500", "500-1000", "1000-2500", "2500-5000", "5000-10000")
    guides(fill = guide_none()) + scale_fill_viridis_d()


DELs <- SV_Ref %>% filter(type == "Deletion") %>%
    select(Chrm, SV_start, SV_end) %>%
    arrange(Chrm, SV_start) %>%
    rename()

#write_tsv(DELs, path = "dels.bed", col_names = FALSE)
```

```r
gff <- read_tsv("Files for Figures/variation/Mesculenta_305_v6.1.gene.gff3", skip = 3, col_names = c("s

gff %>%
    filter(type == "gene") %>%
    arrange(seqid, start) %>%
    write_tsv(path = "genes.gff3", col_names = FALSE)

gene_dist <- gff %>%
    filter(type == "gene") %>%
    group_by(seqid, strand) %>% arrange(seqid, start) %>%
    mutate(distanceUp = case_when(
      strand == "+" ~ start - lag(end),
      strand == "-" ~ lead(start) - end
    ))


# From bedtools closest -D b
closest <- read_tsv(file = "Files for Figures/variation/closest.bed", col_names = FALSE)
gene_closest <- read_tsv("Files for Figures/variation/AM560genes_vs_TME7dels.bed", col_names = FALSE)


closest <- closest %>%
    mutate(WhereDel = ifelse(X13 < 0, "Upstream", "Downstream"), #this is correct because i used closes
        # WhereDel = ifelse((X10 == "+" & X13 < 0) | (X10 == "-" & X13 > 0), "Upstream", "Downstream"),
          WhereDel = fct_relevel(WhereDel, "Upstream"))

SV_distance_plot <- closest %>% filter(X13 != 0) %>% filter(abs(X13) <= 1e4) %>%
    ggplot() +
    geom_density(aes(x = X13)) + facet_grid(~ WhereDel, scales = "free") +
    labs(x = "Distance to nearest gene") +
    cowplot::theme_cowplot() +
    theme(axis.text.x = element_text(angle = 30, hjust = 1)) +
    cowplot::panel_border()  +
    scale_x_continuous(breaks = c(seq(0, 25000, by = 2000), seq(0, -25000, by = -2000)))
```

Chr03 Het SV dotplot

```r
readDelta <- function(deltafile){
  lines = scan(deltafile, 'a', sep='\n', quiet=TRUE)
  lines = lines[-1]
  lines.l = strsplit(lines, ' ')
  lines.len = lapply(lines.l, length) %>% as.numeric
  lines.l = lines.l[lines.len != 1]
  lines.len = lines.len[lines.len != 1]
  head.pos = which(lines.len == 4)
  head.id = rep(head.pos, c(head.pos[-1], length(lines.l)+1)-head.pos)
  mat = matrix(as.numeric(unlist(lines.l[lines.len==7])), 7)
  res = as.data.frame(t(mat[1:5,]))
  colnames(res) = c('rs','re','qs','qe','error')
  res$qid = unlist(lapply(lines.l[head.id[lines.len==7]], '[', 2))
  res$rid = unlist(lapply(lines.l[head.id[lines.len==7]], '[', 1)) %>% gsub('^>', '', .)
  res$strand = ifelse(res$qe-res$qs > 0, '+', '-')
  res
```

```r
}

mumgp <- readDelta("Files for Figures/variation/cassava_tme7_phase1_unzips_p0_scaffolds.delta")

filterMum <- function(df, minl=1000, flanks=1e4){
    coord = df %>% filter(abs(re-rs)>minl) %>% group_by(qid, rid) %>%
        summarize(qsL=min(qs)-flanks, qeL=max(qe)+flanks, rs=median(rs)) %>%
        ungroup %>% arrange(desc(rs)) %>%
        mutate(qid=factor(qid, levels=unique(qid))) %>% select(-rs)
    merge(df, coord) %>% filter(qs>qsL, qe<qeL) %>%
        mutate(qid=factor(qid, levels=levels(coord$qid))) %>% select(-qsL, -qeL)
}

mumgp.filt = filterMum(mumgp, minl=1e5)

Chr03_mumgp <- mumgp %>% filter(rid == "Chromosome03_Phase0", qid == "001856F_006")

Chr03_mumgp_plot <- Chr03_mumgp %>%
    ggplot(aes(
        x = rs,
        xend = re,
        y = qs,
        yend = qe
    )) +
    geom_segment() +  cowplot::theme_cowplot() + geom_hline(yintercept = 64969, linetype = 3) +
    geom_hline(yintercept = 72186, linetype = 3) +
    xlab('Chromosome03_Phase0') +
    ylab('Haplotig 001856F_006') +
    xlim(17200000, 17237181) +
    scale_y_continuous(limits = c(62000, 90000),
                       breaks = c(60000, 64969, 70000, 72186, 80000, 90000))
```

```r
SV1 <- cowplot::ggdraw() +
  cowplot::draw_image(image = "Files for Figures/variation/Chromosome14_20000228_20004341.png")

SV2 <- cowplot::ggdraw() +
  cowplot::draw_image(image = "Files for Figures/variation/Chromosome03_14207739_14214954.png")

SV_right <- cowplot::plot_grid(SV1, SV2, ncol = 1, labels = c("c", "d"))

SV_top <- cowplot::plot_grid(SV_Ref_dist_plot, SV_right, ncol = 2, rel_widths = c(0.3, 1), labels = c("

SV_bot <- cowplot::plot_grid(SV_distance_plot, Chr03_mumgp_plot, rel_widths = c(6, 4), labels = c("b",

pdf("fig7.pdf", 14, 14)
cowplot::plot_grid(SV_top, SV_bot, ncol = 1, rel_heights = c(2.5, 1))
dev.off()
```

```
## pdf
##   2
```

```r
cowplot::plot_grid(SV_top, SV_bot, ncol = 1, rel_heights = c(2.5, 1))
```

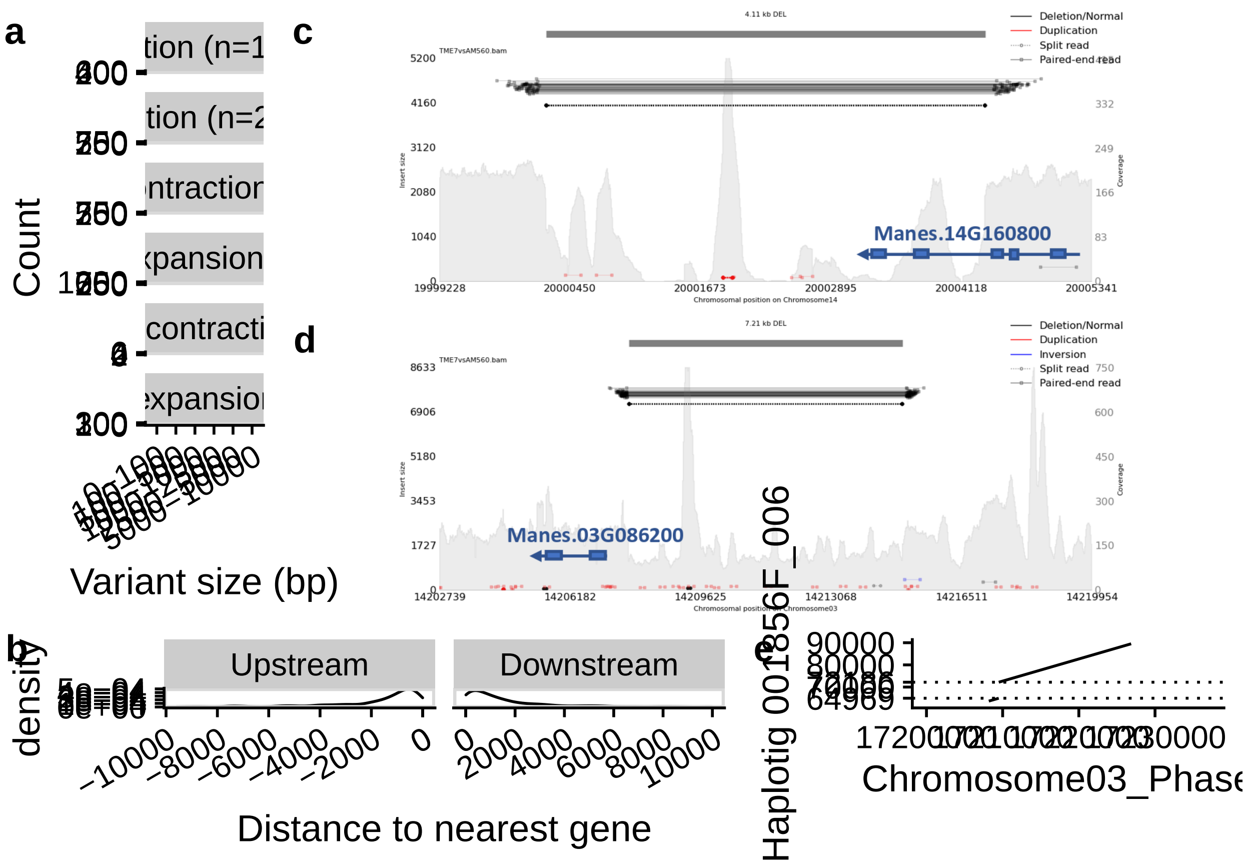

Figure 8

```
library(DESeq2)
library(viridis)

new_ase <- read_tsv("Files for Figures/variation/phaser_tissue_ase_all_samples.txt") %>%
    mutate(geneid = str_remove(str_extract(name, "(?<=ID=)(.*)(?=Name)"), ";")) %>%
    mutate(sample = str_remove(bam, "Aligned.sortedByCoord.out"))

new_ase_mat <- new_ase %>%
    select(geneid, aCount, bCount, sample) %>%
    pivot_wider(names_from = sample, values_from = c(aCount, bCount))

bams <- new_ase %>% pull(bam) %>% unique()

m <- 11

samples <- data.frame(count =  str_remove(colnames(new_ase_mat)[-1], "Count_.*"),
                      tissue = str_remove( str_remove(colnames(new_ase_mat)[-1], ".Count_"), "_[0-9]"),
                      rep =  str_remove(colnames(new_ase_mat)[-1], ".Count_.*_")
                      )

design <- ~ count*tissue

count_mat <- data.frame(new_ase_mat %>% dplyr::select(-geneid), row.names = new_ase_mat$geneid)
```

```r
library("DESeq2")
dds <- DESeqDataSetFromMatrix(count_mat, samples, design)
dds <- DESeq(dds)

keep <- rowSums(counts(dds)) >= 250

dds <- dds[keep, ]

resultsNames(dds)
```

```
##  [1] "Intercept"                   "count_b_vs_a"
##  [3] "tissue_fibrousroot_vs_fec" "tissue_lateralbud_vs_fec"
##  [5] "tissue_leaf_vs_fec"          "tissue_midvein_vs_fec"
##  [7] "tissue_oes_vs_fec"           "tissue_petiole_vs_fec"
##  [9] "tissue_ram_vs_fec"           "tissue_sam_vs_fec"
## [11] "tissue_stem_vs_fec"          "tissue_storageroot_vs_fec"
## [13] "countb.tissuefibrousroot"  "countb.tissuelateralbud"
## [15] "countb.tissueleaf"           "countb.tissuemidvein"
## [17] "countb.tissueoes"            "countb.tissuepetiole"
## [19] "countb.tissueram"            "countb.tissuesam"
## [21] "countb.tissuestem"           "countb.tissuestorageroot"
```

```r
ase_log2_mat <- as.data.frame(
    new_ase %>%
    filter(geneid %in% rownames(dds)) %>%
    mutate(aCount = aCount + 0.01,
           bCount = bCount + 0.01,
           newLog2_aFC = log2(aCount / bCount)
           ) %>%
    dplyr::select(geneid, newLog2_aFC, sample) %>%
    pivot_wider(names_from = sample, values_from = newLog2_aFC)
)

rownames(ase_log2_mat) <- ase_log2_mat$geneid
ase_log2_mat <- ase_log2_mat[-1]
```

PCAs

```r
# NON-ASE PCA
design <- ~ tissue

new_ase_mat2 <- new_ase %>%
   dplyr::select(geneid, totalCount, sample) %>%
    pivot_wider(names_from = sample, values_from = totalCount)

count_mat <- data.frame(new_ase_mat2 %>%dplyr::select(-geneid), row.names = new_ase_mat2$geneid)

dds2 <- DESeqDataSetFromMatrix(count_mat, samples[1:32, ], design)
dds2 <- DESeq(dds2)

keep <- rowSums(counts(dds2)) >= 250

dds2 <- dds2[keep, ]
```

```r
vst <- vst(dds2)

pca <- prcomp(t(assay(vst)))
percentVar <- pca$sdev^2 / sum( pca$sdev^2 )
d <- data.frame(PC1=pca$x[,1], PC2=pca$x[,2])
d$tissue <- str_remove(row.names(d), "_[0-9]")
pca_a <- d %>%
    ggplot(aes(x = PC1, y = PC2, color = tissue)) +
    geom_point() +
    ggrepel::geom_label_repel(aes(label = tissue), max.overlaps = 20) +
    xlab(paste0("PC1: ",round(percentVar[1] * 100),"% variance")) +
    ylab(paste0("PC2: ",round(percentVar[2] * 100),"% variance")) +
    # coord_fixed() +
    cowplot::theme_cowplot() +
    scale_color_viridis(discrete = T)

## ASE PCA
pca <- prcomp(t(ase_log2_mat))
percentVar <- pca$sdev^2 / sum( pca$sdev^2 )
d <- data.frame(PC1=pca$x[,1], PC2=pca$x[,2])
d$tissue <- str_remove(row.names(d), "_[0-9]")
pca_b <- d %>%
    ggplot(aes(x = PC1, y = PC2, color = tissue)) +
    geom_point() +
    ggrepel::geom_label_repel(aes(label = tissue), max.overlaps = 20) +
    xlab(paste0("PC1: ",round(percentVar[1] * 100),"% variance")) +
    ylab(paste0("PC2: ",round(percentVar[2] * 100),"% variance")) +
    # coord_fixed() +
    cowplot::theme_cowplot()  +
    scale_color_viridis(discrete = T)
```

ASE results

```r
# get Results tables
tissueCounts <- resultsNames(dds)[grep("count.", x = resultsNames(dds))]
ase_results <- lapply(tissueCounts, function(x)
{r <- results(dds, contrast=list( c("count_b_vs_a", x) ))
r %>% as_tibble() %>%
    mutate(geneid = rownames(r),
           contrast = x)}
) %>% bind_rows() %>%
    mutate(tissue = ifelse(contrast == "count_b_vs_a",
                           "fec",
                           str_remove(contrast, "countb.tissue"))) %>%
    filter(!is.na(padj)) %>%
    mutate(
        ASE_type = case_when(padj > 0.05 ~ "No ASE",
                             abs(log2FoldChange) < 5 & padj < 0.05 ~ "Partial ASE",
                             abs(log2FoldChange) >= 5 & padj < 0.05 ~ "Complete ASE")) %>%
    mutate(ASE_type = fct_relevel(ASE_type, "No ASE", "Partial ASE", "Complete ASE")) %>%
    filter(!is.na(padj)) %>%
    left_join(new_ase %>%
                  dplyr::select(contig, start, stop, geneid) %>%
```

```
                  distinct(),
              by = "geneid")

ase_genes <- ase_results %>%
    group_by(contrast) %>%
    group_split() %>%
    lapply(X = ., function(x) {x %>%
            filter(ASE_type != "No ASE") %>%
            pull(geneid)}
           )

names(ase_genes) <- c("fec", str_remove(tissueCounts, "countb.tissue")[-1])

library(UpSetR)
upset(fromList(ase_genes), order.by = "freq", nsets = 11, )
```

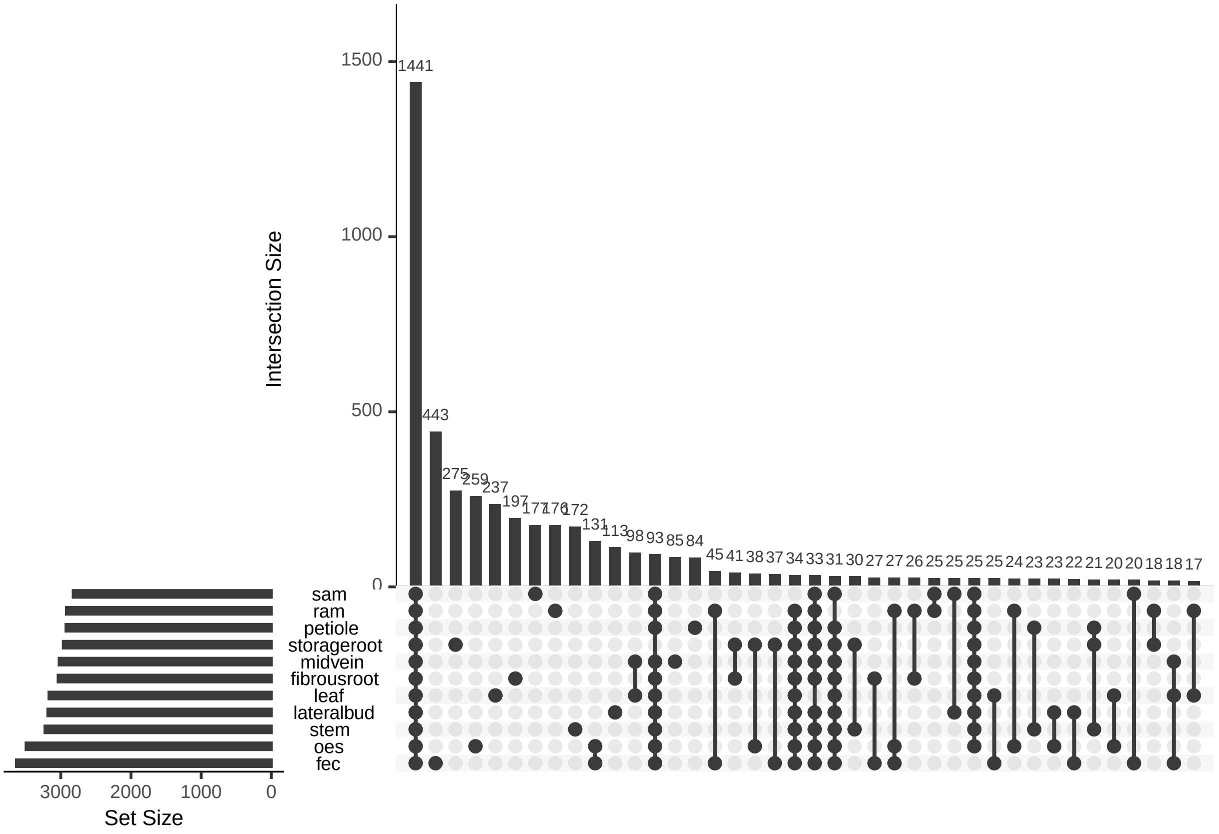


```
length(Reduce(union, ase_genes))
```

```
## [1] 7222
```

```
complete_ase_genes <- ase_results %>%
    group_by(contrast) %>%
    group_split() %>%
    lapply(X = ., function(x) {x %>%
            filter(ASE_type == "Complete ASE") %>%
            pull(geneid)}
    )
```

```r
names(complete_ase_genes) <- c("fec", str_remove(tissueCounts, "countb.tissue")[-1])
length(Reduce(union, complete_ase_genes))
```

```
## [1] 1800
```

```r
# barplot
bar <- ase_results %>%
    group_by(tissue) %>%
    mutate(readsDetected = n()) %>%
    group_by(tissue, ASE_type) %>%
    mutate(Number = n(),
           label = paste0(round(Number / readsDetected * 100, digits = 2), "%")) %>%
    summarise(Number = unique(Number), label = unique(label)) %>%
    mutate(ypos = case_when(ASE_type == "Complete ASE" ~ as.numeric(Number / 2),
                            ASE_type == "No ASE" ~ 10000,
                            TRUE ~ 3500)) %>%
    ggplot(aes(x = tissue, y = Number, fill = ASE_type, label = label)) +
    geom_bar(stat = "identity", position = "stack") +
    geom_text(# data = . %>%  filter(ASE_type != "Complete ASE"),
              stat = "identity", position = position_stack(vjust = 0.5), color = "white") +
    # ggrepel::geom_text_repel(data = . %>%  filter(ASE_type == "Complete ASE"),
    #                          aes(y = ypos),
    #                          color = "white",
    #                          nudge_y = 750,
    #                          stat = "identity",
    #                          direction = "y", arrow = arrow(length = unit(0.015, "npc")),
    #                                                          min.segment.length = unit(0, 'lines')) +
    scale_fill_manual(values = c(viridisLite::viridis(4, direction = -1))[-1],
                      name="ASE type") +
    cowplot::theme_cowplot() +
    cowplot::panel_border() +
    theme(axis.text.x = element_text(angle = 35, vjust = 0.5))

ma <- ase_results %>%
    # arrange(factor(ASE_type2, levels = c("Partial ASE", "Complete ASE", "No ASE"))) %>%
    ggplot(aes(x = log10(baseMean), y = log2FoldChange, color = ASE_type), alpha = 0.2) +
    geom_point() +
    geom_point(data = . %>% filter(log2FoldChange < -15), aes(y = -14.5), shape = 25) +
    geom_point(data = . %>% filter(log2FoldChange > 15), aes(y = 14.5), shape = 24) +
    ylim(-15, 15) +
    facet_grid(~tissue) +
    scale_color_manual(values = c(viridisLite::viridis(4, direction = -1))[-1],
                       name="ASE type") +
    cowplot::theme_cowplot() +
    cowplot::panel_border()

pca_a
```

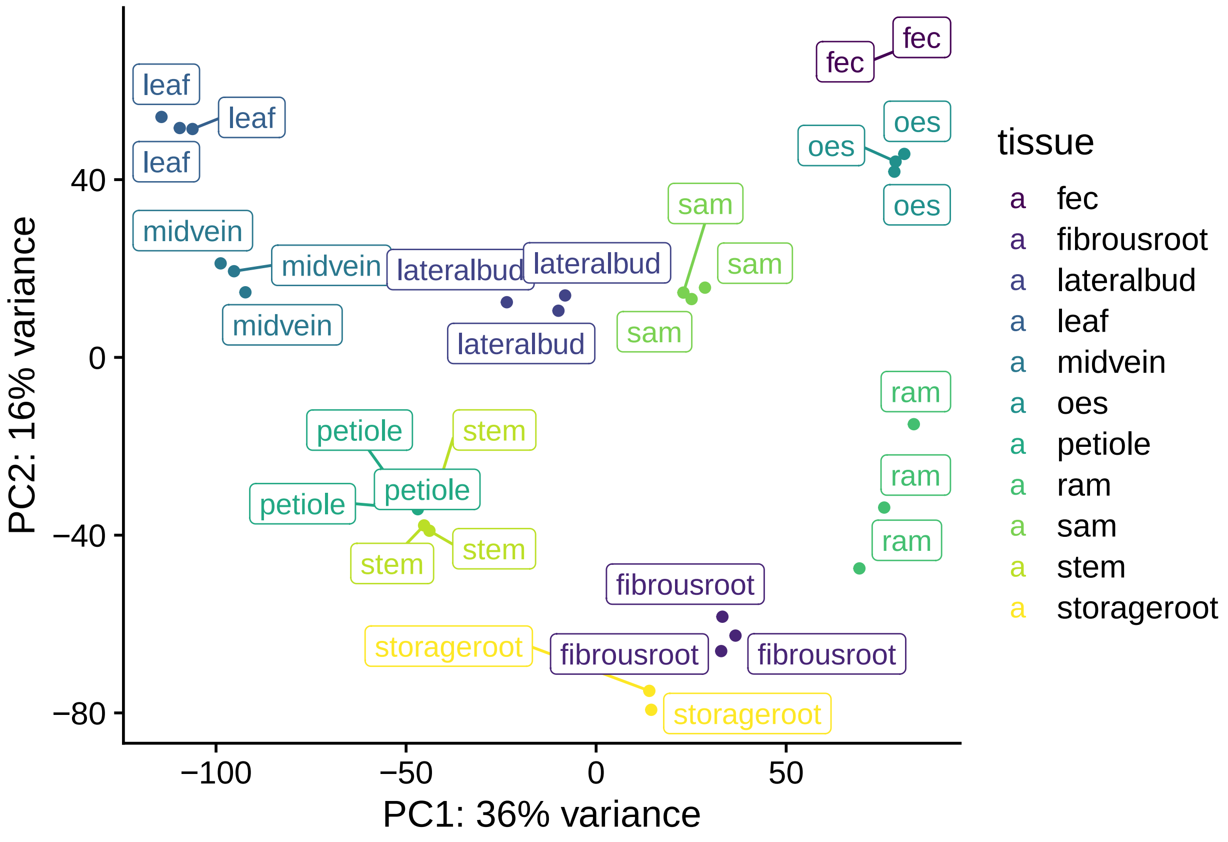

tissue
fec
fibrousroot
lateralbud
leaf
midvein
oes
petiole
ram
sam
stem
storageroot
PC1: 36% variance
PC2: 16% variance


```
pca_b
```

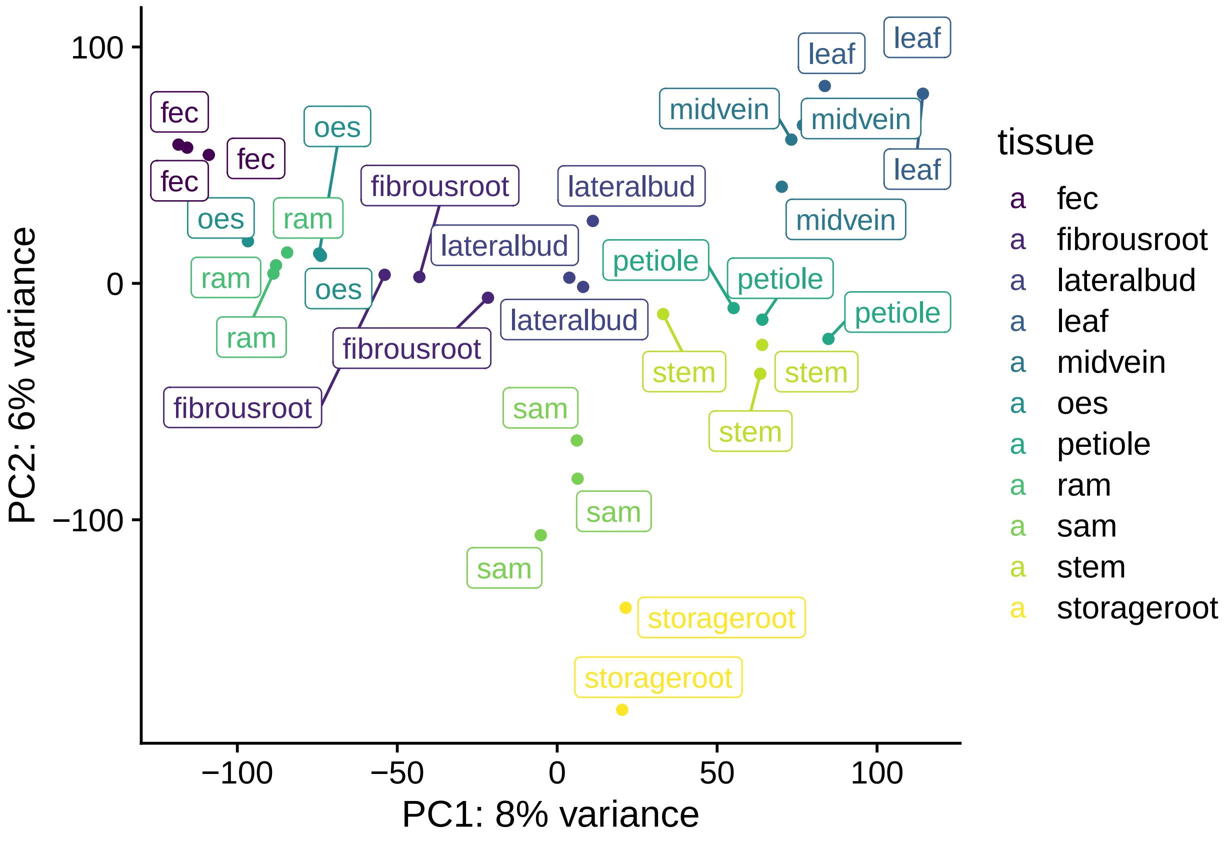
PC1: 8% variance
PC2: 6% variance
tissue
fec
fibrousroot
lateralbud
leaf
midvein
oes
petiole
ram
sam
stem
storageroot

```
bar
```

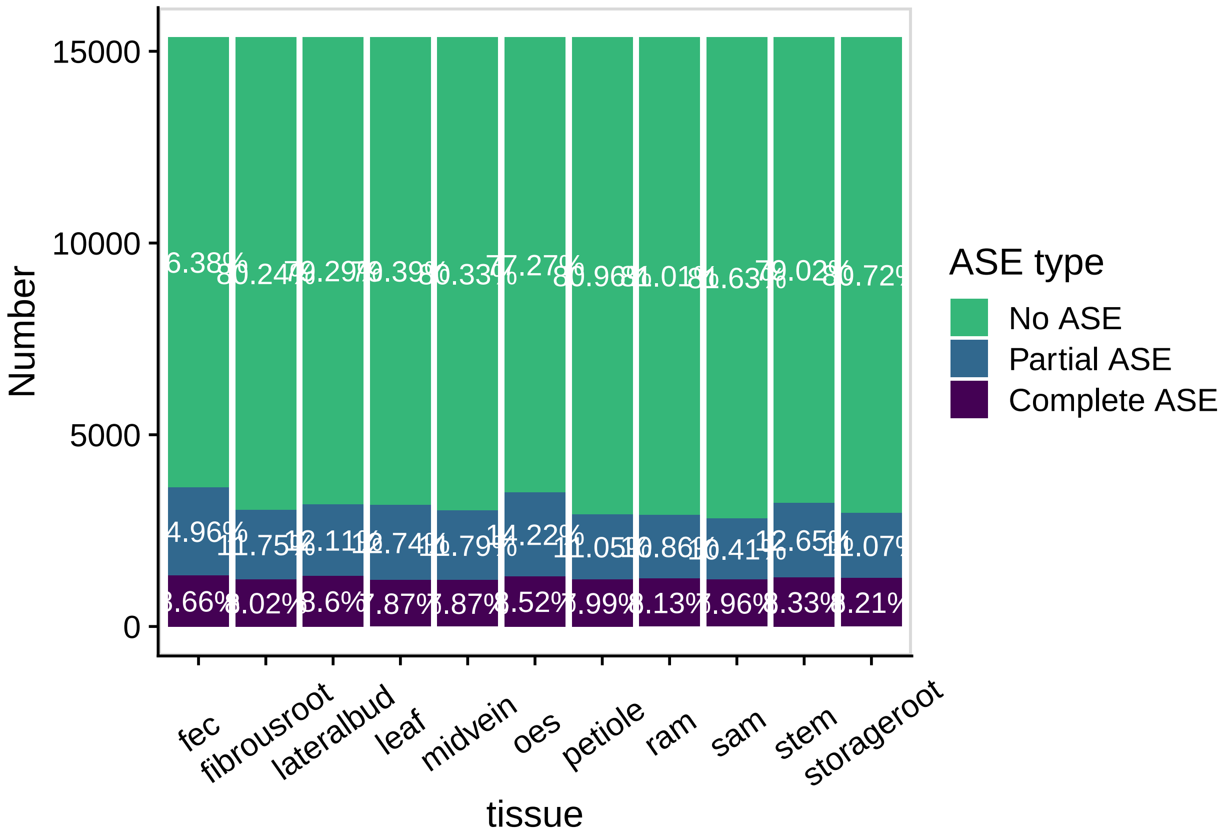
Number
15000
10000
5000
0
tissue
fec
fibrousroot
lateralbud
leaf
midvein
oes
petiole
ram
sam
stem
storageroot
ASE type
No ASE
Partial ASE
Complete ASE

```
ma
```

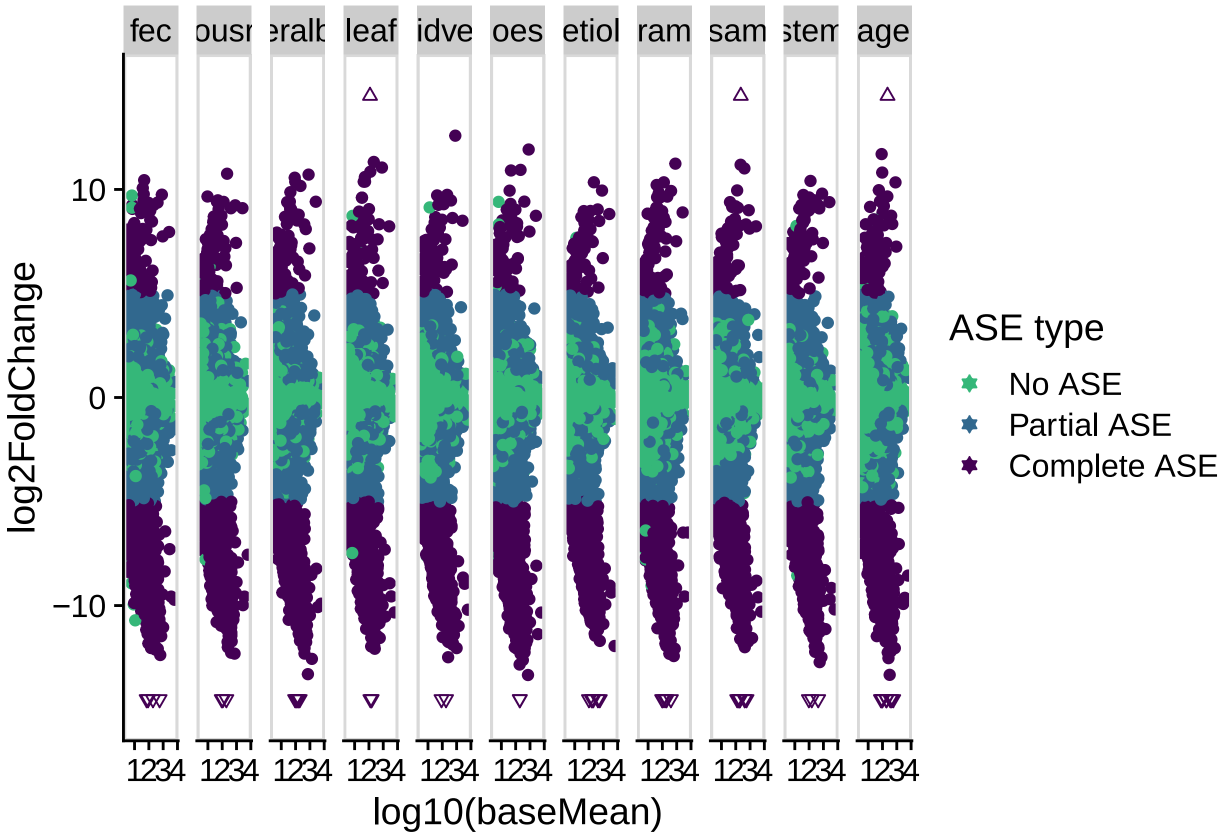

ASE GO terms

```
### GO terms

library(topGO)
cassavaGO <- read_csv(file = "Files for Figures/GO terms/cassavaGO_Manual.csv", col_names = TRUE)

#prepare as named list for use with topGO
GOList <- setNames(nm = cassavaGO$cassava_gene, strsplit(cassavaGO$allGOterms.dedup, "; "))

runTopGoAnalysis <- function(geneSet, allgenes, GOList, nodeSize){

    alg <- factor(as.integer(allgenes %in% geneSet))
    names(alg) <- allgenes

tgd <-
    new(
        "topGOdata",
        ontology = "BP",
        allGenes = alg,
        nodeSize = 100,
        annot = topGO::annFUN.gene2GO,
        gene2GO = GOList
    )
tgd
```

```r
}

exportGOtable <- function(tgd_object,
             orderTest = "Fisher.weight01",
             n = 25,
             joinfun = "intersect") {
        resultTopGO.weight01 <-
            topGO::runTest(tgd_object, algorithm = "weight01", statistic = "Fisher")
        topGO::GenTable(
            tgd_object,
            #Fisher.elim = resultTopGO.elim,
            #Fisher.classic = resultTopGO.classic,
            Fisher.weight01 = resultTopGO.weight01,
            #Fisher.pc = resultTopGO.parentchild,
            orderBy = orderTest,
            topNodes = min(resultTopGO.weight01@geneData[4], n)
        )
    }


tgdToTable <- function(tgd, n = 500) {
    GO <- exportGOtable(tgd, "Fisher.weight01", n = n) %>%
        mutate(Fisher.weight01 = ifelse(Fisher.weight01 == "< 1e-30", "1e-30", Fisher.weight01)) %>%
        mutate(Fisher.weight01 = as.numeric(Fisher.weight01))

    # subset(GO, Fisher.weight01 < cutoff)
    GO
}


#union
tgd <- runTopGoAnalysis(geneSet = Reduce(union, ase_genes),
                        allgenes = rownames(dds),
                        GOList = GOList,
                        nodeSize = 100)

union_genes <- tgdToTable(tgd, 50) %>% filter(Fisher.weight01 < 0.05) #%>% dplyr::select(GO.ID, Term, F

union_genes
```

```
##         GO.ID                                        Term Annotated Significant
## 1  GO:1901565 organonitrogen compound catabolic proces...       585         315
## 2  GO:1901566 organonitrogen compound biosynthetic pro...      1096         591
## 3  GO:1901607       alpha-amino acid biosynthetic process       131          82
## 4  GO:0044282            small molecule catabolic process       197         120
## 5  GO:0044270 cellular nitrogen compound catabolic pro...       148          81
## 6  GO:0045935 positive regulation of nucleobase-contai...       314         124
## 7  GO:0010604 positive regulation of macromolecule met...       458         190
## 8  GO:0046700               heterocycle catabolic process       145          78
## 9  GO:0055086 nucleobase-containing small molecule met...       301         175
## 10 GO:0006091 generation of precursor metabolites and ...       267         150
## 11 GO:0006518                   peptide metabolic process       518         279
## 12 GO:1901362 organic cyclic compound biosynthetic pro...      1802         764
## 13 GO:0018130            heterocycle biosynthetic process      1670         694
```

```
## 14 GO:0010035            response to inorganic substance       614         318
## 15 GO:0019438      aromatic compound biosynthetic process      1725         722
## 16 GO:0044271 cellular nitrogen compound biosynthetic ...      2118         924
## 17 GO:0042742              defense response to bacterium       276         151
## 18 GO:0044283         small molecule biosynthetic process       503         266
## 19 GO:1901361 organic cyclic compound catabolic proces...       175          93
## 20 GO:0055085                    transmembrane transport       669         344
## 21 GO:1901615 organic hydroxy compound metabolic proce...       261         139
## 22 GO:0010033              response to organic substance      1120         538
## 23 GO:0019439         aromatic compound catabolic process       159          84
## 24 GO:0045229 external encapsulating structure organiz...       152          75
## 25 GO:0072594 establishment of protein localization to...       219         121
## 26 GO:0006412                                 translation       443         233
## 27 GO:0071702                 organic substance transport       788         408
## 28 GO:0055114                oxidation-reduction process       919         466
## 29 GO:0017038                              protein import       100          59
## 30 GO:0009814 defense response, incompatible interacti...       118          68
## 31 GO:0009150    purine ribonucleotide metabolic process       159          89
## 32 GO:0046395           carboxylic acid catabolic process       130          74
## 33 GO:0016051           carbohydrate biosynthetic process       219         106
## 34 GO:0006464      cellular protein modification process      1938         896
## 35 GO:0006605                           protein targeting       202         110
## 36 GO:0015979                              photosynthesis       151          84
## 37 GO:0006796 phosphate-containing compound metabolic ...      1545         761
## 38 GO:0051247 positive regulation of protein metabolic...       123          56
## 39 GO:1901605          alpha-amino acid metabolic process       223         135
## 40 GO:0044281            small molecule metabolic process      1183         648
## 41 GO:0016310                             phosphorylation      1015         485
## 42 GO:0022603 regulation of anatomical structure morph...       102          58
## 43 GO:1901575         organic substance catabolic process       954         501
## 44 GO:0009165             nucleotide biosynthetic process       128          74
## 45 GO:0032787      monocarboxylic acid metabolic process       372         183
## 46 GO:0098662   inorganic cation transmembrane transport       162          88
## 47 GO:0046148              pigment biosynthetic process       105          59
## 48 GO:0008654           phospholipid biosynthetic process       109          61
## 49 GO:0044248                   cellular catabolic process       864         457
## 50 GO:0048583          regulation of response to stimulus       506         245
##    Expected Fisher.weight01
## 1    274.69        0.000048
## 2    514.64        0.000130
## 3     61.51        0.000210
## 4     92.50        0.000250
## 5     69.49        0.000290
## 6    147.44        0.000890
## 7    215.06        0.000900
## 8     68.09        0.001130
## 9    141.34        0.001170
## 10   125.37        0.001400
## 11   243.23        0.002120
## 12   846.15        0.002430
## 13   784.16        0.003050
## 14   288.31        0.003370
## 15   809.99        0.004990
## 16   994.53        0.005120
```

```
## 17   129.60        0.005430
## 18   236.19        0.005630
## 19    82.17        0.005630
## 20   314.14        0.005750
## 21   122.56        0.005900
## 22   525.91        0.006060
## 23    74.66        0.006670
## 24    71.37        0.007720
## 25   102.83        0.007950
## 26   208.01        0.008860
## 27   370.01        0.009660
## 28   431.53        0.009870
## 29    46.96        0.010120
## 30    55.41        0.012550
## 31    74.66        0.013500
## 32    61.04        0.013930
## 33   102.83        0.016450
## 34   910.01        0.017670
## 35    94.85        0.018750
## 36    70.90        0.019470
## 37   725.47        0.021070
## 38    57.76        0.022890
## 39   104.71        0.023780
## 40   555.49        0.025110
## 41   476.60        0.026470
## 42    47.90        0.027920
## 43   447.96        0.028130
## 44    60.10        0.030530
## 45   174.68        0.033940
## 46    76.07        0.035160
## 47    49.30        0.035570
## 48    51.18        0.036330
## 49   405.70        0.039480
## 50   237.60        0.039930
```

```
write_delim(union_genes, delim = "\t", file = "ASEgenes_goterms.tsv ")

#intersect
tgd <- runTopGoAnalysis(geneSet = Reduce(intersect, ase_genes),
                        allgenes = rownames(dds),
                        GOList = GOList,
                        nodeSize = 100)

tgdToTable(tgd, 50) %>% filter(Fisher.weight01 < 0.05) #%>% dplyr::select(GO.ID, Term, Fisher.weight01)
```

```
##         GO.ID                                        Term Annotated Significant
## 1  GO:0006412                                 translation       443          94
## 2  GO:0000398              mRNA splicing, via spliceosome       173          36
## 3  GO:0006457                             protein folding       144          30
## 4  GO:0017038                              protein import       100          23
## 5  GO:0072594 establishment of protein localization to...       219          39
## 6  GO:0016310                             phosphorylation      1015          89
## 7  GO:0022607                cellular component assembly       646         100
## 8  GO:0006605                           protein targeting       202          35
## 9  GO:0048193                      Golgi vesicle transport       152          28
```

```
## 10 GO:0051252         regulation of RNA metabolic process      1392         113
## 11 GO:0045229 external encapsulating structure organiz...       152          18
## 12 GO:0042325              regulation of phosphorylation       130          15
## 13 GO:0006364                            rRNA processing       193          33
## 14 GO:0022618          ribonucleoprotein complex assembly       121          23
## 15 GO:0046395           carboxylic acid catabolic process       130          24
## 16 GO:0016051           carbohydrate biosynthetic process       219          29
## 17 GO:0070925                         organelle assembly       143          25
## 18 GO:0072657            protein localization to membrane       107          20
## 19 GO:0046686                    response to cadmium ion       239          37
## 20 GO:0043161 proteasome-mediated ubiquitin-dependent ...       178          29
## 21 GO:0006520       cellular amino acid metabolic process       312          52
## 22 GO:0061024                     membrane organization       199          31
## 23 GO:0046907                    intracellular transport       605          96
## 24 GO:0006886            intracellular protein transport       464          73
## 25 GO:0048646 anatomical structure formation involved ...       138          11
## 26 GO:1901565 organonitrogen compound catabolic proces...       585          89
## 27 GO:0018193             peptidyl-amino acid modification       272          36
## 28 GO:0010608 posttranscriptional regulation of gene e...       175          27
## 29 GO:0009150     purine ribonucleotide metabolic process       159          25
## 30 GO:0007005                 mitochondrion organization       136          22
## 31 GO:0040007                                     growth       355          42
## 32 GO:0016458                             gene silencing       148          24
## 33 GO:0008380                               RNA splicing       219          46
## 34 GO:1901605          alpha-amino acid metabolic process       223          34
## 35 GO:0032787       monocarboxylic acid metabolic process       372          49
## 36 GO:0006091 generation of precursor metabolites and ...       267          37
## 37 GO:0097435           supramolecular fiber organization       126          20
## 38 GO:0019941 modification-dependent protein catabolic...       316          48
## 39 GO:0044419 interspecies interaction between organis...       688          66
## 40 GO:0000904 cell morphogenesis involved in different...       127          20
## 41 GO:0044282             small molecule catabolic process       197          36
## 42 GO:0044281             small molecule metabolic process      1183         160
## 43 GO:0006996                     organelle organization      1292         165
## 44 GO:0016052               carbohydrate catabolic process       158          23
## 45 GO:0048583          regulation of response to stimulus       506          54
## 46 GO:0006325                      chromatin organization       264          37
## 47 GO:0009408                           response to heat       176          25
## 48 GO:0006281                                 DNA repair       262          35
## 49 GO:0034655 nucleobase-containing compound catabolic...       103          16
## 50 GO:0006464       cellular protein modification process      1938         183
##    Expected Fisher.weight01
## 1     44.10       5.500e-13
## 2     17.22       1.400e-05
## 3     14.33       6.900e-05
## 4      9.95       9.900e-05
## 5     21.80       2.300e-04
## 6    101.04       3.800e-04
## 7     64.30       6.400e-04
## 8     20.11       7.800e-04
## 9     15.13       9.700e-04
## 10   138.56       1.060e-03
## 11    15.13       1.140e-03
## 12    12.94       1.250e-03
```

```
## 13    19.21       1.380e-03
## 14    12.04       1.730e-03
## 15    12.94       2.100e-03
## 16    21.80       2.640e-03
## 17    14.23       3.680e-03
## 18    10.65       4.090e-03
## 19    23.79       4.380e-03
## 20    17.72       5.320e-03
## 21    31.06       7.470e-03
## 22    19.81       7.860e-03
## 23    60.22       9.240e-03
## 24    46.19       9.400e-03
## 25    13.74       1.108e-02
## 26    58.23       1.133e-02
## 27    27.08       1.297e-02
## 28    17.42       1.411e-02
## 29    15.83       1.418e-02
## 30    13.54       1.509e-02
## 31    35.34       1.547e-02
## 32    14.73       1.763e-02
## 33    21.80       1.768e-02
## 34    22.20       1.806e-02
## 35    37.03       1.992e-02
## 36    26.58       2.391e-02
## 37    12.54       2.394e-02
## 38    31.46       2.454e-02
## 39    68.49       2.556e-02
## 40    12.64       2.585e-02
## 41    19.61       2.948e-02
## 42   117.76       3.391e-02
## 43   128.61       3.695e-02
## 44    15.73       4.036e-02
## 45    50.37       4.062e-02
## 46    26.28       4.281e-02
## 47    17.52       4.342e-02
## 48    26.08       4.375e-02
## 49    10.25       4.785e-02
## 50   192.91       4.920e-02
```

```r
# get distance info
dist_ase_up <- read_tsv(file = "Files for Figures/variation/closest_io_id_Da.txt", col_names = c("Chrm"

dist_ase_dwn <- read_tsv(file = "Files for Figures/variation/closest_io_iu_Da.txt", col_names = c("Chrm

dist_ase <- bind_rows("Upstream" = dist_ase_up, "Downstream" = dist_ase_dwn, .id = "stream")


ase_results_dist <- ase_results %>%
    left_join(dist_ase, by = c(
        "contig" = "Chrm",
        "start" = "start",
        "stop" = "end"
    )) %>%
    mutate(stream = fct_relevel(stream, "Upstream"))
```

```r
# Complete ASE across all tissue types

dist_plot <- ase_results_dist %>%
    group_by(stream, ASE_type) %>%
    filter(indel_chrm != ".", abs(dist) <= 20000) %>%
    distinct(geneid, .keep_all = T) %>%
    ggplot() +
    geom_density(aes(x = (dist), fill = geneid %in% Reduce(intersect, complete_ase_genes)), alpha = 0.6
    cowplot::theme_cowplot() +
    labs(x = "Distance to nearest large InDel") +
    scale_fill_manual(values = c(viridisLite::viridis(4, direction = -1))[-1],
                      name="ASE in\nall tissue") +
    facet_grid( ~ stream, scales = "free")

ase_results_dist %>%
    group_by(stream, ASE_type) %>%
    filter(indel_chrm != ".", abs(dist) <= 1e6) %>%
    distinct(geneid, .keep_all = T) %>%
    ggplot() +
    geom_density(aes(x = (dist), fill = geneid %in% Reduce(intersect, complete_ase_genes)), alpha = 0.6
    cowplot::theme_cowplot() +
    labs(x = "Distance to nearest large InDel") +
    scale_fill_manual(values = c(viridisLite::viridis(4, direction = -1))[-1],
                      name="ASE in\nall tissue") +
    facet_grid( ~ stream, scales = "free")
```

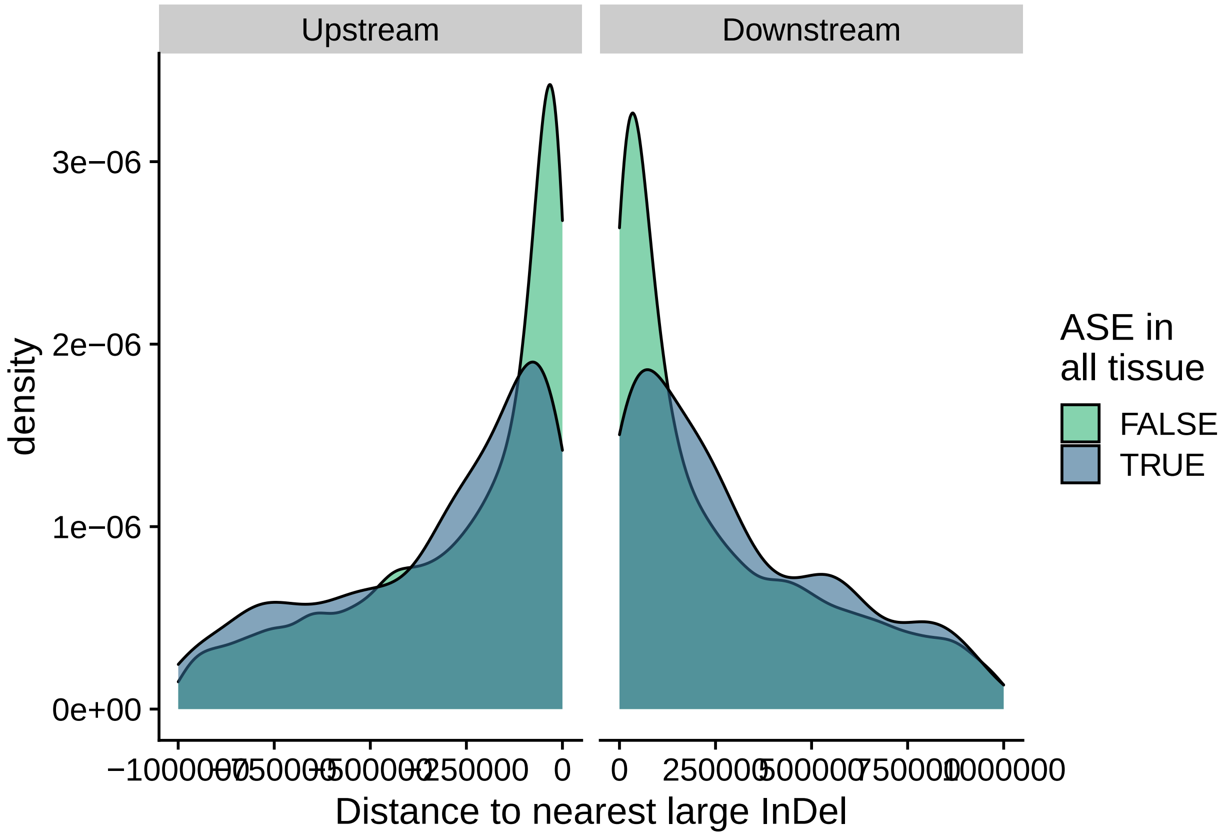

```r
library(writexl)

list_of_ase_tissues <- ase_results_dist %>% group_by(tissue) %>% group_split(tissue)

names(list_of_ase_tissues) <- unique(ase_results_dist$tissue)


write_xlsx(list_of_ase_tissues, 'Supplementary_file_3_ASE_and_SVs.xlsx')
```

```r
## PLOTS ###


ase_pcas <- cowplot::plot_grid(pca_a + theme(legend.position = "none",
                                 plot.title = element_text(hjust = 0.5)) +
                     labs(title = "Total count PCA"),
                   pca_b + theme(legend.position = "none",
                                 plot.title = element_text(hjust = 0.5)) +
                     labs(title = "Allele specific PCA"),
                   align = "h")

ase_dist_plots <- cowplot::plot_grid(bar, dist_plot, align = "h", axis = "b",
                                     labels = c("c", "d"))

cowplot::plot_grid(ase_pcas,
                   ma,
                   ase_dist_plots,
                   rel_heights = c(1, 0.7, 1),
                   labels = c("a", "b"),
                   align = "h", axis = "l",
                   ncol = 1)
```

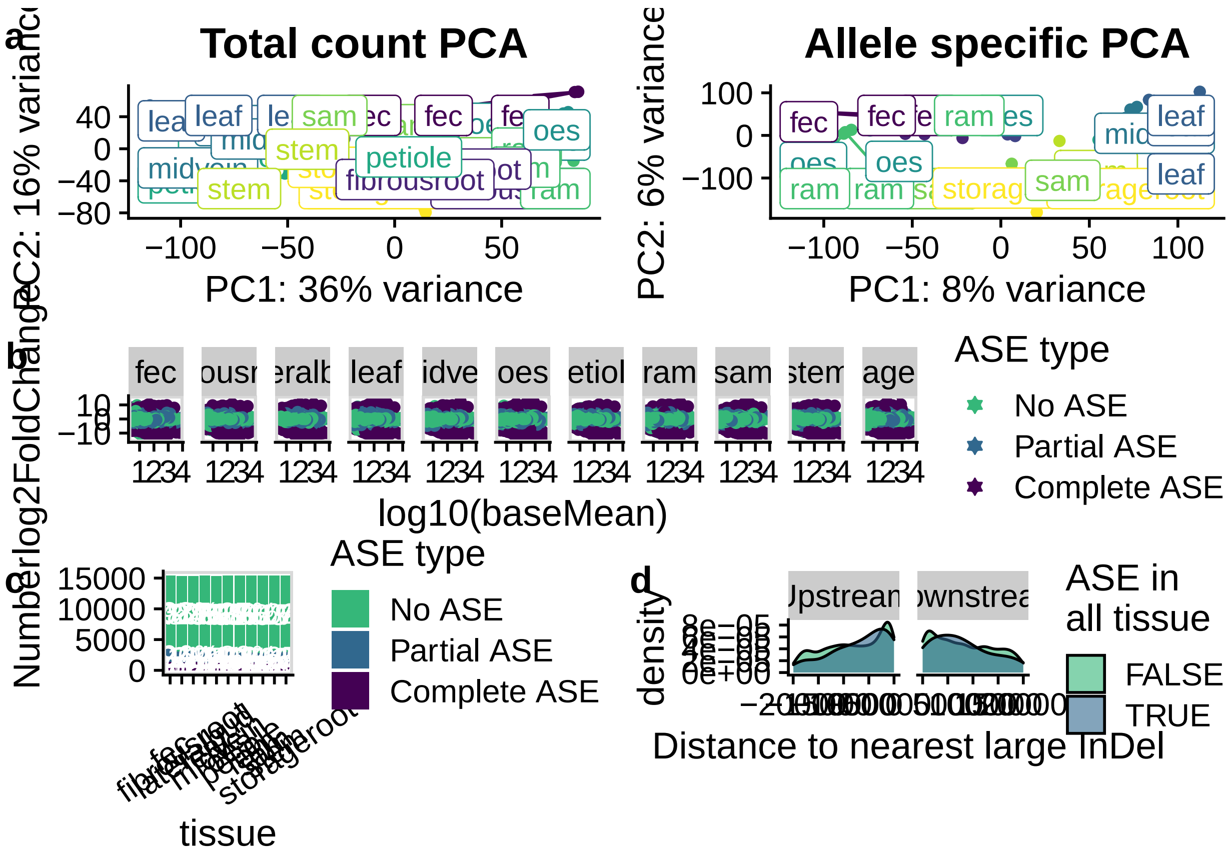

Tandem gene duplication analaysis

```
# The phase#.tandem files generated by running MCScanX on each phase's gff and protein.fasta separately

tandem0 <- read_delim(file = "Files for Figures/tandemdup/phase0.tandem", delim = ",", col_names = c("g
    mutate(array = gene2 == lead(gene1)) %>% # checks if the next gene1 is the same as gene2 (ie is arr
    mutate(array2 = ifelse(lag(array), TRUE, array), # marks the last pair in the array as array
           array2 = ifelse(is.na(array2), FALSE, array2)) %>% # corrects the first NA because of lead()
    mutate(yy = ifelse(array2 == TRUE, {yy = rle(array2); rep((yy$lengths), yy$lengths)}, 1))  #uses rl

tandem0 %>%
    group_by(yy) %>%
    tally() %>%
    mutate(count = n / yy)
```

```
## # A tibble: 10 x 3
##       yy     n count
##    <dbl> <int> <dbl>
##  1     1  1094  1094
##  2     2   180    90
##  3     3   108    36
##  4     4    72    18
##  5     5    20     4
##  6     6    36     6
##  7     7    21     3
##  8     8    24     3
```

```
## 9      9     9     1
## 10    10    10     1
```

```
tandem1 <- read_delim(file = "Files for Figures/tandemdup/phase1.tandem", delim = ",", col_names = c("g
    mutate(array = gene2 == lead(gene1)) %>% # checks if the next gene1 is the same as gene2 (ie is arr
    mutate(array2 = ifelse(lag(array), TRUE, array), # marks the last pair in the array as array
           array2 = ifelse(is.na(array2), FALSE, array2)) %>% # corrects the first NA because of lead()
    mutate(yy = ifelse(array2 == TRUE, {yy = rle(array2); rep((yy$lengths), yy$lengths)}, 1))  #uses rl

tandem1 %>%
    group_by(yy) %>%
    tally() %>%
    mutate(count = n / yy)
```

```
## # A tibble: 7 x 3
##      yy     n count
##   <dbl> <int> <dbl>
## 1     1   998   998
## 2     2   198    99
## 3     3   105    35
## 4     4    60    15
## 5     5    35     7
## 6     6    24     4
## 7     7     7     1
```

```
array_count <- bind_rows("phase0" = tandem0 %>%
                             group_by(yy) %>%
                             tally() %>%
                             mutate(count = n / yy),
                         "phase1" = tandem1 %>%
                             group_by(yy) %>%
                             tally() %>%
                             mutate(count = n / yy),
                         .id = "Phase"
)

array_count %>% mutate(`Array size` = yy + 1) %>%
    ggplot() +
    geom_bar(aes(x = `Array size`, y = count, fill = Phase), stat="identity", position = "dodge") +
    scale_x_continuous(breaks = 1:11) +
    scale_y_continuous(name = "Number of arrays")
```

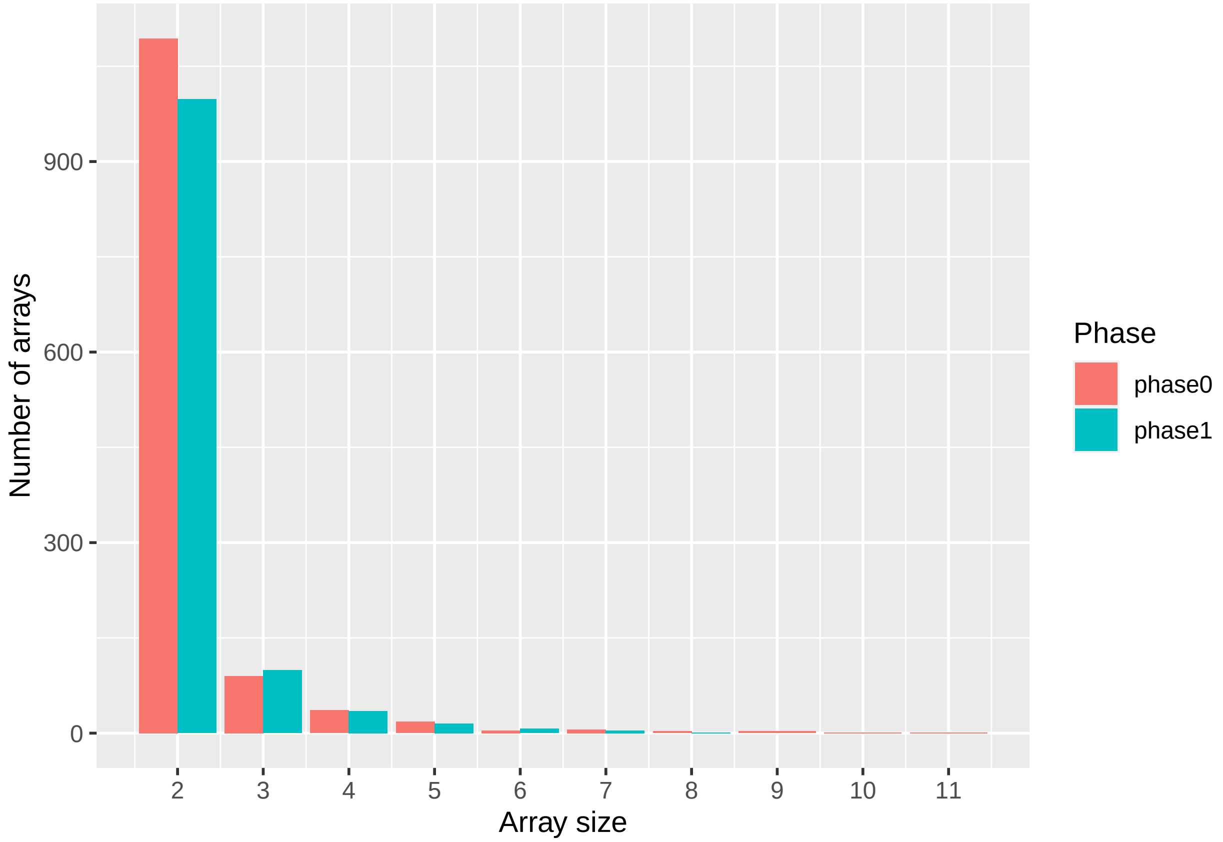


```
### GO terms

all_GOgenes <- names(GOList)

tgd <- runTopGoAnalysis(geneSet = str_extract(union(tandem0$gene1, tandem0$gene2), ".*(?=\\.)"),
                        allgenes = all_GOgenes,
                        GOList = GOList,
                        nodeSize = 100)

tandem_go <- tgdToTable(tgd, 50) %>% filter(Fisher.weight01 < 0.05)

tandem_go %>%
    knitr::kable(digits = 20)
```

GO.ID	Term	Annotated	Significant	Expected	Fisher.weight01
GO:0055114	oxidation-reduction process	1937	345	180.24	0.000e+00
GO:0016125	sterol metabolic process	208	77	19.35	0.000e+00
GO:0016132	brassinosteroid biosynthetic process	100	40	9.30	2.100e-16
GO:0045490	pectin catabolic process	151	49	14.05	1.900e-15
GO:0005975	carbohydrate metabolic process	1403	214	130.55	3.400e-15
GO:0055088	lipid homeostasis	119	41	11.07	3.800e-14
GO:0048544	recognition of pollen	139	43	12.93	6.400e-13
GO:0042545	cell wall modification	219	53	20.38	6.100e-11
GO:0051707	response to other organism	1725	195	160.51	1.000e-10
GO:0007275	multicellular organism development	3141	237	292.27	2.900e-09
GO:0032259	methylation	330	39	30.71	6.300e-09

GO.ID	Term	Annotated	Significant	Expected	Fisher.weight01
GO:1901617	organic hydroxy compound biosynthetic pr...	360	92	33.50	6.200e-08
GO:0006950	response to stress	4302	427	400.30	4.600e-07
GO:0007165	signal transduction	1997	197	185.82	1.900e-06
GO:0006952	defense response	1678	177	156.14	2.500e-06
GO:0009056	catabolic process	1926	217	179.21	2.500e-06
GO:0006468	protein phosphorylation	2327	274	216.52	5.800e-06
GO:0006629	lipid metabolic process	1392	196	129.52	7.300e-06
GO:0009699	phenylpropanoid biosynthetic process	138	30	12.84	8.500e-06
GO:0014070	response to organic cyclic compound	376	57	34.99	9.900e-06
GO:0009813	flavonoid biosynthetic process	122	27	11.35	1.700e-05
GO:0009611	response to wounding	291	47	27.08	1.300e-04
GO:0032502	developmental process	3833	294	356.66	1.500e-04
GO:0008643	carbohydrate transport	110	23	10.24	1.700e-04
GO:0044265	cellular macromolecule catabolic process	786	49	73.14	4.000e-04
GO:0032787	monocarboxylic acid metabolic process	669	71	62.25	4.600e-04
GO:0006508	proteolysis	1080	101	100.49	5.300e-04
GO:0055085	transmembrane transport	1416	145	131.76	7.200e-04
GO:0000272	polysaccharide catabolic process	224	65	20.84	7.900e-04
GO:0010033	response to organic substance	2360	240	219.60	1.180e-03
GO:0006081	cellular aldehyde metabolic process	103	20	9.58	1.180e-03
GO:0008610	lipid biosynthetic process	858	128	79.84	1.390e-03
GO:0044042	glucan metabolic process	276	34	25.68	1.530e-03
GO:0071395	cellular response to jasmonic acid stimu...	147	18	13.68	1.900e-03
GO:1901565	organonitrogen compound catabolic proces...	971	84	90.35	2.110e-03
GO:0044248	cellular catabolic process	1454	124	135.29	2.590e-03
GO:0006464	cellular protein modification process	4150	381	386.15	3.270e-03
GO:0006865	amino acid transport	112	20	10.42	3.330e-03
GO:1901615	organic hydroxy compound metabolic proce...	586	140	54.53	5.260e-03
GO:0048443	stamen development	174	23	16.19	5.770e-03
GO:0009620	response to fungus	373	54	34.71	5.800e-03
GO:0098754	detoxification	102	18	9.49	5.890e-03
GO:0044036	cell wall macromolecule metabolic proces...	187	25	17.40	6.190e-03
GO:1901701	cellular response to oxygen-containing c...	799	86	74.35	8.940e-03
GO:0042537	benzene-containing compound metabolic pr...	115	19	10.70	9.710e-03
GO:0006721	terpenoid metabolic process	253	30	23.54	1.002e-02
GO:0031668	cellular response to extracellular stimu...	273	29	25.40	1.104e-02
GO:0044281	small molecule metabolic process	2037	180	189.54	1.215e-02
GO:0002229	defense response to oomycetes	102	17	9.49	1.282e-02
GO:0072522	purine-containing compound biosynthetic ...	123	9	11.45	1.458e-02

```
sessionInfo()
```

```
## R version 4.0.5 (2021-03-31)
## Platform: x86_64-w64-mingw32/x64 (64-bit)
## Running under: Windows 10 x64 (build 19043)
##
## Matrix products: default
##
## locale:
## [1] LC_COLLATE=English_United States.1252
## [2] LC_CTYPE=English_United States.1252
## [3] LC_MONETARY=English_United States.1252
```

```
## [4] LC_NUMERIC=C
## [5] LC_TIME=English_United States.1252
##
## attached base packages:
## [1] stats4    parallel  stats     graphics  grDevices utils     datasets
## [8] methods   base
##
## other attached packages:
##  [1] writexl_1.4.0               topGO_2.42.0
##  [3] SparseM_1.81                GO.db_3.12.1
##  [5] AnnotationDbi_1.52.0        graph_1.68.0
##  [7] viridis_0.6.1               viridisLite_0.4.0
##  [9] DESeq2_1.30.1               SummarizedExperiment_1.20.0
## [11] Biobase_2.50.0              MatrixGenerics_1.2.1
## [13] matrixStats_0.59.0          GenomicRanges_1.42.0
## [15] GenomeInfoDb_1.26.7         IRanges_2.24.1
## [17] S4Vectors_0.28.1            BiocGenerics_0.36.1
## [19] UpSetR_1.4.0                forcats_0.5.1
## [21] stringr_1.4.0               dplyr_1.0.7
## [23] purrr_0.3.4                 readr_1.4.0
## [25] tidyr_1.1.3                 tibble_3.1.2
## [27] ggplot2_3.3.4               tidyverse_1.3.1
##
## loaded via a namespace (and not attached):
##  [1] bitops_1.0-7           fs_1.5.0               lubridate_1.7.10
##  [4] bit64_4.0.5            RColorBrewer_1.1-2     httr_1.4.2
##  [7] tools_4.0.5            backports_1.2.1        utf8_1.2.1
## [10] R6_2.5.0               DBI_1.1.1              colorspace_2.0-1
## [13] withr_2.4.2            tidyselect_1.1.1       gridExtra_2.3
## [16] bit_4.0.4              compiler_4.0.5         cli_2.5.0
## [19] rvest_1.0.0            xml2_1.3.2             DelayedArray_0.16.3
## [22] scales_1.1.1           genefilter_1.72.1      digest_0.6.27
## [25] rmarkdown_2.9          XVector_0.30.0         pkgconfig_2.0.3
## [28] htmltools_0.5.1.1      fastmap_1.1.0          dbplyr_2.1.1
## [31] rlang_0.4.11           readxl_1.3.1           rstudioapi_0.13
## [34] RSQLite_2.2.7          generics_0.1.0         jsonlite_1.7.2
## [37] BiocParallel_1.24.1    RCurl_1.98-1.3         magrittr_2.0.1
## [40] GenomeInfoDbData_1.2.4 Matrix_1.3-2           Rcpp_1.0.6
## [43] munsell_0.5.0          fansi_0.5.0            lifecycle_1.0.0
## [46] stringi_1.6.2          yaml_2.2.1             zlibbioc_1.36.0
## [49] plyr_1.8.6             grid_4.0.5             blob_1.2.1
## [52] crayon_1.4.1           lattice_0.20-41        splines_4.0.5
## [55] haven_2.4.1            annotate_1.68.0        hms_1.1.0
## [58] locfit_1.5-9.4         knitr_1.33             pillar_1.6.1
## [61] geneplotter_1.68.0     reprex_2.0.0           XML_3.99-0.6
## [64] glue_1.4.2             evaluate_0.14          modelr_0.1.8
## [67] vctrs_0.3.8            cellranger_1.1.0       gtable_0.3.0
## [70] assertthat_0.2.1       cachem_1.0.5           xfun_0.24
## [73] xtable_1.8-4           broom_0.7.7            survival_3.2-10
## [76] memoise_2.0.0          ellipsis_0.3.2
```
